# Proteomic and Systems Biology Analysis of the Monocyte Response to *Coxiella burnetii* Infection

**DOI:** 10.1371/journal.pone.0069558

**Published:** 2013-08-21

**Authors:** Matt Shipman, Kirk Lubick, David Fouchard, Rajani Gurram, Paul Grieco, Mark Jutila, Edward A. Dratz

**Affiliations:** 1 Department of Chemistry and Biochemistry, Montana State University, Bozeman, Montana, United States of America; 2 Department of Veterinary Molecular Biology, Montana State University, Bozeman, Montana, United States of America; University of Pittsburgh, United States of America

## Abstract

*Coxiella burnetii* is an obligate intracellular bacterial pathogen and the causative agent of Q fever. Chronic Q fever can produce debilitating fatigue and *C. burnetii* is considered a significant bioterror threat. *C. burnetii* occupies the monocyte phagolysosome and although prior work has explained features of the host-pathogen interaction, many aspects are still poorly understood. We have conducted a proteomic investigation of human Monomac I cells infected with the Nine Mile Phase II strain of *C. burnetii* and used the results as a framework for a systems biology model of the host response. Our principal methodology was multiplex differential 2D gel electrophoresis using ZDyes, a new generation of covalently linked fluorescent protein detection dyes under development at Montana State University. The 2D gel analysis facilitated the detection of changes in posttranslational modifications on intact proteins in response to infection. The systems model created from our data a framework for the design of experiments to seek a deeper understanding of the host-pathogen interactions.

## Introduction

*C. burnetii* is a remarkably infective organism, requiring as little as one bacterium to cause infection (ID_50_ = 1–10) [1], [2]. It is readily aerosolized, can be pneumatically dispersed, is stable for long term storage and environmentally persistent, making it a potential bioterror threat [3], [4]. Initially classified as a *Rickettsia* family member, *C. burnetii* has recently been reclassified as a unique member of the γ-proteobacterium clade and has been shown to be most closely related to *Legionella pneumophila* [2], [5]. *C. burnetii* inhabits the monocyte phagolysosome in humans, and it's primary carbon sources are likely to be amino acids and peptide [2], [5], [6].

Q fever can present as either an acute or chronic infection. Acute infections typically progress within 20 days of exposure, often presenting with fever, muscle pain, and severe headaches [5]. Infections that persist for more than six months are considered chronic, and can lead to serious complications, including chronic fatigue and endocarditis [5]. Although acute Q fever can be cleared without treatment, the prognosis is greatly improved upon administration of antibiotics. In addition, the bacterium can persist *in vivo*, despite years of continuous antibiotic treatment [1]. Less than 2% of all acute cases of Q fever are fatal, however, >65% of chronic cases can be fatal [5], [6]. In addition, the chronic fatigue associated with Q fever can become debilitating [7].

One reason that improved detection and treatment of Q fever is important is that a person who clears acute Q fever can still retain a reservoir of *C. burnetii*, currently believed to lie in the bone marrow, and reacquire the chronic form of the disease years after the initial infection [1], [5], [8]. The acidic environment in the phagolysosome presents unique challenges to *C. burnetii*'s survival, and a better understanding of host interactions with *C. burnetii* could lead to improved treatment options and prognosis. *C. burnetii* is able to reconfigure the intracellular environment and evade or inhibit host cell defenses [9]–[11]. How *C. burnetii* regulates the intracellular environment to suit its needs is not well understood and how the host cell responds to *C. burnetii* infection is incompletely described. Therefore, we have undertaken a proteomic investigation of the response of Monomac I cells to *C. burnetii* infection, seeking to generate a better understanding the host cell mechanisms. We utilized multiplex, differential 2D gel electrophoresis, tandem MS/MS analysis, and bioinformatic tools to identify proteins that change expression in response to *C. burnetii* infection. We observed statistically significant differential expression of thirteen unique proteins, as well as several isoforms of these proteins. This information was used to guide literatures searches to provide a foundation for a systems biology model describing some of the major features of the response, and to serve as a framework for future investigations.

We utilized Monomac I cells, a cultured monocytic cell line rather than primary monocytes. The cell line facilitated the acquisition of the amounts of cells necessary to perform this investigation more readily than purifying primary monocytes. In addition, the consistent genetic background of the cell line facilitated analysis, and enabled a consistent experimental framework that would be difficult to obtain from primary monocytes, acquired from multiple donors. We infected Monomac I cells with phase II *C. burnetii*, an avirulent laboratory strain rather than a pathogenic phase I strain. Heinzen's group recently found that phase I and phase II *C. burnetii* behave similarly in both primary monocyte-derived macrophages and cultured THP-1 cells, although phase II does have an attenuated LPS response [12]. The conditions within the phagolysosomal replicative vacuole have also been reported to be similar between phase I and phase II *C. burnetii* [12], although differences in the delivery of cathepsins to phase I and phase II *C. burnetii* infected phagolysosomes have been reported [13]. While the cited comparision of Phase I and Phase II *C. burnetii* [12] does not specifically address their effects on Monomac I cells, it does suggest that cultured monocytic cells can provide a good surrogate for primary monocytes, when examining the host cell responses to *C. burnetii* infection. The cells and conditions used in this investigation represented a favorable choice of systems for this initial investigation, and the results can serve as a basis for investigations of the monocyte- *C. burnetii* host-pathogen response in the future, using primary monocytes and the select agent.

## Methods

### Sample preparation

Monomac I cultures were grown and infected with phase II *Coxiella burnetii* (Ninemile strain originally provided by R. Heinzen, NIH, Rocky Mountain Laboratory) as previously described [14]. Final cell counts were approximately 2×10^8^ per sample, the cells were pelleted by centrifugation (5 minutes at 1000×g, 4°C) and the growth media discarded. Samples were centrifuged an additional 5 minutes (1000×g, 4°C), and any remaining growth media was removed by pipette. The entire cell sample was resuspended to 4 mL final volume, using monocyte extraction buffer (detailed in Methods S1) [15]–[17] and mixed thoroughly. The experimental flow for this investigation is diagrammed in figure S1.

Lysis protocols were adapted from published accounts [13], [16]. Briefly, samples were lysed on ice in 1 ml aliquots using a hand-driven, tight-fitting Dounce homogenizer. Four rounds of sonication were carried out on ice and a freeze thaw cycle to −80°C was followed by two rounds of sonication on ice [15], [18] (as described in Methods S1). Samples were held on ice for at least 1 minute between rounds of sonication to decrease the temperature and lysis was determined by cell counting in a hemacytometer.

The four aliquots were subjected to high speed centrifugation for 45 minutes at 100,000×g and 4°C, using a TSL100 ultracentrifuge and a TL-55 rotor (Beckman) to pellet membranes and organelles. The supernatant, containing the soluble cytosolic fraction and some luminal contents of organelles, was transferred to four 1.5 mL microfuge tubes on ice, for TCA precipitation, prior to dissolving in labeling buffer (as described in Methods S1).

The membrane fraction pellets, containing the plasma and organellar membranes, were stored at −80°C until further processing [19]. The lysed and frozen membrane fractions samples were thawed on ice, resuspended in 30 mM Tris pH 8.5, centrifuged at 100,000×g for 45 minutes at 4°C, and the supernatant discarded. The washed membrane pellet was dissolved in labeling buffer (see above), including 4% (w/v) CHAPS + 1% (w/v) ASB-14 (Calbiochem, research grade) [20]. Like aliquots were pooled, the protein content quantified by the Bradford assay (Biorad, 500-0006, Hercules CA) and stored at −80°C. See methods S1 for details.

### Labeling reactions and separation conditions for multiplex 2DE

100 ug of protein per channel (control and experimental) were labeled with different colored ZDyes [21]–[23], and reciprocal color labeling was carried out on duplicate samples, as described in more detail in methods S1. The samples were brought to 5 mg/mL final concentration with pH 8.5 labeling buffer, containing detergent(s) appropriate for the sample type.

Samples were labeled with 4 nmol of dye/reaction added to each protein aliquot and each aliquot was labeled separately. Samples were reacted for 30 minutes in the dark, followed by quenching with 10 mM lysine for 10 minutes. The quenched fractions, labeled with differently colored Zdyes, were combined appropriately, and DTT (20 mM final concentration, Biorad, electrophoresis grade), 0.5% (v/v) carrier ampholytes (GE Healthcare), labeling buffer to 450 uL final volume and 3 uL of 0.03% (w/v) bromophenol blue were added. Isoelectric focusing (IEF) was carried out using 24 cm immobilized pH gradient (IPG) strips pH 3–11NL (GE Healthcare). The samples were loaded onto IPG strips via active rehydration, at 50 V, 20°C for 14–16 hours. After loading, the strips were run as shown in table S1, removed from the IEF cell within one hour of the completion of the high voltage step, and stored at −80°C until the 2nd dimension separation.

Acrylamide gradient gels (9.5–16% for membrane samples and 9.5–18% for soluble fraction samples) were cast using 1.5 mm spacer plates and a GE Healthcare casting chamber. Prior to loading the gels, IPG strips were thawed, then reduced and alkylated as described in Methods S1 [24]. IPG strips were loaded onto 2nd dimension gels under a layer of 0.5% (w/v) agarose (Biorad, electrophoresis grade) with a trace of bromophenol blue (Biorad, electrophoresis grade), and run in a Dalt12 tank (Amersham/GE Healthcare) overnight in 1× running buffer at 16°C in the dark at ∼4 W/gel. Gels were fixed and washed as described in Methods S1 and scanned using a Typhoon Trio gel scanner (GE Healthcare), at 200 um resolution with laser excitation at 488 nm, 532 nm, and 633 nm and with the PMT voltage set to just below the threshold of pixel saturation.

### Gel analysis

Images were uploaded into the Progenesis software package (v.2, Nonlinear Dynamics), using the setting for multiple dyes without DIGE structure. The control and experimental images were each normalized by the total intensity in the analyzed area of each gel. During the analysis we recognized that a common method of analyzing multicolor differential 2D gel data, using the pooled internal standard for intensity normalization was leading to a higher statistical significance for differences between control and experimental images than was justified by the data. This effect was due to a lack of statistical independence between control and experimental datasets, when they were each normalized by the pooled internal standard, due to correlation of the data in the normalization as had been previously reported [25]–[27]. Thus in the final data analysis, only the ratios between the control and experimental images were utilized. The control and experimental images for the biological and technical replicates, under a particular test condition, were analyzed as one experiment. A reference image was chosen that had the highest apparent spot number counts, and the best qualitative appearance with respect to spot separation. Gel spot patterns were aligned using both manual and autovectors and the alignments were manually validated.

Three biological replicates were analyzed for each experimental condition and control. A minimum of 2 technical replicates were used for each labeling condition (with forward and reverse dye color swap labeling) in a particular biological replicate, for a minimum of 4 gel images/biological replicate (as described further in Methods S1). To be selected for further analysis, a protein spot required a p-value of <0.05, for the difference between control and experimental intensities and a power score of >0.8. The data was exported to an Excel file for the spots passing this phase of statistical analysis. The spot number and normalized spot volume data was formatted in Excel and then uploaded to SPSS v.16. Changes in the normalized spot volumes between experimental and control images were evaluated with a mixed linear model [25], as described in methods S1 and shown in table S2. Briefly, in the mixed linear model used, the technical replicates were grouped into their appropriate biological replicates. Biological replicates were grouped by test condition (control or experimental) and a two-way ANOVA was performed to obtain statistical calculations. For the final decision on the differential regulation of protein spots, a p-value from the mixed linear model was required to be <0.05 with an observed power score >0.7.

### Spot picking, in-gel digestion, and MS anaysis

Gels utilized for spot picking and MS analysis were stained with the Blue Silver formulation of Coomassie blue (described in Methods S1). A spot map was generated from Progenesis analysis of differentially regulated spots and a unique label was established for each picked spot. Spots were picked manually on a light box in a positive pressure, HEPA filtered hood. In-gel digestion protocols were adapted from Barry [28], [29]. Gel pieces were macerated with a clean forceps, washed until no longer blue, and then dried completely in a Speed-vac (see Methods S1 for further details). Following drying, the gel pieces were rehydrated with Promega modified porcine trypsin solution [12.5 ng/uL in 25 mM NH_4_HCO_3_/10% (v/v) acetonitrile pH 8.0] [28]–[30] using enough solution to cover the pieces with ∼3× their dry volume. Gel pieces were rehydrated on ice for 30 minutes, and excess trypsin solution was removed. The gel pieces were covered with 25 mM NH_4_HCO_3_/10% (v/v) acetonitrile pH 8.0 [30] and incubated overnight at 37°C. The digests were centrifuged briefly, 100 uL of nanopure water was added, vortexed for 10 minutes, and placed in a sonicating bath for 5 minutes. Digests were transferred to Eppendorf tubes containing 10 uL of 50% (v/v) acetonitrile/5% (v/v) formic acid (Fisher Scientific, ACS grade).

The peptides were extracted three times using fresh 50% (v/v) acetonitrile/5% (v/v) formic acid (as described further in Methods S1), reduced to ∼20 uL final volume in a Speed-vac and either stored at −80°C or used immediately for MS analysis. Digests were analyzed using an Agilent ChipLC system with a 150 mm separation column (part# G4240-62002) and Agilent XCT Ultra Ion trap mass spectrometer, using: Solvent A [95% (v/v) water (Fisher Scientific, HPLC grade)/5% (v/v) acetonitrile (Fisher Scientific, HPLC grade)/0.1% (v/v) formic acid] and Solvent B [5% (v/v) water/95% (v/v) acetonitrile (HPLC grade)/0.1% (v/v) formic acid]. Completed runs were analyzed using Bruker Daltonics Data Analysis software.

### Bioinformatics

Four bioinformatics tools were used for analysis of the mass spectrometer data. Mascot (licensed in-house at Montana State University) [31] and the X!Tandem, X!Hunter and X!Tandem P3 algorithms from www.thegpm.org [32]–[35] were utilized. One missed cleavage by trypsin was allowed and the parent ion mass tolerance was set to 0.8 Da. The NCBInr human database was searched in Mascot and an MS/MS tolerance of 0.3 Da was used, with cysteine carbamidomethylation, deamidation and methionine oxidation included as variable parameters for +2 and +3 ions. For both X!Hunter and X!Tandem P3 searches, all available human databases were searched and default parameters were used, with the exception of the 0.8 Da parent ion mass tolerance. For X!Hunter only +2 charges were searched. The *C. burnetii* database was searched in X!Tandem. Protein identifications that were not keratin or trypsin, and were the highest ranked protein identification that most closely matching the observed molecular weight and pI were accepted. A minimum of two unique, statistically significant peptides were required for a protein identification. Mascot protein scores are reported as the sum of peptide scores from all statistically significant peptides.

Deamidations were a commonly detected posttranslational modification, perhaps due to the observation that standard protein digestion techniques can cause partial deamidations *in vitro* [36]. We cannot distinguish *in vivo* deamidation from *in vitro* deamidation with the methods used in this investigation. We scanned for common phosphorylation neutral loss masses of 49 and 98 Da (for +2, and +1 ions respectively). No phosphorylation sites were identified by bioinformatics analysis, however phosphorylation can be difficult to detect by the collision induced dissociation methods employed.

## Results

**Figure 1:**
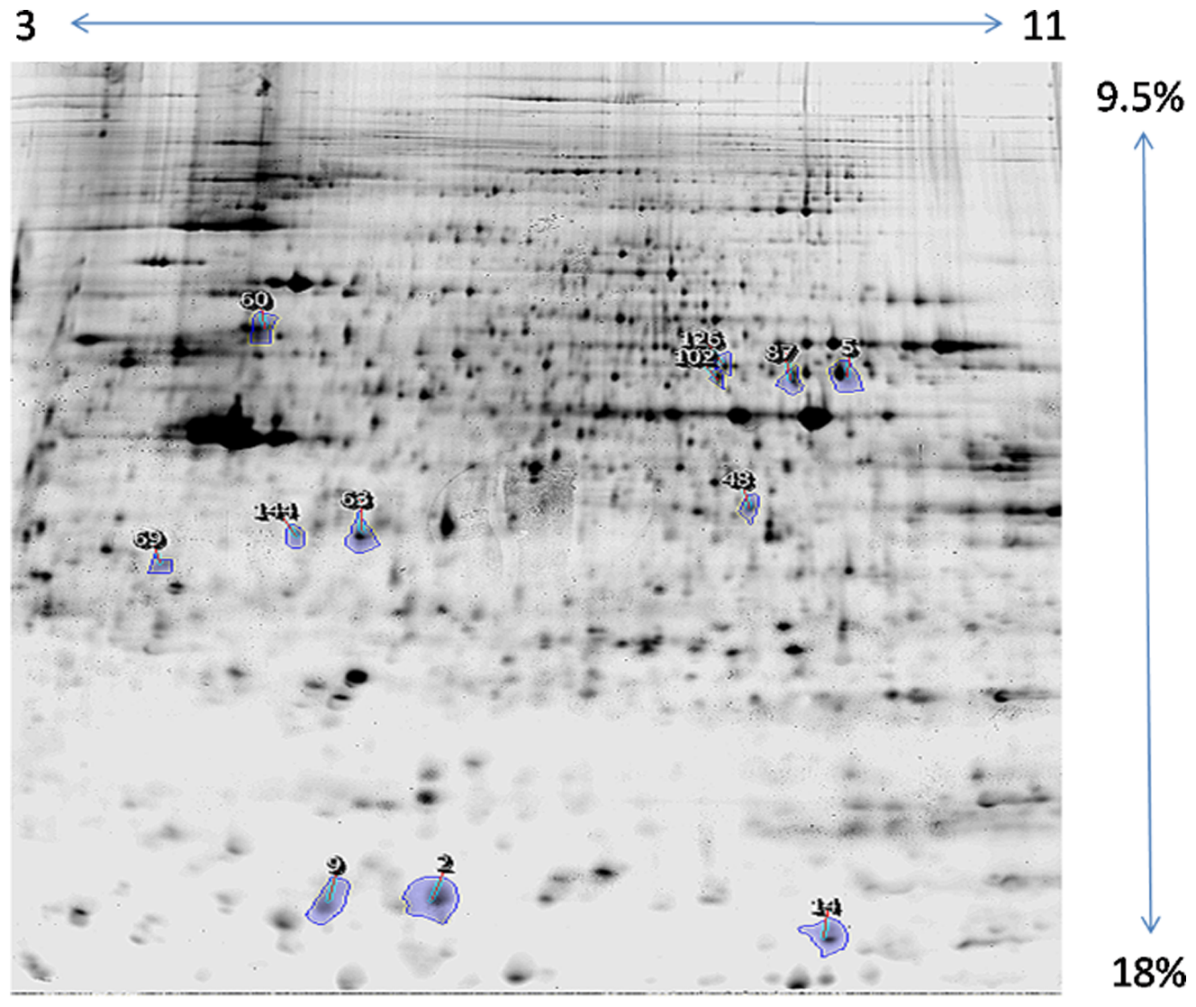
Soluble protein fraction, 96 hr postinfection. Progenesis master image. Where outlined, blue numbered spots were determined to be differentially expressed in a statistically significant manner, following image analysis and offline statistical analysis. The analysis included 3 biological replicates, with 6 technical replicates per biological replicate, including reciprocal Zdye color labeling, as described in the text. See tables 1 and 2 for statistical analyses and protein identifications.

**Figure 2:**
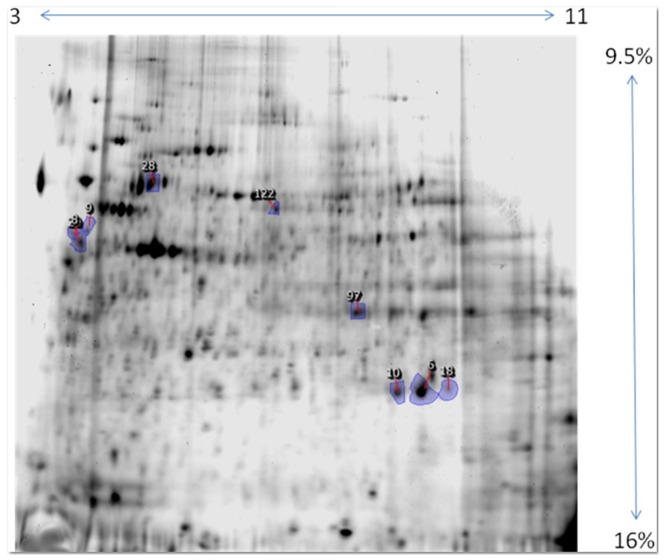
Membrane protein fraction 96 hr postinfection. Progenesis master image where outlined, blue numbered spots were differentially expressed in a statistically significant manner, following image analysis and offline statistical analysis. The analysis was for 3 biological replicates, including 6 technical replicates per biological replicate with reciprocal Zdye color labeling, as described in the text. See tables 1 and 2 for statistical analyses and protein identifications. Note that spot 20 (indicated by the arrow) is partially obscured by spot 8.

We studied samples of Monomac I cells infected with phase II *C. burnetii* at a multiplicity of infection of 200∶1. Aliquots were taken at 24, 48, and 96 hrs. postinfection, and controls were sham infected with PBS. Three biological replicates were obtained and a total of four to six technical replicates were performed for each biological replicate (as described in Methods). Representative gel images are shown in figures 1 and 2. Gel spots that differed significantly between the experimental and control samples were identified as described in the Methods section. One spot changed expression in a statistically significant manner at 48 hrs. postinfection (figure S2), and 21 spots exhibited differential expression in the soluble and membrane fractions at 96 hrs postinfection. Proteins that exhibited differential expression were identified following MS/MS analysis, using the four different bioinformatics tools described in Methods.

The final list of identified proteins as well as associated statistical and bioinformatic data is shown in tables 1 and 2. Typically we obtained >20% protein sequence coverage for each protein identification. False discovery rates (FDR) were determined to be <1% for X!Hunter and <1.5% for P3 for all spots analyzed. In most cases, all four bioinformatics tools gave the same protein identifications, although with somewhat different peptide lists, reflecting differences in the algorithms and treatment of posttranslational modifications. Many of the changing proteins were found as distinct spots in two or more isoforms on 2D gels, as described later. The 22 identifiable protein spots that were found to change significantly were accounted for by isoforms of 15 different proteins. No significant hits were identified as *C. burnetii* proteins with one exception. This exception occurred in a protein spot that was also identified as human Hsp60. When searched against a *C. burnetii* database, X!Tandem identified the spot as GroEL. When searching both human and *C. burnetii* databases on X!Tandem simultaneously, human Hsp60 was the best hit identified, supporting the hypothesis that *C. burnetii* GroEL was a misidentification, due to homology with Hsp60. While bacterial proteins must certainly be present, the experiments lacked the sensitivity to identify changes in their expression using the conditions employed.

**Table 1:** Differentially regulated protein identifications and statistical data.

			Nested ANOVA		
Test condition	Spot #	Fold change	p-value	Power score	ID
48 hr sol	10	2.1	0.0101	0.921	Mn-containing Superoxide Dismutase isoform A
96 hr sol	2	4.1	0.0026	0.998	S100 Ca Binding Protein A9
	5	3.0	0.0147	0.861	Visfatin/PBEF
	9	2.7	0.0097	0.927	S100 Ca Binding Protein A9
	14	2.6	0.0156	0.850	S100 Ca Binding Protein A8
	48	1.7	0.0180	0.820	Transaldolase 1
	60	1.6	0.0154	0.853	Chaperonin/Hsp60
	63	1.6	0.0047	0.985	PPA1Cytosolic Inorganic Pyrophosphatase
	69	1.6	0.0174	0.829	Microtubule associated protein EB1
	87	1.5	0.0172	0.831	Fascin 1
	102	1.5	0.0231	0.763	Leucine Aminopeptidase 3
	126	1.4	0.0127	0.887	Leucine Aminopeptidase 3
	144	1.3	0.0003	1.000	Pyrophosphatase 1
96 hr mem	6	3.1	0.0091	0.934	Mn containing superoxide dismutase
	8	2.4	0.0084	0.944	Vimentin
	9	2.4	0.0104	0.917	Vimentin
	10	2.3	0.0006	1.000	Rab7
	18	−2.0	0.0040	0.990	Rab7
	20	1.9	0.0057	0.975	Vimentin
	28	1.6	0.0195	0.803	Chaperonin/Hsp60
	97	−1.3	0.0119	0.898	Enoyl CoA Hydratase 1
	122	1.2	0.0141	0.869	Aldehyde Dehydrogenase 2

Regulated proteins from *C. burnetii* infected monocyte samples compared to uninfected monocytes. Spot numbers indicate the spot numbers in the gel images. Selection criteria were p<0.05 and power score >0.7 for the differences in protein abundance in control compared to infected conditions. Fold change refers to the direction and magnitude of protein expression changes in the infected samples compared to the uninfected controls.

**Table 2:** Bioinformatic data for differentially expressed monocyte proteins.

Test condition	Spot #	ID	Mascot Score	Mascot # Unique Peptides	Mascot % Coverage	NCBI Accession #	X!Hunter Score	X!Hunter # Unique Peptides	X!Hunter % Coverage	X!Hunter FDR (%)	X!Hunter/P3 Uniprot Accession #	P3 Score	P3 # Unique Peptides	P3 % Coverage	P3 FDR (%)
48 hr sol	10	Mn-containing Superoxide Dismutase isoform A	519	4	23.4	30841309	−43.6	6	24.8	0.22	P04179	−31.1	5	23.0	1.07
96 hr sol	2	S100 Ca Binding Protein A9	212	3	31.6	4506773	−40.7	4	28.9	0.02	P06702	−17.4	3	31.6	0.95
	5	Visfatin/PBEF	767	10	30.3	5031977	−59.7	7	15.5	0.37	P43490	−67.4	10	18.5	1.23
	9	S100 Ca Binding Protein A9	198	3	44.7	4506773	−40.1	4	30.7	0.20	P06702	−28.2	4	30.7	1.00
	14	S100 Ca Binding Protein A8	728	9	48.4	21614544	−31.2	5	34.4	0.41	P05109	−33.5	7	39.8	1.17
	48	Transaldolase 1	456	7	21.1	5803187	−56.1	7	19.3	0.46	P37837	−43.8	7	16.6	1.18
	60	Chaperonin/Hsp60	722	12	26.2	31542947	−85.1	10	17.5	0.23	P10809	−118.9	12	21.5	1.10
	63	PPA1Cytosolic Inorganic Pyrophosphatase	589	7	33.8	4583153	−84.1	9	30.8	0.34	Q15181	−96.1	9	24.2	1.12
	69	Microtubule associated protein EB1	447	7	31.7	6912494	−86.4	8	27.6	0.24	Q15691	−71.9	9	19.8	1.46
	87	Fascin 1	442	8	22.2	4507115	−87.4	9	20.5	0.21	Q16658	−61.3	9	17.6	1.20
	102	Leucine Aminopeptidase 3	857	8	20.9	37588925	−83.4	13	24.9	0.27	P28838	−103.4	21	30.1	1.20
	126	Leucine Aminopeptidase 3	869	14	28.3	37588925	−84.9	12	24.3	0.22	P28838	−131.7	16	26.6	0.79
	144	Pyrophosphatase 1	278	5	33.8	4583153	−71.1	8	30.8	0.32	Q15181	−76.2	11	30.8	0.99
96 hr mem	6	Mn containing superoxide dismutase	397	3	22.1	30841303	−77.9	13	32.4	0.32	P04179	−58.4	10	34.2	1.42
	8	Vimentin	1761	21	35.0	37852	−168.3	28	41.6	0.24	P08670	−149.1	24	32.4	0.63
	9	Vimentin	1146	13	21.9	37852	−148.2	22	35.0	0.18	P08670	−149.5	19	28.1	0.97
	10	Rab7	560	6	51.6	34147513	−44.6	7	30.9	0.32	P51149	−73.7	6	29.5	1.05
	18	Rab7	240	4	30.7	34147513	−44.3	5	31.9	0.37	P51149	−35.2	4	27.1	1.29
	20	Vimentin	963	12	32.2	37852	−137.4	19	44.4	0.46	P08670	−101.6	15	24.9	1.18
	28	Chaperonin/Hsp60	1316	15	32.8	31542947	−127.2	10	30.7	0.32	P10809	−190.7	19	28.4	0.78
	97	Enoyl CoA Hydratase 1	1591	12	29.5	70995211	−47.0	5	23.2	0.36	Q13011	−93.8	19	33.2	0.79
	122	Aldehyde Dehydrogenase 2	1250	14	27.3	25777732	−117.1	20	32.5	0.15	P05091	−156.7	31	38.7	1.01

Results of the bioinformatic analysis for protein identified as differentially expressed. See table 1 for statistical analysis of the changes in individual protein spots from the gel data. The scores for the various bioinformatic tools were calculated by their respective software, as was %FDR for X!Hunter and X!Tandem P3 (referred to as P3 in the table). The number of unique peptides and the %coverage was calculated, following manual validation of the peptides from each of the tools. Accession numbers for Mascot are for the NCBInr database, the accession numbers for X!Hunter and P3 are for the IPI database.

### Differentially expressed proteins

We observed changes in two isoforms of Rab7 in the membrane fraction of 96 hr infected samples, one upregulated ∼2-fold and one downregulated ∼2-fold (spot numbers 10 and 18 in figure 2), illustrating the isoform resolution capabililties of 2D gel electrophoresis. The Rab7 protein is involved in maturation of phagosomes [37]–[39]. Manganese superoxide dismutase (MnSOD) was observed to be upregulated 2.1 fold in the soluble fraction at 48 hrs postinfection, as well as 3.1 fold in the membrane fraction at 96 hrs postinfection. SODs are highly conserved proteins that convert superoxide anions into hydrogen peroxide. Bacteria contain one to three different types of SOD enzymes [40], and these contribute to the virulence of many human pathogens [41]. However, the bioinformatics analysis establishes that we are observing human mitochondrial and not bacterial MnSOD in the experiments described.

Vimentin was observed to be upregulated 2.4 fold in the membrane fraction 96 hr postinfection. Vimentin is an intermediate filament protein, and is highly abundant in monocytes [42], [43]. S100A8 and A9 are strongly upregulated in the soluble fraction of 96 hr postinfection samples. These proteins are expressed in innate immune cells, including monocytes as either homo- or heterodimers [44], and are markers for macrophage differentiation [45]. Visfatin was observed to be upregulated 3 fold in the soluble fraction at 96 hrs postinfection. Visfatin [also known as pre-B cell colony enhancing factor and nicotinamide phosphoribosyltransferase (NAMPT)], is a highly conserved cytokine to which numerous functions have been ascribed [46]–[50]. Although bacteria do contain a visfatin homolog, bioinformatic analyses indicate that the upregulated visfatin detected in our samples is of human origin. We observed an upregulation of EB1, a microtubule associated protein, by 1.6 fold in the soluble fraction of 96 hr postinfection samples. EB1 was recently reported to regulate phagosome maturation [51]. We observed an upregulation of Hsp60 in the soluble fraction samples taken at 96 hrs postinfection. Hsp60 is a chaperone protein, that has been implicated in immune responses [52].

Enoyl CoA Hydratase 1 (ECH) was downregulated 1.3 fold in the membrane fraction of 96 hr postinfection samples. ECH controls the rate limiting step in fatty acid β-oxidation [53] and is crucial for oxidative energy generation. We observed Leucine amino peptidase 3 (Lap3) being upregulated 1.4–1.5 fold (different isoforms) in the soluble fraction at 96 hrs postinfection. Lap3 has recently begun to attract interest for its cysteine protease activity [54]–[56]. Transaldolase (TAL) was upregulated 1.7 fold in the soluble fraction of *C. burnetii* infected samples at 96 hrs postinfection. Transaldolase is a key enzyme in the nonoxidative portion of the pentose phosphate pathway (PPP) [57], [58]. We observed that aldehyde dehydrogenase 2 (Aldh2) is upregulated 1.2 fold in the membrane fraction of 96 hr postinfection samples. Aldh2 catalyzes the conversion of aldehydes to carboxylic acids in mitochondria. Inorganic pyrophosphatase (PPA1) was observed to be upregulated by 1.3–1.6 fold in the soluble fraction of samples at 96 hrs postinfection. Pyrophosphatases catalyze the conversion of pyrophosphates to inorganic phosphate, which is important for cellular energy to pull reactions forward that yield AMP and pyrophosphate [59]. Bioinformatic analyses confirm that the PPA1 we observe to be upregulated is of human origin.

### Systems biology models

**Figure 3:**
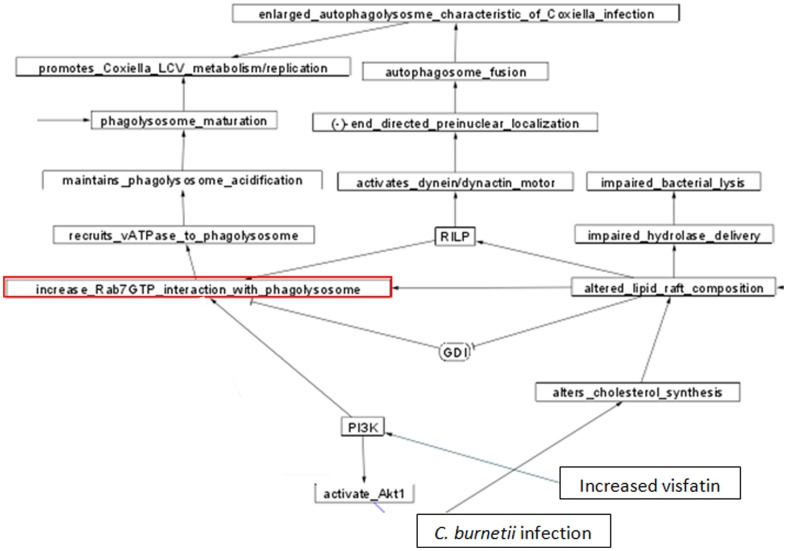
Detail of regulation and proposed Rab7 functions from figure S3. Detail from figure S2, showing the regulation of Rab7 GTP activity and the various potential roles of Rab7GTP. *C. burnetii* infection alters cellular cholesterol synthesis and alters lipid raft composition. Altered lipid raft composition impairs hydrolase delivery and bacterial lysis, as well as favoring recruitment of Rab7 to the phagolysosomal membrane. Proposed functional consequences of maintaining Rab7 recruitment to the phagosomal membrane include maintenance of vATPase at the phagolysosomal membrane, which would act to maintain acidification of the phagolysosome, required for metabolic and replicative processes of *C. burnetii*. RILP =  Rab Interacting Lysosomal Protein. GDI = Guanine nucleotide Dissociation Inhibitor.

We surveyed the literature to more fully understand what is known about the functions of the proteins that change expression during infection with *C. burnetii* and to seek to fit the proteins into a signaling and metabolic framework. Figure S3 shows the systems biology model of the data which is presented in the supplements, as it is too large to fit in a text figure. Figure S3 shows known and/or predicted connections between various proteins in the monocyte. An example of a more detailed section from S3 is shown in figure 3 that includes the role of the activated Rab7 protein, as deduced from the literature. The players in the model and their interactions as found in the literature are considered in the Discussion section.

**Figure 4:**
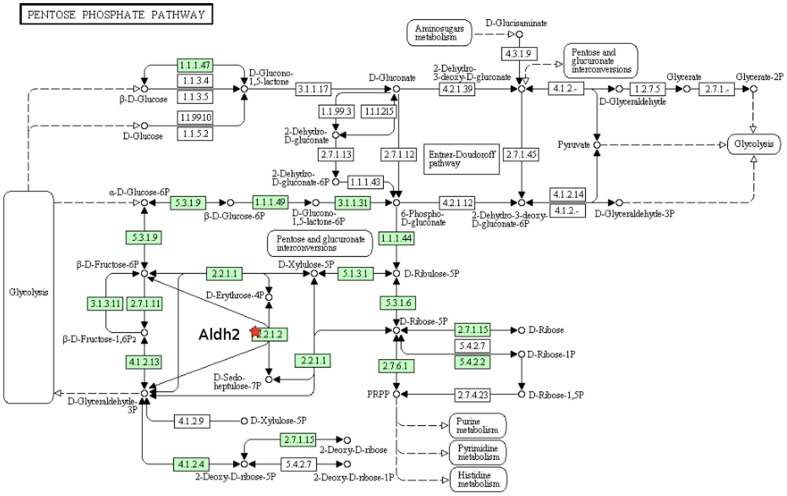
DAVID analysis of the linkages of aldehyde dehydrogenase 2 (Aldh2). Diagram from the KEGG pathway analysis database produced by DAVID^61^. The red star indicates Aldh2 (EC 1.2.1.3). The diagram shows the involvement of aldehyde dehydrogenase 2 (Aldh2) in Valine, Leucine, and Isoleucine degradation.

**Figure 5:**
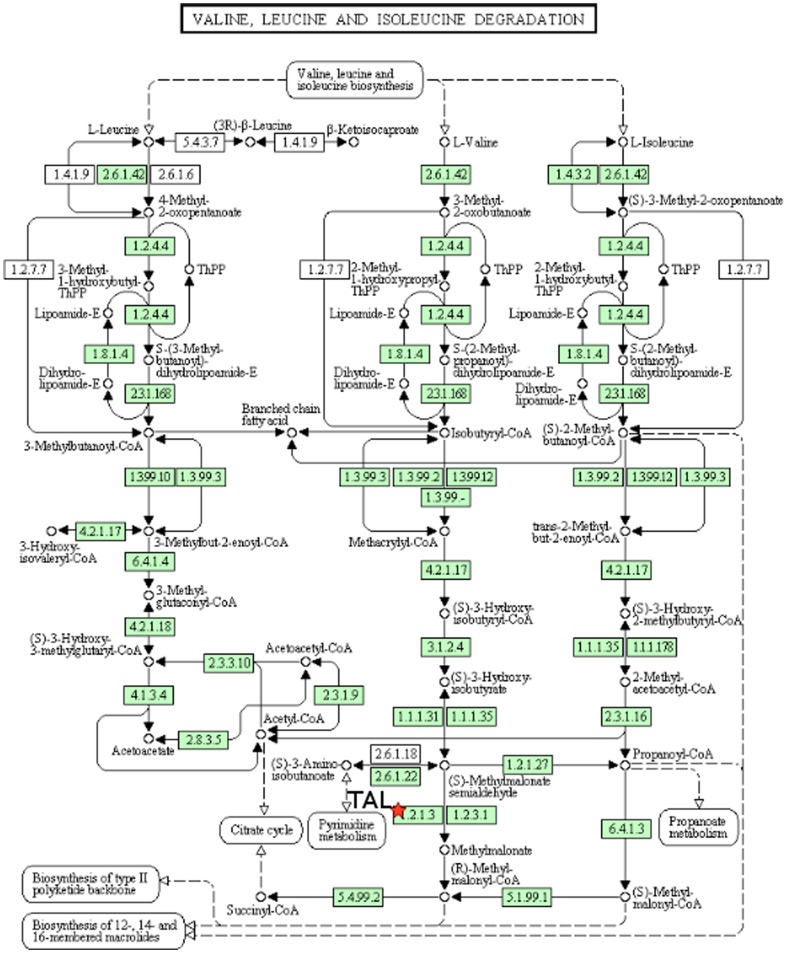
DAVID analysis of the linkages of transaldolase 1 (TAL1). Diagram from the KEGG pathway analysis database produced by DAVID^61^. The red star indicates TAL1 (EC 2.2.1.2). The diagram shows the involvement of transaldolase 1 in the pentose phosphate pathway.

We also utilized the GOEAST [60] and DAVID [61], [62] systems biology tools to evaluate the functions and relations of the proteins that were found to change expression upon *C. burnetii* infection. Figure 4, derived from DAVID, shows metabolic pathways related to Aldh2, which is an important step in branched chain amino acid metabolism. Figure 5, also derived from DAVID, shows the position of transaldolase in the pentose phosphate pathway (PPP). Together, the changes observed in metabolic protein expression suggest that the host cell metabolism is being altered in response to infection. In general, DAVID provided greater molecular detail provided for the various pathways under analysis, whereas GOEAST provided more of an overview. The manually constructed model was the most focused of the models, looking primarily at immunological functions that are most likely to be relevant to the monocyte response to *C. burnetii* infection. The manually generated model serves as a framework from which to derive mechanistic hypotheses about the system that may not be obvious when considering each regulated protein individually, as well as suggesting interconnections between regulated proteins, as described in the Discussion.

## Discussion

### Visfatin

We observed a pronounced upregulation of visfatin after infection. Visfatin is involved in catalyzing the conversion of nicotinamide to NAD, a central electron carrier in many metabolic reactions [47]. Inhibition of visfatin has been shown to reduce the intracellular availability of NAD and to reduce the secretion of TNFα, as well as other proinflammatory cytokines (e.g. IL-6) in a dose-dependent manner, both *in vivo* and *in vitro* [47]. The upregulation of visfatin in our experiments, suggests that a corresponding increase in NAD may have occurred, as indicated in figure S3. Visfatin mRNA expression levels can be modulated by several cellular factors, including activated NF-κB [49]. Experiments of Kendal and Bryant-Greenwood suggest that several markers of inflammation may regulate expression of visfatin and/or control its secretion [49].

Adya et al., have recently demonstrated that human endothelial cells upregulate mRNA expression for monocyte chemoattractant protein 1 (MCP1) in response to visfatin exposure, in a dose-dependent manner [46]. Further it was suggested that activated PI_3_K and NF-κB pathways are involved and necessary for MCP1 mRNA upregulation [46]. PI_3_K is also involved in phagosome maturation and autophagic vacuole maturation [37], [63] (see Figure 3), suggesting a possible interaction between Rab7-mediated phagolysosomal maturation (to be discussed later), and the downstream effects of visfatin. PI_3_K can activate the Akt pathway [46] which is known to be involved in *C. burnetii*'s inhibition of apoptosis in infected monocytes [64] (see figure S3).

Jia et al., showed that loss of visfatin expression abrogates LPS-induced inhibition of apoptosis in neutrophils, a cell type that is constitutively apoptotic and where apoptosis can be inhibited by LPS activation of TLRs [48]. Jia et al., further demonstrated that visfatin expression alone is sufficient to inhibit apoptosis in unstimulated neutrophils, that visfatin is secreted and its inhibition of apoptosis is at least partially due to its actions as a cytokine [48]. The antiapoptotic activities of visfatin were shown to be due, at least in part, to inhibition of caspase 3 and 8 activity [48], and this may be related to the reported inhibition of host caspases by *C. burnetii* infection [64], as diagrammed in figure S3. Visfatin appears to be involved in a general response to pathological conditions [46], [50] and the interactions between visfatin and its effectors may drive a feed-forward protective mechanism, which could later be modulated/downregulated or could lead to a chronic inflammatory state.

### Manganese superoxide dismutase

Our results suggest that the elevated MnSOD that we observed following infection is an early cellular response to the host cell respiratory burst and the resulting increased levels of reactive oxygen species (ROS). Inflammatory signals, such as those triggered by infection, cause the production of ROS, including the superoxide anion, which are an important part of the microbicidal process. Infection by *C. burnetii* is known to produce a reduced respiratory burst compared to most other bacterial infections [65]. Higher levels of MnSOD produced after infection would be expected to help increase the ability of the host cells to resist the pathogenesis of the inflammation [66]. Increasing levels of MnSOD were also reported to cause an increase in the levels of glutathione, which is an important part of the machinery protecting the cell from ROS [67].

We observed that the change in MnSOD expression altered its subcellular localization from the soluble fraction (which can include luminal contents of organelles) at 48 hours p.i. to the membrane-bound fraction at 96 hrs p.i., as shown in Figure 2. We cannot determine the organellar localization of proteins from our experimental design and further work would be needed to establish the subcellular localization of MnSOD as a function of time after *C. burnetii* infection. The model indicates that MnSOD is one of several ROS-inhibiting activities in infected monocytes as are detailed below.

### Enoyl CoA hydratase

Observations in the literature and these experiments indicate that the mitochondrial protein ECH is downregulated in a variety of infectious conditions [68], [69]. ECH is the rate-limiting enzyme in beta oxidation and may be downregulated in order to reduce energy availability, as a means to reduce pathogen replication within cells. Shutting down beta oxidation would tend to reduce the availability of ATP and acetyl CoA (AcCoA), as diagrammed in figure S3. The two carbon acetyl compound carried by AcCoA is a central intermediate in energy metabolism that is derived from the breakdown of glucose, fatty acids, or ketogenic amino acids. AcCoA serves as the entry point into the Krebs cycle, which is a key component of aerobic energy generation, including the production of NADH and FADH_2_, which carry electrons to the respiratory electron transport chain.

The response of mouse liver cells to LPS stimulation was recently reported to induce steatosis, the excessive retention of lipids in the liver, which reduces the lipids available for energy generation [70]. Ohhira et al., reported that lipopolysaccharide stimulation of Toll-like receptors induced a reduction in the PPARα-related transcription regulation system, which includes ECH, and observed an ∼50% reduction in ECH mRNA expression in response to 2 hours of LPS stimulation [70]. In human glioblastoma cells treated with siRNA to knockdown ECH expression (30–45% reduction observed) and subsequently infected with measles virus (MV), it was reported that MV replication was significantly impaired, in some cases by nearly 100% [68]. These studies were extended to vesicular stomatitis virus and semliki forest virus, where similar effects on inhibition of viral replication were observed [68]. Watari et al., had previously demonstrated a similar down regulation of ECH following infection with MV, and further demonstrated that siRNA-induced knockdown of ECH mRNA expression impaired the replication of MV in monkey kidney cells [69].

It seems likely that a reduction in PPARα may be responsible for the reduction in ECH that we found in response to infection, in a similar manner to published reports [70], and summarized in the model in figure S3. The downregulation of ECH may be due to Hsp60 (or other effector) that is secreted in response to *C. burnetii* infection. Hsp60 can trigger TLR2/4 [100]–[101] and it is possible that secreted Hsp60 is acting on infected monocytes to trigger a downregulation of ECH expression.

In summary, our model predicts that a decrease in ECH expression reduces the availability of ATP, AcCoA and other metabolic intermediates, which may impair *C. burnetii* infection. A further possibility is that *C. burnetii* may be taking advantage of an increased level of lipids, that would be expected to result from the reduction in the rate of β-oxidation, to alter the lipid composition of phagolysosome membranes, and perhaps also alter the lipid composition of lipid rafts containing Rab7, as discussed further below [71].

### Calcium binding S100A8/9

We found that three S100A8/9 protein isoforms were upregulated by 2.5–4 fold at 96 hrs p.i., suggesting that the infected monocytes are in the early stages of differentiating into mature macrophages [45], in response to *C. burnetii* infection. The S100A8/9 proteins are upregulated during differentiation in the model illustrated in figure S3, to increase inflammation and to trigger responses that attract other immune cells. Wounds cause a rapid increase in S100A8/9 expression, before the appearance of inflammation, followed by disappearance of these proteins over time, supporting the conclusion that these are early acting proteins [72]. It is possible that S100A8/9 proteins are also being secreted as signals to attempt to recruit more macrophages, but we did not test the secretome in our analysis.

Secretion of S100A8/9 is typically preceded by activation of PKC [44], [45], and PKC can also initiate phosphorylation of GABA_A_ receptors to inhibit aspects of GABA_A_ receptor function [73]. Securinine, a GABA_A_ receptor antagonist, has been shown to accelerate killing of and inhibit infection by *C. burnetii* [14]. Activation of GABA_A_ receptors have been shown to downregulate or moderate the immune regulation of both monocytes and T cells [14], [74]–[76]. This suggests that activation of PKC, following the initial cellular responses to infection, may initiate a later acting downregulation of GABA_A_ receptor function, as part of a robust immune response. Future, targeted examination of PKC levels (and subtypes), as well as assessing the secretion of S100A8/9 and the activity of GABA_A_ receptors as a function of time, in response to *C. burnetii* infection, should be helpful to understand the interplay between these proteins.

### Vimentin

Our 2D gels resolved three separate protein spots, that were identified as vimentin isoforms (figure 2), and the isoelectric point shifts of these isoforms are consistent with the occurrence of three different phosphorylation states of vimentin. It has been reported that vimentin has a number of rapidly turned over phosphorylation sites that alter the dynamics of filament assembly [77]. Several phosphorylations were demonstrated by Eriksson et al., to regulate the assembly and disassembly of vimentin fibers [78]. We did not directly identify phosphorylation sites on vimentin in these experiments, however phosphorylation can be difficult to identify by the mass spectrometry methods used in this investigation.

One of the vimentin spots (spot 20) is found at an apparent molecular weight suggesting that it may be cleaved. Cleavage of vimentin is possibly due to caspase activity, which would suggest that the reported inhibition of caspase activation by *C. burnetii* [64] may not be fully effective by 96 hours in our experiments, using phase II *C. burnetii*. We propose that vimentin may take part in regulating the cytoskeletal arrangement of the monocytes after *C. burnetii* infection. Vimentin regulation of cytoskeletal dynamics may have implications for optimizing the cytoskeleton for autophagic vacuole delivery to the *C. burnetii* replicative vacuole, and the cellular localization of that vacuole, suggests that vimentin may be a potential drug target to inhibit *C. burnetii* infection.

Increased vimentin expression has been reported to occur during monocyte differentiation, and is important for monocyte proliferation [77]. Reduction of vimentin expression in BM2 cells suppressed proliferation and reducing vimentin levels also reduced ROS production [77]. Vimentin can be secreted by activated macrophages, and both PKC and TNFα are independently involved in controlling vimentin secretion [43]. It has also been suggested that vimentin is associated with the phagosome membrane [79], which is in keeping with changes in vimentin expression appearing in the membrane fraction of the monocytes. The upregulation of vimentin in response to *C. burnetii* infection suggests that the monocytes are being activated after infection to become macrophages and may have started differentiating at 96 hours postinfection (as summarized in figure S3).

### Aldehyde dehydrogenase 2

We found Aldh2 levels increase in response to *C. burnetti* infection. Aldh2 contains a series of thiol groups that are subject to oxidation by ROS, which inactivates the protein [80]. Wenzel et al., demonstrated that Aldh2 was inhibited in a dose dependant manner by oxidizing compounds, and that loss of Aldh2 results in a buildup of toxic aldehyde species, as well as activating NADPH oxidases, further increasing ROS production [81]. It was demonstrated that lipoic acid, naturally abundant in mitochondria, can reduce oxidized Aldh2 back to its active state, while glutathione (GSH) cannot [81]. Microarray analysis of oxidative stress in *Schistosoma mansoni* demonstrated that oxidative conditions cause a large upregulation (up to 4-fold) in mRNA for Aldh2 [82], although we are observing a much smaller protein upregulation [43]. This observed expression difference could simply reflect a different time course of Aldh2 activation in our experiments or may reflect the generally poor correlation between mRNA levels and protein expression levels [83], [84].

The lower increase in Aldh2 in *C. burnetii* infected samples, compared to the increase observed in *S. mansonii* mRNA experiments, might also indicate that there is less oxidative stress in our samples than occurred in the *S. mansonii* experiments. *C. burnetii* infections have been reported to induce a smaller oxidative burst in phagocytes, compared to several other infections [60], and the smaller change in the levels of Aldh2 in our samples may reflect this. We propose that Aldh2 is acting in an antioxidant capacity to protect proteins in *C. burnetii* infected monocytes from products of ROS and RNS, that arise as a result of infection, as indicated in figure S3. It was reported by Li et al., that overexpression of an Aldh2 transgene inhibited ROS-induced caspase 3-mediated apoptosis, in addition to reducing Erk1/2 and p38 activation, in response to increased Aldh2 expression [85]. Lipoic acid provides an *in vivo* mechanism for reduction of oxidized Aldh2 thiols, to maintain Aldh2's functionality [81]. A prediction that arises from our model (figure S3) is that oxidative stress, in response to infection by *C. burnetii*, could trigger an increase in lipoic acid production, with lipoic acid acting in an antioxidant capacity by recycling Aldh2 to its active, reduced state.

### Leucine amino peptidase 3

Lap3 has attracted interest for its cysteine protease activity and its involvement in autophagy [54]–[56]. It was observed by Kagayama, et al., that Lap3 is specifically targeted to autophagosomes and is degraded under conditions of nitrogen starvation [54]. This suggests that at 96 hours postinfection, the infected monocytes might have entered nitrogen starvation, causing Lap3 to locate to the phagosome [79]. However, there is another possibility, as diagrammed in figure S3, and described below.

Activated monocytes have been shown to produce homocysteine [86]–[88], a byproduct of one-carbon methyl group donation from methionine [88], [89]. Homocysteine production appears to be at least partially regulated by the NF-κB pathway, most likely via modulation of cytokine levels [88]. Activation of the Toll-like receptors is known to trigger NF-κB activation, which further activates the cellular response to infection, including ROS activation [90]. Homocysteine is toxic to cells in high concentrations, possibly through conversion to homocysteine thiolactone, which can occur under cellular conditions [91].

Homocysteine thiolactone formation may be detrimental since: 1) Homocysteine thiolactone formation requires ATP, that would tend to dissipate energy reserves, 2) thiolactone binds to the epsilon-amino group of lysines forming damaged proteins that, 3) require additionial ATP/cellular energy resources to degrade via the unfolded protein response [92]. It has been observed that Lap3 has a protective role under conditions of elevated homocysteine levels, hydrolyzing homocysteine thiolactones [88]. Homocysteine toxicity is incompletely understood, but homocysteine has been observed to cause connective tissue and vascular damage [91]. We propose that elevated Lap3 levels in the *C. burnetii* infected monocytes are a cellular response to elevated ROS and may be accompanied by increased homocysteine production in activated monocytes in response to infection, as diagrammed in figure S3.

### Transaldolase

Transaldolase has been shown to contribute to regulation of the Pentose Phosphate Pathway (PPP), via recycling of glucose-6-phosphate (G6P) [93], in conjunction with transketolase. Transketolase has also been found to regulate the mitochondrial transmembrane potential (Δγ_m_) [57], [93]. Upregulation of transaldolase has been shown to increase the Δγ_m_, which precedes induction of apoptosis [94]. Increased levels of transaldolase have been shown to inhibit production of NADPH and GSH [94]. G6P was found to be significantly depleted in cells overexpressing transaldolase [57], [95]. G6P is the major input into the oxidative portion of the PPP, which is responsible for generation of NADPH [96]. At elevated ROS levels, the level of available NADPH reducing capacity in the cell to maintain GSH in its reduced state is depleted, thus favoring the increase in ROS-induced apoptosis in cells expressing higher levels of transaldolase, as diagrammed in figure S3. Apoptosis of infected host cells is a strategy for resistance of the organism to obligate intracellular pathogens. We propose that the increase in transaldolase expression that we observed is a cellular response to infection, that would normally lead to apoptosis were *C. burnetii* not acting to prevent this, as reported by Voth and Heintzen [64].

### Pyrophosphatase 1 (PPA1)

We observed that host PPA1 was upregulated in *C. burnetii* infected monocytes, most likely due to monocyte activation and maturation, as these conditions would be expected to increase the cells' metabolic needs, as diagrammed in figure S3. This does not rule out that at least part of the change in PPA1 is being regulated by *C. burnetii*. It was recently reported that pyrophosphatase 1 (PPA1) is upregulated in activated CD8^+^ T-lymphocytes [97], suggesting a function for PPA1 in immune responses. Bacterially derived PPA1 is essential for survival of *E. coli* and *Legionella pneumophila*. In *L. pneumophila* infections, the bacterium upregulates PPA1 in response to the phagosomal microenvironment of monocytes [98], [99], and *C. burnetii* may also be generating its own PPA1, below the limits of detection in the present experiments. The environmental activation of PPA1 observed in *L. pneumophila* may be a characteristic of γ-proteobacterial pathogens utilizing a dot/icm Type IV secretion system [98], [100], which is potentially of interest, since *C. burnetii* possesses a dot/icm system similar to *L. pneumophila*'s [100].

### Heat shock protein 60 (Hsp60)

We found that Hsp60 is upregulated in response to *C. burnetii* infection. Hsp60 can insert into the membrane as part of the antigen presentation machinery of macrophages, causing the stimulus of a Th1 type T-cell response in infected samples [52], [101]–[104], as diagrammed in figure S3. It has been proposed that Hsp60 is secreted from monocytes to act as a danger signal to attract and activate other immune cells [110]. In addition, exposure to Hsp60 seems to induce attenuated responses to later inflammatory stimuli, including LPS and additional Hsp60 [105]. It has been proposed that the receptors TLR2, TLR4, and the coreceptor CD14 are involved in Hsp60 recognition and/or binding, although Hsp60 responses independent of these receptors have also been proposed [52], [101]–[104]. It has been noted that there is an increase in CD4^+^CD25^+^ inhibitory T-cell activity in the presence of Hsp60, suggesting a role of Hsp60 in regulating the adaptive immune response [104].

It is possible that some of the upregulated Hsp60 is being secreted to attract and/or regulate nearby immune cells. As described above, innate immune cells have cell surface receptors for Hsp60, but these are not completely described. It has been shown that increased Hsp60 exposure increases activation of p38, NF-κB, and Erk, but not Jnk [102], [106], [107] (see figure S3). *C. burnetii* infection causes an increase in Akt and Erk1/2 activation, but a decrease in p38 activation [64]. Another possible Hsp60 function may be stimulating the down regulation of ECH (discussed above and diagrammed in figure S3). Hsp60 can be secreted, and Hsp60 seems to be able to interact with the toll-like receptors (TLRs) [52], [102]–[105], [107]–[110]. Hsp60 could thus be regulating TLR-based immune responses, including the downregulation of ECH. It is also possible that the Hsp60, particularly that observed in the membrane fraction of *C. burnetii* infected cells, is involved in antigen presentation to T-cells, as reported in the literature [52], [101]–[104]. Thus Hsp60 could be providing a link between the monocyte response to infection and activation of a T cell-mediated adaptive immune response, although additional experiments would be required to test this hypothesis.

### Rab7

As diagrammed in figures S3 and 4, elevated Rab7 has a number of potential implications for *C. burnetii* infected monocytes. The elevation of Rab7 GTP binding protein signals entry into the phagolysosomal maturation process and exit from the early/recycling endosomal stage, including acidification to a final pH of ∼4.5 in a fully mature phagolysosome [111]. *C. burnetii* is an acidophile, requiring an acidic environment for survival [65], [112], [113]. *C. burnetii* requires a phagolysosomal pH of ∼4.5–5 in order to replicate, and raising the pH above ∼6 will prevent replication [65], [113]. Rab7GTP has been reported to mediate the acquisition of vacuolar ATPase (vATPase), required for acidification of the maturing phagolysosome [114]. Bucci et al., reported that phagosomes overexpressing a DN-Rab7 construct (that is locked in the inactive GDP conformation), showed reduced phagolysosome acidification [114]. Maintaining functional vATPase at the phagolysosomal membrane, appears to be part of the bacterium's survival strategy. It has been reported that the active form, Rab7GTP increases the size of phagolysosomes, as reasoned from effects of the constitutively active Rab7GTP analog Rab7Q67L [114]. Phagosome maturation is normally accompanied by hydrolase delivery to the phagosome [79]. Alteration of Rab7's recruitment by *C. burnetii* may require bacterial protein synthesis, since exposure of *C. burnetii* infected cells to chloramphenicol (a bacterial protein synthesis inhibitor) prevented maturation of *C. burnetii* infected phagolysosomes into replicative vacuoles [9].

It has been reported that lipid rafts or lipid raft-like domains are present in monocytes not only at the plasma membrane, but also in internal membranes, including the ER and the phagosome [115]. Rab7 may be lipid raft associated and increased cholesterol accumulation has been shown to increase Rab7 recruitment to the phagolysosome [71]. Li et al., reported that monocytes localize both Rab7 and vATPase in lipid rafts [115]. Rab7 binding to the phagolysosomal membrane has been shown to be sensitive to the cholesterol composition of the phagosome membrane, supporting a lipid raft localization of Rab7 [71]. Increasing the cholesterol content of the phagosomal membrane is correlated with an increase in Rab7 recruitment [71]. Inhibiting Rab7 recruitment would impair maturation the development of the phagolysosome [114], [116]–[119]. These observations suggest that inhibition of Rab7 in *C. burnetii* infected phagolysosomes/replicative vacuoles might be an effective form of treatment to slow the progression of the infection [10], [120]. We suggest that *C. burnetii*, by altering the monocyte phagolysosomal lipid raft composition, not only increases Rab7GTP and vATPase recruitment, but would also interfere with hydrolase delivery [13], as diagrammed in figure S3.

It was shown by Howe et al., [12] that both phase I and phase II *C. burnetii* replicative vacuoles contain active cathepsin D, which would seem to contradict the findings of Ghigo et al., [13]. It is important to note that Ghigo et al., [13] looked at primary monocytes in humans recovering from acute Q fever that had either progressed to chronic Q fever-induced endocarditis or had been cured of endocarditis. Howe et al., [12] looked at a much shorter time frame, consistent with the acute phase of infection. The differences in the timing of the experiments may explain the differences in the observations of the two groups. Regulation of lipid raft composition to regulate the delivery of hydrolases may therefore be of greater importance in more advanced Q fever infections.

*C. burnetii* infection has been shown to alter gene expression of key enzymes in the cholesterol biosynthesis pathway [10]. Furthermore, inhibition of cholesterol synthesis by statins inhibits *C. burnetii* replication [10] and *C. burnetii* infected cells produce up to 73% more cholesterol than uninfected cells [120], suggesting that *C. burnetii* might be directing an increase in cholesterol production rather than simply causing a redistribution of cholesterol. These observations led Voth and Heinzen to suggest that *C. burnetii* replicative vacuoles experience an increase in both flotillin protein (a lipid raft marker) and Rab7 expression [120], in agreement with the results of Li et al., [115]. Voth and Heinzen further suggest that an increase in phagolysosomal cholesterol content may be responsible for the observed resistance of *C. burnetii* infected phagolysosomes to mechanical disruption, as well as the decreased ionic permeability, thus facilitating the maintenance of the low phagolysosome pH necessary for *C. burnetii*'s life cycle [120]. As pointed out previously, raising the phagolysosome to a pH of ∼6 was shown to reduce *C. burnetii* viability [65], [113]. *C. burnetii*'s dot/icm secretion systems may be involved in regulating Rab5 and Rab7 proteins, potentially providing a further layer of regulation of phagosome maturation by the bacterium.

Information from the literature indicates that *C. burnetii* inhabits an autophagolysosome composed of autophagic vacuoles fused with a mature phagolysosome [11]. Rab7 is involved in the development of autophagic vacuoles in a manner analogous to phagosome maturation [117]. Autophagic vacuole fusion would deliver necessary nutrients. *C. burnetii* appears to be genetically unable to produce 11 amino acids (either missing entire pathways or key enzymes thereof), but does contain a number of amino acid transporters [2]. Proteins and peptides delivered to the phagolysosome would be broken down to amino acids for uptake by *C. burnetii*, and delivery of the essential amino acids via autophagosomes is presumably required for *C. burnetii* survival. *C. burnetii* is known to produce greatly enlarged phagolysosomes [120] and autophagosomal fusion with the maturing phagolysosome suggests a mechanism by which *C. burnetii* replicative vacuoles are able to grow to such large sizes.

It seems clear from the literature that one survival strategy for intracellular pathogens is to alter the time course of Rab protein expression. *Leishmania donovani* has been shown to inhibit phagosome acquisition of Rab7 [118], and a similar strategy seems to be employed by *Salmonella typhimurium* [121]. Our model predicts that maintaining Rab7 recruitment to the phagolysosome is important for *C. burnetii* maturation and survival but the mechanism used by *C. burnetii* to maintain Rab7 recruitment has not yet been determined. An attractive hypothesis is that *C. burnetii* is regulating the composition of lipid rafts via upregulation of host cell cholesterol synthesis. The analysis of the phagosomal proteome by Garin et al., (2001) [79] suggests that there are a number of protein targets, in addition to the lipid raft composition, that are affected by *C. burnetii*. Given that statins have been shown to inhibit *C. burnetii* infection [10], [120], it would be interesting to monitor the response of *C. burnetii* infected monocytes to statins +/− immune adjuvants [14].

## Conclusions

The model developed in this study hypothesizes that upregulation of S100A8/9 in *C. burnetii* infected monocytes as well as vimentin indicates that the monocytes have begun to differentiate, and that the microbicidal properties of the S100A8/9 heterodimer are active. We further hypothesize that secretion of S100A8/9 may be regulated by the activation status of monocyte GABA_A_ receptors. Evidence has been presented that inhibition of the GABA_A_ receptor by Securinine, a GABA_A_ receptor antagonist [122], increases monocytic cell killing of *C. burnetii* [14]. GABA_A_ receptors are present in monocytes, and GABA_A_ receptors are known to be susceptible to downregulation upon activation of PKC [14], [73]–[75], [123]. We provide evidence in a companion paper that Securinine stimulation of monocytes initiates an innate immune response that is distinct from the TLR-mediated response [126]. S100A8/9 are believed to be early acting [72], and more mature macrophages may not display a significant upregulation of these proteins, consistent with the proposition that monocytes in the *C. burnetii* infected samples at 96 hours are at an early stage of maturation.

The observation that Hsp60 could be involved in many facets of innate immune responses has recently begun to be appreciated [52], [101], [124]. It is distinctly possible that increased expression of Hsp60 and export to the secretome is responsible for many of the proteome changes directed by membrane receptors, including the downregulation of enoyl CoA hydratase, and could have a role in changing the redox status of the cell (e.g. via regulation of aldehyde dehydrogenase and transaldolase). The upregulation of visfatin would provide more NAD for driving redox reactions. The regulation of various central metabolic proteins after *C. burnetii* infection suggests that a more comprehensive understanding of changes in the metabolome and lipidome may provide a deeper understanding of the monocyte response to infection. Particularly, changes in the levels of various metabolites and lipids can give an indication of the flux of these compounds through the cell during infection. This in turn could be used to infer the activation status of various metabolic enzymes that could be changing their activity without changing their expression levels, e.g. by sensing the ratios of ADP/ATP or NAD^+^/NADH.

While the overall alterations to the proteome suggest an innate immune response is being activated by *C. burnetii* infection, there are also points of interest related to *C. burnetii* biology. The elevation of S100A8/9 as well as vimentin suggests the onset of macrophage differentiation as pointed out above. The elevation of apparently intact vimentin suggests that caspases are not highly active in the infected cells, consistent with observations that *C. burnetii* can inhibit caspase activation [64]. The changes in the two Rab7 isoforms in our experiments, as well as observations that Rab7 is a lipid raft associated protein, warrants further scrutiny. Rab7 would be predicted to be critical to *C. burnetii*'s metabolic and replicative activities as detailed above. Disruption of cellular lipid raft remodeling by disruption of cholesterol synthesis with statins is expected to have a synergistic effect with antibiotic and/or immune adjuvant treatment protocols. Therefore, altering the composition of lipid rafts might be an attractive mechanism for *C. burnetii* to modulate Rab7 and vATPase localization.

The recent work by Howe et al., [12] demonstrated that phase I and phase II *C. burnetii* induced phenotypically indistinguishable responses from both primary and cultured monocytes at least with regards to the replicative vacuole. These findings support the use of phase II *C. burnetii* in cultured monocytes as a model system for studying *C. burnetii*-host cell interactions. It is also of note that Vero cells, a Green monkey kidney epithelial cell, commonly used in *C. burnetii* investigations, tends to give similar results to those obtained in monocytic cells [12]. This suggests that *C. burnetii* infections have similar effects on a variety of different cell types and suggests further that the system used in our investigation can be extended to pathogenic conditions in primary cells. The observation of *C. burnetii*'s effects on NADPH oxidase assembly, coupled with the observation that lipid rafts regulate the activation state of NADPH oxidase in renal cells [125], further supports the hypothesis that *C. burnetii* infections (phase I and II) are able to regulate the lipid raft composition of the host cell. The finding that cholesterol depletion increased NADPH oxidase activity in renal cells [125], supports the hypothesis that inhibiting cellular cholesterol production (e.g. via statin administration) in combination with other treatments could improve therapeutic approaches to Q fever infection.

The changes in protein expression described above, as well as the proposed functions of the regulated proteins in this system would be predicted to be similar between phase I *C. burnetii* and phase II, as well as between Monomac I cells and primary monocytes. This prediction is based in part on the work of Howe et al, [12], but also on the numerous publications cited above in multiple different innate immune systems [46], [49], [52], [68]–[70], [72], [77], [97], [101]–[103], [107], [112], [113]. This suggests that the use of phase II *C. burnetii* and Monomac I cells provides a useful basis for formulating models and guiding experiments that can be extended to the select agent (phase I *C. burnetii*) in primary cells. Statin treatment protocols, including cotreatment with e.g. antibiotics, or immune adjuvants, for Q fever, if successful, could potentially be extended to other obligate intracellular pathogens and possibly to the treatment of the diseases caused by these pathogens. Our goal is to use this systems model to integrate findings and guide future investigations.

## Supporting Information

Figure S1 **Flowchart of methodology.** This flowchart briefly describes the workflow used during this investigation.
(TIF)

Figure S2 **Soluble protein fraction 48 hr postinfection.** Progenesis master image following gel image analysis and offline statistical analysis. The analysis was for 3 biological replicates with 6 technical replicates for biological replicates 1 and 2 and 5 technical replicates for biological replicate 3. Technical replicates included reciprocal Zdye color labeling as described in the text. Spot 10 was determined to be differentially expressed in a statistically significantly manner. See tables 1 and 2 for identification.
(PDF)

Figure S3 **Systems biology network model proposed for the response of Monomac I cells to infection by** ***C. burnetii.*** This model was developed by text mining the literature on the proteins regulated by *C. burnetti* infection. A red box around a node indicates that the protein was up regulated in *C. burnetii* infected monocytes vs. unstimulated controls and a green box indicates that the protein was down regulated. The colored edges present in the image are for clarity only, and imply no special significance to the nodes they are connected to.
(PDF)

Table S1 **Isoelectric focusing profiles used in this investigation.** All strips were loaded using active rehydration as described in the text and methods S1.
(XLS)

Table S2 **Syntax and data structure for SPSS data analysis of the protein spot intensity changes on 2D gels.** Syntax for nested ANOVA (mixed linear model) for use in SPSS v. 16. This program directs the SPSS software to generate an array and then group data appropriately. Technical replicates are grouped within their appropriate biological replicate and the samples are also grouped by treatment condition. SPSS then performs a two-way ANOVA to generate the p-values (<0.05) and observed power scores (>0.7) reported in this study.
(XLSX)

Data S1 **Log normalized spot volumes derived from Progenesis analysis of the 2D gel spot data.** The data are organized as follows: Each column represents a single biological replicate with up to six technical replicates per biological replicate. Control indicates the control sample uninfected monocytes, infect indicates data derived from the infected samples. These numbers were input into SPSS for further analysis using the nested ANOVA approach. An indication of pass at the bottom of each set of data indicates that the spot passed analysis and was picked for MS identification. An indication of fail at the bottom of the data indicates the spot did not meet the statistical cutoffs and was not selected for further analysis. For reference, the cutoffs for the nested ANOVA analysis were p<0.05, and power score >0.7. A spot meeting both of these criteria was selected for further analysis.
(PDF)

Data S2 **Sequence coverage maps for identified proteins.** Red indicates sequence coverage. The underscores seen in certain amino acids indicate possible sites of posttranslational modification. Data is presented for all bioinformatics tools used.
(PDF)

Methods S1 **Additional methodology information for this investigation.** Additional information on the methods used in this investigation. References cited are listed in the references section of the manuscript.
(DOC)
