# Supplementary figures and images for: Proteomic and Systems Biology Analysis of the Monocyte Response to Coxiella burnetii Infection
Source: PLoS One. 2013 Aug 21;8(8):e69558. doi: 10.1371/journal.pone.0069558 (PMC3749201; doi:10.1371/journal.pone.0069558)

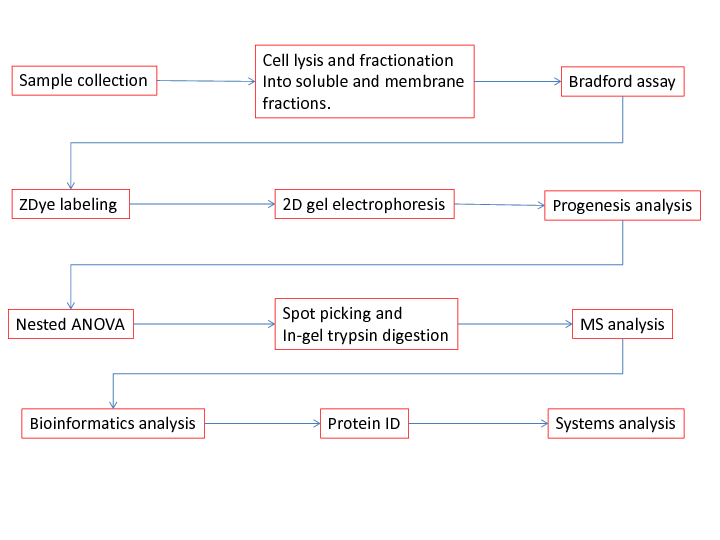

Supplement: Figure S1 — Flowchart of methodology. This flowchart briefly describes the workflow used during this investigation. (TIF) [file pone.0069558.s001.tif]

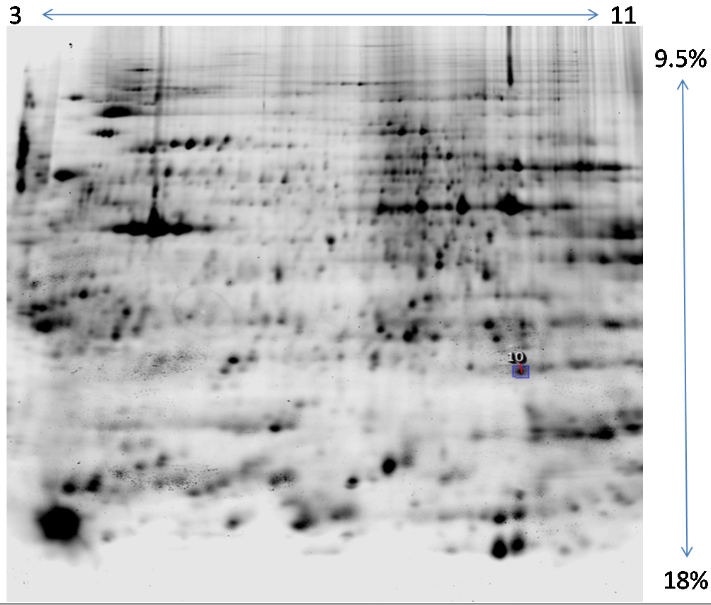

Supplement: Figure S2 — Soluble protein fraction 48 hr postinfection. Progenesis master image following gel image analysis and offline statistical analysis. The analysis was for 3 biological replicates with 6 technical replicates for biological replicates 1 and 2 and 5 technical replicates for biological replicate 3. Technical replicates included reciprocal Zdye color labeling as described in the text. Spot 10 was determined to be differentially expressed in a statistically significantly manner. See tables 1 and 2 for identification. (PDF) [file pone.0069558.s002.pdf]

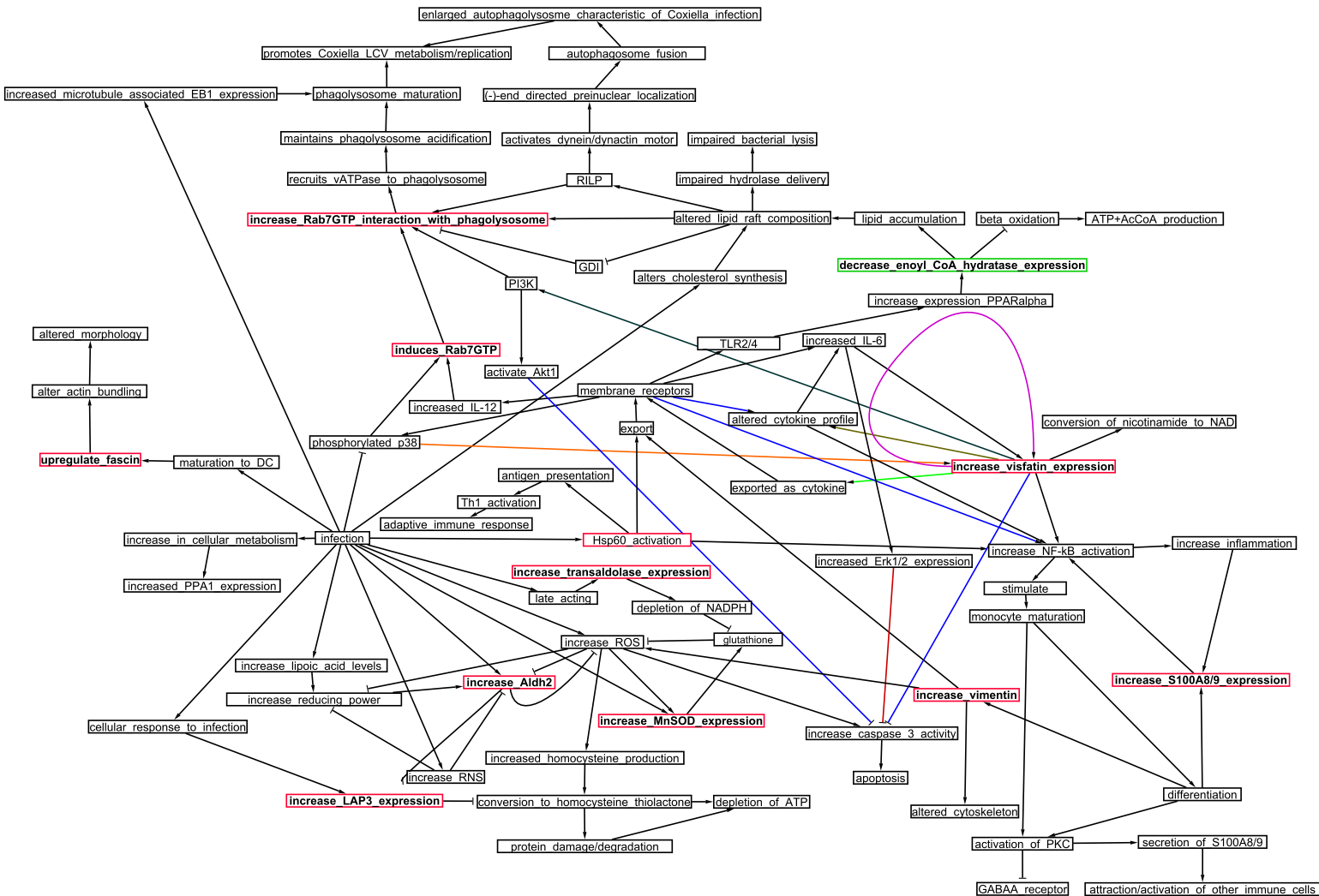

Supplement: Figure S3 — Systems biology network model proposed for the response of Monomac I cells to infection by C. burnetii. This model was developed by text mining the literature on the proteins regulated by C. burnetti infection. A red box around a node indicates that the protein was up regulated in C. burnetii infected monocytes vs. unstimulated controls and a green box indicates that the protein was down regulated. The colored edges present in the image are for clarity only, and imply no special significance to the nodes they are connected to. (PDF) [file pone.0069558.s003.pdf]
