# Supplementary material for: Proteomic and Systems Biology Analysis of the Monocyte Response to Coxiella burnetii Infection
Source: PLoS One. 2013 Aug 21;8(8):e69558. doi: 10.1371/journal.pone.0069558 (PMC3749201; doi:10.1371/journal.pone.0069558)
Supplement: Data S1 — Log normalized spot volumes derived from Progenesis analysis of the 2D gel spot data. The data are organized as follows: Each column represents a single biological replicate with up to six technical replicates per biological replicate. Control indicates the control sample uninfected monocytes, infect indicates data derived from the infected samples. These numbers were input into SPSS for further analysis using the nested ANOVA approach. An indication of pass at the bottom of each set of data indicates that the spot passed analysis and was picked for MS identification. An indication of fail at the bottom of the data indicates the spot did not meet the statistical cutoffs and was not selected for further analysis. For reference, the cutoffs for the nested ANOVA analysis were p<0.05, and power score >0.7. A spot meeting both of these criteria was selected for further analysis. (PDF) [file pone.0069558.s006.pdf]

48 HR SOLUBLE FRACTION:

Spot 2

Controlrep1	Controlrep2	Controlrep3	Infectrep1	Infectrep2	Infectrep3
90960.17	45210.5623	27885.8943	219950.34	60735.531	502831.28
62293.886	68760.0738	2958.59098	201415.24	67796.078	56778.977
42995.9756	56437.9214	35756.5372	107493.83	89449.082	6924.0426
30642.6021	52357.9815	37877.8352	163930.08	97159.246	28266.741
67031.6005	26526.7051	32538.4556	208101.96	52622.603	46080.159
36405.0286			272344.08		

Fail

Spot 5

Controlrep1	Controlrep2	Controlrep3	Infectrep1	Infectrep2	Infectrep3
81011.5719	115464.281	88316.421	164084.33	112818.6	567451.11
80996.5268	177536.366	101405.955	284117.77	148346.39	148337.68
69486.3172	51013.2678	55038.8951	149984.84	75773.859	41878.213
54464.889	32577.6496	22493.0432	118522.36	77018.213	52795.519
82229.0242	49366.3119	66098.4762	185119.83	104664.75	93476.417
208705.569			788345.54		

Fail

Spot 10

Controlrep1	Controlrep2	Controlrep3	Infectrep1	Infectrep2	Infectrep3
6576.81079	28597.9292	18068.9949	14993.155	26378.572	31171.444
14838.8384	17807.1854	10573.8503	47131.084	19150.804	15933.003
26349.939	12182.6788	10734.8891	24291.354	19267.403	12288.021
9065.47291	7661.77303	6359.65918	36378.874	10816.934	5589.7941
7583.78401	12414.0697	5768.55886	33428.206	18596.898	5868.48
15370.4164			51146.846		

Pass

Spot 12

Controlrep1	Controlrep2	Controlrep3	Infectrep1	Infectrep2	Infectrep3
662857.178	1165093.83	1066759.87	810030.23	2093247.4	1442119.6
921174.709	1077719.89	1572876.56	1286950.7	1694136.6	1692749.8
864864.541	688184.196	1281533.39	877216.81	1080341.5	2401889
517825.195	619471.75	949609.232	1235268.7	1879285	2202727.4

919987.089	651046.211	1109778.07	1687304.8	1011270.8	1566448.3
794241.616			1834762.7		

Fail

Spot 13

Controlrep1	Controlrep2	Controlrep3	Infectrep1	Infectrep2	Infectrep3
116570.706	237879.193	54436.6512	261328.43	440401.75	98939.281
201399.168	110602.665	74734.3193	365179.36	285237.23	116819.68
192690.262	100624.597	152111.755	416982.27	180634.18	152042.13
102465.474	99634.8453	415378.673	170945.35	220209.64	415889.03
215942.988	98743.3254	102757.12	308441.56	147601.75	71791.078
112487.267			230897.6		

Fail

48 HR MEMBRANE

Spot 1

Controlrep1	Controlrep2	Controlrep3	Infectrep1	Infectrep2	Infectrep3
343331.3906	330716.4787	379484.6312	337298.8013	729831.6555	784507.4295
201231.1758	282761.6389	448433.0792	158047.6898	664186.8441	971141.6572
201374.5049	422468.3394	421474.3519	181244.4113	955298.6262	808364.6034
307937.042	309675.6615	350583.2891	296286.85	744980.2	678968.8268
140862.6366	217293.5187	377819.5537	148745.7873	545447.188	705093.9113
264083.3381	356461.2205	360614.1704	225870.0291	720743.4889	712265.1622

Fail

Spot 2

Controlrep1	Controlrep2	Controlrep3	Infectrep1	Infectrep2	Infectrep3
537325.0692	680737.0004	906289.4929	1500952.261	765705.4442	869816.2291
566459.479	900269.3397	1300806.219	1375603.664	938347.0805	1099754.559
256714.1389	936408.5515	1361420.378	995456.055	935252.4681	1267904.944

602877.0195	567451.416	761728.6333	735694.4316	690244.9548	812580.7701
420588.7304	913976.6275	1597487.897	717282.5408	1318655.862	2076473.543
514852.9416	698696.6536	921891.5168	615550.2069	855117.3728	2027438.093

Fail

96 HR SOLUBLE

Spot 2

Controlrep1	Controlrep2	Controlrep3	Infectrep1	Infectrep2	Infectrep3
611587.5259	325124.4428	565481.264	2031615.209	1221698.812	1866484.055
276312.6549	232718.8176	543136.8167	851526.4511	796546.0302	2388767.574
334817.9191	384021.4955	527127.4206	1116637.904	1277799.604	2391435.995
305594.5708	268861.0828	397614.8593	1682843.337	1112198.365	817849.0592
728119.0239	315524.5588	404965.254	4198160.951	1545471.642	1292233.404
255306.6622	258472.8291	317984.5377	926010.9715	1551942.983	1601058.204

Pass

Spot 5

Controlrep1	Controlrep2	Controlrep3	Infectrep1	Infectrep2	Infectrep3
280304.9268	306197.5465	625318.8236	1092222.052	1764853.205	1275113.411
471402.0895	727856.2743	543046.0899	1244075.476	2187108.286	1264469.058
239830.652	346349.1804	308129.2279	919450.6142	1729786.328	962406.5609
398354.9362	433413.0539	511103.5062	829729.3719	1576322.486	1255515.614
346710.6423	298274.338	438511.1937	763778.4151	1481004.226	983159.7285
483555.0759	167414.7918	568954.2984	722303.6965	882026.3423	1418783.963

Pass

Spot 9

Controlrep1	Controlrep2	Controlrep3	Infectrep1	Infectrep2	Infectrep3
231363.8488	195044.208	389821.7999	763045.0016	601725.6212	1018482.706
127809.498	198864.2092	411709.4589	303927.1427	432038.1901	1038211.101

211916.3871	149708.7999	499386.1619	462571.2876	580839.9743	1217546.956
133679.8059	119820.1158	264652.6052	560828.6184	597778.3135	575126.2479
278544.7697	101334.948	153301.4982	745275.3778	411023.9895	194386.9659
183947.153	140823.2087	110110.6874	401838.7109	396653.228	236568.3484

Pass

Spot 12

Controlrep1	Controlrep2	Controlrep3	Infectrep1	Infectrep2	Infectrep3
30973.58017	36720.25651	142113.296	37117.03441	232748.8545	386213.8193
7587.313338	56562.5869	95755.94145	22114.98755	314733.4095	258857.1562
25791.48407	89360.77424	135215.8198	78722.04499	415794.8276	393410.7434
2527.140132	96371.8224	160080.344	47935.4774	225297.8748	257755.6015
9480.098452	93141.5575	256067.2268	42795.65269	279372.0138	358223.8196
7467.852439	81757.72798	144098.3144	56888.6829	273321.5111	229443.2138

Fail

Spot 13

Controlrep1	Controlrep2	Controlrep3	Infectrep1	Infectrep2	Infectrep3
68929.11847	93123.38785	1465237.997	337412.6856	732965.6947	1288738.418
107220.5271	74248.30387	58718.38533	370657.8796	800644.533	597147.4681
101103.0762	116874.898	70475.83957	382906.195	592214.0398	544641.8596
156327.9221	187990.1488	115369.9268	331178.2498	432559.1144	246887.0355
106196.4947	61163.53265	74416.82734	92516.9016	474941.8703	249813.3961
127935.9991	78861.74867	106541.7577	229801.0207	352513.8523	305860.8074

Fail

Spot 14 Pass

Controlrep1	Controlrep2	Controlrep3	Infectrep1	Infectrep2	Infectrep3
359427.4097	519382.9257	715881.1561	837921.6701	539688.468	3757318.129
328378.398	431108.7407	435385.8095	1117138.372	420249.6378	2103993.316
568479.2482	535838.4745	171939.8292	2187248.765	510582.705	503818.9664
295811.6544	353199.5429	246427.0851	493641.683	930239.5147	370221.0528
394888.5056	508429.0288	126475.1797	819511.2896	1201013.508	328462.7044
359309.224	350266.3407	253794.8384	857819.2399	778358.5687	389585.3349

Spot 16

Controlrep1	Controlrep2	Controlrep3	Infectrep1	Infectrep2	Infectrep3

21215.95076	73583.36245	143238.8257	61657.97491	224926.4368	282275.5982
36378.69148	90704.3798	139095.749	76553.04699	406378.4796	332885.3814
64634.45658	117627.4978	186798.8223	98220.29324	532380.9481	453675.7919
19663.03494	155466.5063	153975.2904	53054.31935	330370.3297	149273.1018
35489.07153	114865.6303	217466.2725	55223.95131	396644.166	420284.4545
37338.16149	156035.5671	198856.7292	60351.02041	417411.8391	350036.8861

Fail

Spot 17

Controlrep1	Controlrep2	Controlrep3	Infectrep1	Infectrep2	Infectrep3
47225.8805	54871.63743	27431.87682	17223.69313	11512.52628	9616.25173
31284.22783	35026.50747	6713.396722	6861.2047	7816.429197	7667.590238
38671.76328	38944.68588	19706.97876	17037.77238	17839.08011	6246.898165
23229.2362	76382.6474	20206.68994	18962.27284	12876.40463	4891.875418
49315.08644	110820.6819	21915.10313	38019.38466	25239.39456	5556.062085
79144.68142	116994.6819	41107.53313	58280.66609	71687.43478	14778.71087

Fail

Spot 18

Controlrep1	Controlrep2	Controlrep3	Infectrep1	Infectrep2	Infectrep3
660996.3351	455483.7887	297018.4608	243060.9163	167872.4883	116727.0299
322184.3806	649233.9622	219257.0602	134073.2502	182924.6404	81178.0794
657290.2594	582304.2454	305794.9928	279998.3772	244235.7902	123783.23
510091.7389	1025382.367	308705.4761	288939.6574	392719.1152	141578.6133
528665.885	1422866.974	305455.0888	302796.4139	446665.4124	142512.6806
943314.2843	550143.8698	371348.219	615187.3882	180558.0139	177083.1354

Fail

Spot 29

Controlrep1	Controlrep2	Controlrep3	Infectrep1	Infectrep2	Infectrep3
917077.124	634434.9147	386197.2816	324754.132	117003.061	191266.4775

452953.8869	739307.9531	109583.679	96390.42757	124314.1834	131379.9935
839985.6226	667768.9895	187687.3843	282406.3664	131003.3792	92698.27989
426473.4455	535025.2297	505247.2057	164899.188	425573.1175	404027.5523
505808.8649	734806.5811	242409.6066	443455.8208	465897.504	239410.7945
339111.0593	407253.6125	397255.0276	145772.6963	288064.3143	324578.1084

Fail

Spot 46

Controlrep1	Controlrep2	Controlrep3	Infectrep1	Infectrep2	Infectrep3
295546.3931	87741.62322	65304.63189	469914.2528	304957.773	132962.8075
258173.4581	100243.4567	167237.5318	397932.2162	299316.0561	361096.0101
326359.5137	113101.4293	150904.7393	535423.6894	361014.0047	332815.8459
339623.0091	93270.13331	264354.4133	459821.0945	226383.7382	324320.2505
327356.2853	86478.01424	167474.7624	419594.8272	233575.5983	213141.1626
367033.6333	218512.8722	188984.4162	462963.5557	360295.7955	278896.6716

Fail

Spot 48

Controlrep1	Controlrep2	Controlrep3	Infectrep1	Infectrep2	Infectrep3
232531.4386	244001.5224	252086.6153	352317.0249	322549.2956	244441.8207
105250.2786	194467.3878	180660.9905	176398.6001	340127.0261	191371.9467
159286.0062	175300.7773	189811.3436	327660.3016	252757.1755	240648.6461
212564.3283	267093.7667	182504.3993	502689.0967	552895.7363	312530.5377
377039.9437	303084.3927	144281.6656	514285.5109	704963.9129	219262.4168
144607.3976	112729.4853	261940.8347	328351.8892	253256.3219	474989.6442

Pass

Spot 50 Fail

Controlrep1	Controlrep2	Controlrep3	Infectrep1	Infectrep2	Infectrep3
244422.899	207378.8433	171752.7998	250199.9783	101431.6955	52471.31418
181125.7068	337914.2425	107064.9796	134371.9957	198650.1111	105957.4282
144313.6442	268003.5341	211085.8377	140040.4197	143510.5997	88539.44645
230034.7093	341345.1943	246283.7965	158035.8527	115574.0893	149038.8538
196696.6501	489477.0065	288203.1121	172879.3605	196307.0814	173914.5861
309235.7205	222211.6791	233491.9812	208612.6781	81495.40933	158661.5066

Spot 53

Controlrep1	Controlrep2	Controlrep3	Infectrep1	Infectrep2	Infectrep3
228377.2914	460043.5576	355875.1045	133611.5869	307628.3447	256845.3051
202550.7436	447388.9422	271340.55	123389.3261	308923.5105	241359.4824
306171.063	395664.5243	244972.3498	153570.0456	218321.8957	177335.904
292452.2193	408974.0565	377962.3855	115986.3097	256854.6208	214601.7021
250308.512	706418.4175	241340.9129	86898.61866	460221.8118	143000.676
360746.747	540598.8001	370783.541	124318.101	342883.8887	208304.2038

Fail

Spot 60

Controlrep1	Controlrep2	Controlrep3	Infectrep1	Infectrep2	Infectrep3
497488.4839	898231.7594	479057.1789	746357.5002	1337990.526	682065.9166
254908.1887	361674.7365	550667.7836	665694.3124	509387.1535	830393.6892
332250.8142	387799.5591	867675.2809	801651.1243	624754.2073	1256457.442
390922.7869	411585.5497	378720.2559	869251.9034	658851.7487	432966.7266
415495.905	406247.4659	807772.0087	1001630.476	577987.1857	1103357.657
462715.0944	554712.6494	706957.9279	917559.5739	855233.2556	1026146.241

Pass

Spot 63

Controlrep1	Controlrep2	Controlrep3	Infectrep1	Infectrep2	Infectrep3
368583.5605	590553.0568	376065.9782	504331.4537	814838.513	516646.5943
331198.3507	547259.4716	434356.7898	424902.5456	708600.5259	586398.4135
400347.4194	420467.1643	438408.5	553063.5431	605096.5943	632820.5643
270522.2235	298718.8332	472611.0186	623356.8755	647671.997	874899.0137
548903.4876	312482.0516	166759.7949	711104.2466	678126.4599	298992.2875
304821.8032	386187.7975	453551.6255	651816.1072	836857.1624	877630.2038

Pass

Spot 69

Controlrep1	Controlrep2	Controlrep3	Infectrep1	Infectrep2	Infectrep3

126923.4235	98821.57291	89526.52428	172576.0851	150043.3366	44582.62557
106081.2456	100593.8566	193087.8041	176074.5846	146732.8481	144059.9888
99685.67516	233644.9522	267397.009	268397.6099	328577.9058	233774.6484
111253.4275	132500.5701	141890.9927	238459.8232	276838.5652	236436.1219
137858.9615	139461.3781	199277.2902	257934.2863	331041.2643	312392.8087
137158.1257	143281.1506	225815.3526	251494.694	343881.0463	386831.9632

Pass

Spot 70					
Controlrep1	Controlrep2	Controlrep3	Infectrep1	Infectrep2	Infectrep3
42402.1011	72169.78587	150339.1302	115148.3087	112274.9931	185690.1228
20158.70542	69059.5573	119074.7761	83570.12446	133112.3127	174924.4656
40000.12962	75551.27081	115305.2881	91712.26129	165501.7587	133291.9078
66018.29888	149799.5222	141121.4179	159443.1047	181108.073	225963.4725
139896.0644	189181.1813	89467.26786	148519.4074	318364.5523	138775.9771
89496.89516	50371.8461	131101.1778	101394.6247	129992.6545	202570.1733

Fail

Spot 82					
Controlrep1	Controlrep2	Controlrep3	Infectrep1	Infectrep2	Infectrep3
123090.6225	117647.4198	95928.50803	203647.3209	204986.1464	162281.9486
107308.3029	77009.4223	84264.6886	163449.1007	143153.6929	129004.2159
129468.681	112312.9762	55653.71433	194299.9262	213119.6832	88393.70909
82300.62	39461.63677	63384.04595	141986.5658	67421.23338	92746.26646
92191.97823	75396.36869	27895.61745	140585.6931	131337.4423	37492.97278
214933.0749	57772.96633	55712.95785	219640.1596	86451.38761	84137.72875

Fail

Spot 85	Fail				
Controlrep1	Controlrep2	Controlrep3	Infectrep1	Infectrep2	Infectrep3
57242.25933	81760.14165	160007.5836	62538.43864	47767.08163	104162.417
37279.41184	77321.47442	86109.78054	47586.69114	51924.22665	77256.85302
44879.69504	68477.4297	52998.88403	43247.89979	38153.09972	36923.36696
50007.27037	177029.5367	65258.2808	24697.56624	87669.95892	41786.73291
62485.61286	87378.85377	105612.0766	33118.48123	49801.99402	65055.79893
44495.05721	90184.36997	121126.9017	20910.71581	41401.41739	79963.95238

Spot 87

Controlrep1	Controlrep2	Controlrep3	Infectrep1	Infectrep2	Infectrep3
381002.3997	251022.2891	213154.8927	742866.1035	501496.5119	333794.7151
300344.8284	206057.0227	277617.3643	611973.1649	428642.7762	525784.1407
233763.1228	293547.2908	195909.4985	516690.0087	528870.144	404701.7314
555591.1924	347238.9633	517975.6665	650355.5752	492228.0057	695001.075
468631.7496	282094.0723	307357.2148	497376.3459	481027.7357	336067.5678
303847.6066	220483.3118	371541.3682	278718.6182	345593.058	428657.058

Pass

Spot 88

Controlrep1	Controlrep2	Controlrep3	Infectrep1	Infectrep2	Infectrep3
152769.9791	49420.34612	55630.03462	252961.0962	145832.6865	92608.67278
112800.7611	58587.80548	103595.8356	168340.1255	115228.444	201922.5887
199735.647	66406.76752	70382.68656	300274.0444	154265.1649	131540.7816
156642.5506	63148.77366	139018.6461	219418.6251	90638.67394	164577.1796
109170.1045	83588.6333	114962.7754	145876.1361	138597.0978	130570.1898
182501.6618	135176.1201	110842.0389	221050.1276	176529.0094	148723.5029

Fail

Spot 94

Controlrep1	Controlrep2	Controlrep3	Infectrep1	Infectrep2	Infectrep3
267763.9506	172539.2211	120828.1892	102697.0494	117383.8904	74740.63436
209181.8431	181675.8911	138646.779	98383.36593	97269.55352	102870.4581
215905.8899	142277.2018	92285.36517	101075.923	119009.5554	108272.8927
318949.7704	178526.5817	169730.6985	137305.3194	159183.4308	142499.2337
386879.2313	172418.1746	153565.335	201499.4225	152714.475	163893.7698
368036.6311	154997.2451	179222.0216	178499.1635	166921.7905	187314.3073

Fail

Spot 98

Controlrep1	Controlrep2	Controlrep3	Infectrep1	Infectrep2	Infectrep3
122365.6184	187120.2598	104005.4027	246211.6368	387920.7352	189274.4077
123155.9562	196554.9232	234542.4112	226865.7295	393490.0536	338300.2895
72195.3919	169097.8079	161513.4324	180971.446	271111.3353	213995.2641
227133.5275	165257.4218	182724.8937	252407.5626	296479.0767	208180.8589
179795.0056	113682.9572	229800.821	186692.3898	229555.409	219883.3603
167662.6399	204134.4029	198109.1262	178262.1658	304309.9981	212195.5793

Fail

Spot 99

Controlrep1	Controlrep2	Controlrep3	Infectrep1	Infectrep2	Infectrep3
68861.65701	75150.90644	46270.11418	120912.7731	120647.2187	67802.56009
61879.45687	87556.40648	60087.87261	123615.8521	148842.1782	97007.82316
75959.82184	106930.6534	41464.66575	144839.0559	140806.2286	84125.55148
61724.6146	133186.618	58613.01056	95065.81799	153625.6821	73878.0826
98821.92091	76322.86881	49985.14439	134261.3314	127313.102	67564.83692
107146.7685	80520.04749	92968.92117	125224.4715	130817.1248	102296.0634

Fail

Spot 102

Controlrep1	Controlrep2	Controlrep3	Infectrep1	Infectrep2	Infectrep3
255232.1421	200858.4496	295476.1343	376308.0506	337543.685	411671.7607
239997.4161	214539.0762	225337.1991	348061.1861	331411.102	365652.5513
174533.4385	360170.4305	191508.1771	278898.6978	567390.7234	275699.969
318255.2918	334368.0819	260334.0256	390425.9863	611733.7293	327991.15
295633.5151	342489.1534	242142.0321	386313.1348	645120.0476	308953.3549
291575.688	213983.2425	426601.7661	350099.5547	337889.4521	567880.1327

Pass

Spot 119	Fail				
Controlrep1	Controlrep2	Controlrep3	Infectrep1	Infectrep2	Infectrep3
137311.5735	88846.84072	93732.79813	242899.3081	190817.3459	158123.6282
119577.5293	128129.2048	72249.50323	231299.7167	285701.3787	175208.0983
124371.9008	135130.5745	94176.99991	259960.5236	297049.5278	194603.3586
230378.8995	194486.385	193415.1039	299499.3896	270112.9194	207517.9999
213513.2513	233368.4719	133697.3675	132985.8501	361699.2579	82383.10428
186983.2595	90081.53982	230173.6568	144036.4712	160117.0463	170745.9403

Spot 126

Controlrep1	Controlrep2	Controlrep3	Infectrep1	Infectrep2	Infectrep3
325223.1732	256747.0777	356435.9469	476919.9462	403572.7067	505225.5384
258803.4481	279465.7845	319689.0643	452469.5333	418134.9663	450652.2321
201674.6801	340610.0335	189316.0001	341557.0665	473340.3925	271813.0185
346084.4047	354758.6507	289446.5411	467384.6686	534597.0483	345155.4837
341796.0192	399161.9728	283785.1038	361884.348	697569.7104	335411.7527
366215.3519	251187.0399	296796.9918	352774.8023	356284.272	427970.038

Pass

Spot 131

Controlrep1	Controlrep2	Controlrep3	Infectrep1	Infectrep2	Infectrep3
126690.8549	156235.1337	102026.2186	211489.3293	268386.4899	158619.532
105773.8702	173494.1858	160652.3121	282601.4351	277246.041	205939.8539
111672.9425	216387.5381	118838.1268	321790.1966	300564.8639	223415.531
116322.2396	254552.3074	158293.4945	122569.9868	244047.1874	116970.556
93280.49366	203700.8121	159306.4154	133671.1709	226446.2712	155663.1261
122259.443	239392.2635	123300.6659	152411.1847	268495.1072	125547.6511

Fail

Spot 140

Controlrep1	Controlrep2	Controlrep3	Infectrep1	Infectrep2	Infectrep3
1505917.401	1536047.635	2266018.82	1191283.665	1152771.521	1811874.514
1464370.969	1850038.134	1775037.241	1299633.764	1323756.171	1745417.613
1218819.961	1784195.213	1365340.349	1168328.306	1277685.692	1289847.098
2635711.511	1826357.367	2108666.069	1485311.23	1076942.115	2012632.287
2237379.278	2050890.363	2217174.274	1339925.347	1189996.536	1569459.641
1878948.182	1283135.504	2483320.003	1064412.261	754764.8798	1872640.722

Fail

Spot 144

Controlrep1	Controlrep2	Controlrep3	Infectrep1	Infectrep2	Infectrep3
164978.9136	149209.5553	108241.6961	172033.6203	181693.4557	168424.9335
92520.14068	180900.4375	158579.6714	125655.5299	176060.6394	215663.656
209536.8535	121607.6018	144663.4908	253598.7278	145165.0612	190039.5431
106273.42	118533.5371	126027.0353	158821.0835	198457.9335	168903.8885
126429.671	113209.6101	101930.0395	149563.3682	201897.8144	116305.4474
129456.4079	139306.0746	152908.612	198797.2679	227340.8585	228795.4099

Pass

Spot 147

Controlrep1	Controlrep2	Controlrep3	Infectrep1	Infectrep2	Infectrep3
179748.327	137246.7816	83619.88501	240713.223	228530.2205	112797.5507
212613.4051	117212.3575	108781.0187	313257.438	214210.1322	130736.3811
168364.6125	107876.7367	139540.7399	240006.0942	188800.444	189769.5564
147453.944	161535.6969	108884.5883	200151.6108	182320.4233	120347.251
204524.9952	135091.8115	131350.0782	271315.6529	191977.1593	148502.6878
236312.8053	162973.8514	138800.2484	262116.5045	212442.2005	133147.6373

Fail

Spot 148

Controlrep1	Controlrep2	Controlrep3	Infectrep1	Infectrep2	Infectrep3
1335869.432	1071094.653	1207061.295	1736100.973	1505439.714	1384524.319
1355575.568	1073296.294	870818.4439	1725749.313	1423295.579	996702.8524
1959000.993	932734.2671	823283.924	2637963.802	1322664.768	1000270.587
1630279.175	730592.3437	1085594.743	2068750.415	1092549.714	1477051.632
1514525.179	1090909.683	806320.7552	2032186.268	1828883.479	1079486.119
1497651.121	836667.9414	1069938.005	1691680.829	1412100.478	1401994.952

Fail

Spot 149 Fail

Controlrep1	Controlrep2	Controlrep3	Infectrep1	Infectrep2	Infectrep3
1625055.132	2371500.38	1587113.698	1459299.365	1852402.402	1430232.917
1474190.474	2674130.925	1745189.39	1437269.902	2105415.101	1514634.604
1344025.113	2480571.374	1267676.643	1322361.108	1900621.446	1298447.482
2214913.107	2721483.645	2405690.773	1474041.608	1798922.952	1799816.872
1594349.44	3153890.875	1856090.579	1040531.261	1982792.837	1245472.418
1361046.557	1801748.058	3054599.089	769425.0257	1174147.885	2016914.332

Spot 153

Controlrep1	Controlrep2	Controlrep3	Infectrep1	Infectrep2	Infectrep3
880544.6597	561905.9268	392752.2763	549134.6438	426043.5133	313232.7406
528737.5462	532757.9148	392308.6642	405818.9979	434260.1803	377870.76
628737.0583	508916.9455	330488.5778	418905.3861	362907.1497	336856.314
763240.1213	401238.0659	425056.9705	574493.7219	371653.888	391081.9541
664790.9966	900844.8966	471772.988	471253.5568	598581.3519	338035.8426
608801.4551	373159.7935	496492.9087	438597.022	275900.3381	400044.4541

Fail

96H HR MEMBRANE

Spot 1

Controlrep1	Controlrep2	Controlrep3	96hrinfectrep1	96hrinfectrep2	96hrinfectrep3
58712.95687	42346.10866	35533.36747	423895.2258	324820.3359	142203.3217
180068.8828	22101.41251	118167.3811	589818.5968	458799.1102	195304.447
66493.3702	44424.08716	65205.66568	338412.4793	341166.1433	168815.4059
39675.03791	51010.86695	94910.02082	360922.2366	226903.4671	92595.88566
47854.52142	25602.60392	54434.98477	264897.8848	150947.8816	51831.78211
133640.1509	40924.57749	54905.69384	533994.3079	188879.316	70988.98851

Fail

Spot 2

Controlrep1	Controlrep2	Controlrep3	96hrinfectrep1	96hrinfectrep2	96hrinfectrep3
594820.6952	689316.5659	1005154.724	3898308.781	4939791.52	2141226.678
509350.6454	761547.9569	994333.0436	4568380.024	6265670.988	1936549.522
498217.4511	1402346.641	915233.8211	4292134.537	5466397.246	2092858.366
539212.5401	857157.549	1648218.297	3339575.791	4667556.13	1901230.322
495076.8798	923338.7251	1045437.788	3031939.106	3787810.154	1323369.846
796972.4313	1318308.538	1275705.356	5444748.421	4499061.595	1209599.613

Fail

Spot 3 Fail

Controlrep1	Controlrep2	Controlrep3	96hrinfectrep1	96hrinfectrep2	96hrinfectrep3
895631.7252	190030.9491	171701.9173	2017098.232	715810.4706	231735.0152
550411.9152	172554.2103	171917.9648	1580769.496	1213462.606	202617.8358
345578.5291	269107.1182	275529.6264	1777765.289	1691750.803	293106.9209
147430.4635	178533.6682	194779.8085	1056424.491	924468.8041	274731.9078
443609.8272	241656.7763	82998.13404	2116852.003	872767.2774	80994.85196
206952.6446	214804.3124	508452.6276	1524970.505	841561.6081	932165.0634

Spot 4

Controlrep1	Controlrep2	Controlrep3	96hrinfectrep1	96hrinfectrep2	96hrinfectrep3
80397.2549	17826.9211	23173.31176	334216.8071	253009.2871	147014.5144
142781.823	23424.2607	120787.32	458061.4944	310099.9682	140452.5627
112616.723	77733.9406	43543.80195	301959.5488	288951.0993	206263.2116
49207.0708	28243.9975	93145.52428	278158.4795	176166.2599	90754.64935
35986.4967	29383.9486	66373.24335	197526.2412	185130.5222	63637.94539
66735.1575	88518.3798	67517.79309	295470.6736	205247.6013	51613.08759

Fail

Spot 5

Controlrep1	Controlrep2	Controlrep3	96hrinfectrep1	96hrinfectrep2	96hrinfectrep3
178501.8621	124831.9776	214711.1486	883420.8934	704960.5994	431959.1448
234915.8171	162774.6009	122329.8414	930437.1355	1018149.647	205293.1286
195368.6701	408198.5028	437578.9023	1209087.158	1296481	631287.1627
239044.0722	213049.3763	232148.1283	554583.5543	851503.2897	369706.3265
156830.0232	201344.565	208805.1779	996721.3763	870671.0605	365066.587
298726.2077	223197.5687	184077.4062	566744.5459	966428.0195	187541.7942

Fail

Spot 6

Controlrep1	Controlrep2	Controlrep3	96hrinfectrep1	96hrinfectrep2	96hrinfectrep3
3095207.92	2151852.957	2882944.687	8316022.079	8278885.269	6325595.193
2516068.88	1992986.61	1606923.622	7978026.852	7040813.723	3587819.525
2540655.03	2516346.186	2726445.266	8724130.364	8486948.012	5400028.811
1637578.35	1657907.592	1132318.77	5198569.957	5255089.573	4170746.095
1742674.06	1866521.576	835650.3989	7268538.821	5713376.5	2724505.738
1870301.75	2006424.961	1295999.214	5518385.794	6283910.258	4341897.013

Pass

Spot 7

Controlrep1	Controlrep2	Controlrep3	96hrinfectrep1	96hrinfectrep2	96hrinfectrep3
248753.235	117098.7306	95960.56519	605819.7506	542432.7209	120869.9691
140348.809	160197.4236	119136.2238	410700.4005	534688.6104	156129.0479
201719.88	89699.0676	88820.99146	551480.9788	456185.9333	138153.7543
193902.578	190865.9808	153369.1811	547358.2276	436888.0943	170698.3881
166652.028	167996.9912	61031.28004	387859.0178	396762.0514	96603.14307
202765.908	201757.5231	130869.3913	482058.3793	427412.6969	74006.91325

Fail

Spot 8

Controlrep1	Controlrep2	Controlrep3	96hrinfectrep1	96hrinfectrep2	96hrinfectrep3
268062.9775	180001.7211	343671.9429	547381.908	371471.5332	498021.9387
193300.4574	162515.163	189625.2218	562004.6853	387196.7056	450590.3406
145341.0349	353821.2175	260072.5395	538939.0956	474513.4648	440724.8399
58900.58327	73542.33478	175387.4537	224587.7545	218286.1218	509783.0891
184378.2231	65051.10297	165948.9234	502484.2025	212523.9962	470426.9994
90883.26256	101714.5817	115126.9607	313574.8693	273620.2676	477198.403

Pass

Spot 9

Controlrep1	Controlrep2	Controlrep3	96hrinfectrep1	96hrinfectrep2	96hrinfectrep3
152711.066	156043.3368	262264.8321	682294.0515	328454.7966	587321.8722
140274.331	176726.0978	137994.3301	558963.2871	494017.8233	315824.4968
196410.661	134951.4459	282014.75	682684.1007	429373.829	679980.1751
160872.146	125492.8765	269694.4032	167852.2794	267631.5368	467300.3628
84737.3903	145073.1086	199068.3225	372858.149	257912.2426	421712.6168
469780.413	131833.3752	153058.0582	541352.62	257521.1192	428329.2157

Pass

Spot 10

Controlrep1	Controlrep2	Controlrep3	96hrinfectrep1	96hrinfectrep2	96hrinfectrep3
734856.0804	817301.9936	1312912.801	1655062.225	1901301.29	2261947.07
959205.1362	940510.1533	820276.7314	1884502.18	2413405.359	1535484.281
1015821.265	831658.9337	1016624.685	2212762.309	2323501.581	1889601.448
436752.4543	538870.1704	384805.9008	1312545.885	1606845.195	1203539.059
458495.1774	685905.6152	848374.9874	1855215.003	1866909.691	1406255.602
800538.5194	601077.8548	517018.816	1255150.638	1805324.353	1736381.234

Pass

Spot 11

Controlrep1	Controlrep2	Controlrep3	96hrinfectrep1	96hrinfectrep2	96hrinfectrep3
111752.3248	193743.1473	194655.0746	563820.6032	697197.6186	215341.1473
210640.1773	158212.2885	385104.1553	936687.179	896279.5295	294961.4751
222687.5699	171189.3894	213556.9323	1207932.3	705950.4884	163532.4094
151247.1576	142111.048	294051.4114	148031.7448	528204.145	266440.2442
226537.1253	216314.9851	232509.0969	832228.201	496588.5086	270833.1911
587349.7633	111059.2116	234967.8061	300921.1963	317005.1852	261004.3664

Fail

Spot 13

Controlrep1	Controlrep2	Controlrep3	96hrinfectrep1	96hrinfectrep2	96hrinfectrep3
407729.7168	122714.1034	107824.0654	715766.7082	366094.5245	311019.631
169353.5826	113873.5072	95845.93226	372496.3954	349374.9451	299832.6086
395650.0269	88508.92853	78837.4664	1024636.168	291344.0993	363827.3701
396621.4005	155591.8731	109641.1117	746096.0463	274991.3817	147152.7653
237805.6137	132179.6294	75870.44012	464971.7026	192545.4747	80589.62042
273728.7874	96095.27515	183520.6488	484979.176	160743.9153	190917.6555

Fail

Spot 14

Controlrep1	Controlrep2	Controlrep3	96hrinfectrep1	96hrinfectrep2	96hrinfectrep3
471839.3583	477781.1261	1145476.399	1162358.823	2022480.384	1924171.932
556129.1125	621738.5997	778298.7267	1209571.044	2508558.886	1531886.087
582558.7561	788992.6245	920836.6884	1465269.939	2196099.731	1961380.132
392650.7217	750576.7291	1547939.461	1065516.035	1874198.554	2021064.28
309777.2215	698250.7947	1268966.195	1139212.312	1515581.422	1625442.47
650562.7005	861054.5882	1397976.071	1561363.772	1485230.41	1485269.62

Fail

Spot 15

Controlrep1	Controlrep2	Controlrep3	96hrinfectrep1	96hrinfectrep2	96hrinfectrep3
269324.0875	146985.404	142650.9494	609888.4353	449135.0421	392776.7311
316100.0555	214925.186	161948.5208	798281.1401	589294.8402	223030.2043
316745.265	148530.974	165418.6814	790860.4638	344691.6556	517055.1753
354088.1101	242249.491	229384.7419	789757.1052	391084.8212	301916.3001
211293.3895	219447.797	240295.3019	531381.7307	328925.2412	272689.6249
337479.2382	205405.968	317187.4883	662093.9207	281005.2567	359427.7766

Fail

Spot 16

Controlrep1	Controlrep2	Controlrep3	96hrinfectrep1	96hrinfectrep2	96hrinfectrep3
145808.4907	30525.96437	119626.2285	439245.3297	206026.3792	261832.0262
184131.4742	105486.2397	160514.5084	504507.829	361132.1607	258378.9398
152779.0494	109938.1676	134482.2997	338297.605	188331.1071	242179.0762
54960.66824	45712.31807	192724.6147	56153.225	155537.8772	106592.7352
86547.9843	66864.26339	79598.98377	221278.0344	157350.8101	56141.0673
121424.7554	65017.97636	198331.6264	226632.1503	252887.9374	86852.69815

Fail

Spot 17

Controlrep1	Controlrep2	Controlrep3	96hrinfectrep1	96hrinfectrep2	96hrinfectrep3
607779.084	282887.8383	138627.4363	321095.3066	186856.632	160415.9851
823605.818	543063.7169	268469.002	271074.4208	291706.4692	123809.6107
1123055.02	332288.2068	99704.27129	480652.428	170731.9628	79285.9201
550316.512	388109.7164	186298.0904	246259.9771	217505.5101	131727.8481
1269520.78	317049.6425	255755.162	556743.0815	206056.8442	206474.8263
464399.27	183825.2141	262158.976	225845.3133	105802.604	71966.90411

Fail

Spot 18

Controlrep1	Controlrep2	Controlrep3	96hrinfectrep1	96hrinfectrep2	96hrinfectrep3
973436.2016	958793.3785	692943.7924	1788543.221	1105351.939	1146993.578
547271.0556	450553.6755	448234.2858	946873.3182	958807.0212	771307.3748
930437.8274	928350.0591	675677.7552	1226743.411	1628683.208	962202.3258
565401.2504	621428.7879	349095.9028	1312151.086	1445627.13	1254034.669
383890.1952	980649.2639	390028.1669	1159769.135	1703400.067	1096179.227
458906.1433	368918.6988	438120.9079	859063.7773	1224473.318	1246271.932

Pass

Spot 20

Controlrep1	Controlrep2	Controlrep3	96hrinfectrep1	96hrinfectrep2	96hrinfectrep3
542848.8057	588962.1649	1002712.119	1374405.163	1249748.825	1093092.594
939060.6023	698936.5473	736896.6684	1592882.772	1408413.34	762824.1723
688259.3901	978132.8443	1343599.94	1919796.329	1423339.358	1991089.049
314416.3793	422927.6796	814310.4766	939641.8042	1144171.299	1385006.637
720201.7847	609522.1169	832047.6623	1887840.087	1443196.226	1326398.139
316905.3238	665150.3117	584381.073	1175855.388	1606621.128	701752.1438

Pass

Spot 22

Controlrep1	Controlrep2	Controlrep3	96hrinfectrep1	96hrinfectrep2	96hrinfectrep3
122892.8831	69200.88535	68258.45059	355610.5312	149195.0918	96786.46166
279714.8202	105953.4573	133069.9557	657391.8062	365894.5804	218075.087
70941.69683	107621.4128	117720.6936	196002.3737	177556.3913	206680.811
84707.05342	124654.4546	83396.93476	186911.5472	238076.6091	92779.11119
147818.4437	110724.771	101406.3081	249463.2982	199763.8543	93036.94859
378244.1785	57903.85916	105144.8871	594150.2408	112829.3217	62809.05677

Fail

Spot 25

Controlrep1	Controlrep2	Controlrep3	96hrinfectrep1	96hrinfectrep2	96hrinfectrep3
355246.3962	69328.65504	152376.914	457852.6235	262419.0188	182863.9147
89949.35882	96406.04116	69510.8723	221459.4794	172490.3158	130184.2724
83741.35532	30253.471	68101.3534	233873.2848	204012.6324	129566.1268
123614.2532	87396.55828	113798.001	170024.5673	157859.9491	104677.0816
69087.73204	86886.29172	56081.6498	76400.23023	125353.6197	41645.15291
77466.63109	103337.2695	127028.66	124713.9857	149645.2443	122446.541

Fail

Spot 26

Controlrep1	Controlrep2	Controlrep3	96hrinfectrep1	96hrinfectrep2	96hrinfectrep3
65492.31704	55765.00425	224772.1788	108721.5585	197342.1018	218299.8884
145872.0277	121980.9735	196916.6386	275042.8589	253250.8192	216929.8452
64949.91344	91863.07846	263632.5646	191890.7651	216248.1272	284257.5582
93625.06434	92463.95203	89436.2863	187508.5662	226629.4642	79510.29719
127419.779	55120.10184	106297.8072	167069.5064	125118.001	112160.8608
69400.90204	61282.04276	244633.0421	186815.4181	155802.4588	298619.6404

Fail

Spot 28

Controlrep1	Controlrep2	Controlrep3	96hrinfectrep1	96hrinfectrep2	96hrinfectrep3
1346989.484	1857110.734	1306100.726	2014901.463	2835278.313	2529835.734
1235938.621	2045247.833	1445438.297	2957571.888	2822670.627	2601559.967
1411475.938	2410547.163	1372387.163	3135760.576	3116190.586	2338840.459
1335476.129	1541563.606	2346890.931	2625031.775	2831671.496	2605398.29
1203414.175	1506110.246	1180599.227	2100124.645	2564572.465	1682201.959
1875926.63	1898179.406	1598400.663	3509541.735	2960264.309	1165197.646

Pass

Spot 30

Controlrep1	Controlrep2	Controlrep3	96hrinfectrep1	96hrinfectrep2	96hrinfectrep3
129432.6538	82307.27452	241866.7732	532744.8741	192214.9382	372164.7617
71629.30327	441032.2839	219966.8095	397322.6812	569912.9349	344920.8983
122392.3343	131871.2677	213716.3589	648536.4075	244373.3682	316162.7805
95748.67618	136227.6249	243828.101	307759.0845	287997.4524	267006.8765
34310.80455	89517.3001	235726.5895	256796.6149	145948.9966	214894.1347
191761.6749	169950.2284	1286928.046	469180.7521	184795.7391	783680.93

Fail

Spot 32

Controlrep1	Controlrep2	Controlrep3	96hrinfectrep1	96hrinfectrep2	96hrinfectrep3
913818.4712	1117994.673	2243589.509	3047430.305	3028906.988	1714486.021
1658392.636	1226746.655	2400266.687	3168818.941	2447653.78	1602873.296
1652963.536	898373.2275	3049594.462	3376157.021	2402591.751	1961050.055
1062915.901	1654794.488	1356812.117	2471081.408	3095055.877	2473392.497
2056993.692	1686648.578	1290488.753	2178783.022	3249558.904	1597304.593
2495334.734	1021236.744	1499978.45	3427806.755	2554826.174	2007128.342

Fail

Spot 34

Controlrep1	Controlrep2	Controlrep3	96hrinfectrep1	96hrinfectrep2	96hrinfectrep3
886606.119	241752.2218	189285.9201	647514.7609	196789.0587	152498.4664
540418.43	468851.1453	305278.7485	310928.6513	400820.4423	330396.241
588100.022	123900.182	291534.29	297412.1728	136289.5664	283117.1382
372905.441	347107.5737	318188.5236	237712.7997	180387.5904	151754.2243
256039.05	414158.1446	352909.4896	127155.3911	216525.7852	185348.3076
339458.933	518967.5474	480034.5569	232561.004	251338.9207	207103.9614

Fail

Spot 36

Controlrep1	Controlrep2	Controlrep3	96hrinfectrep1	96hrinfectrep2	96hrinfectrep3
251555.101	155129.2832	129774.0993	483396.1987	313702.3739	271864.5594
452459.875	160816.6161	249832.874	849357.1621	316814.3641	373147.9372
304054.204	71281.56183	204164.5604	518462.9214	135446.3504	352299.2191
376733.119	239660.8953	197895.3267	577538.8333	297300.0432	245542.698
232213.643	234316.3657	138526.8612	341633.5141	288927.905	182365.8719
421273.889	182895.0075	177205.424	507255.9251	211565.4647	162918.7866

Fail

Spot 38

Controlrep1	Controlrep2	Controlrep3	96hrinfectrep1	96hrinfectrep2	96hrinfectrep3
271923.4125	402974.858	332057.7933	130690.0573	276312	241826.207
427845.3911	392269.703	300366.1457	278847.002	247196.4541	252732.8802
340857.2918	317014.959	354583.9382	151959.4146	179817.3137	265315.4747
305849.7135	380510.623	243224.2309	178493.1106	227682.1592	193058.7636
192411.1057	372913.734	252064.3587	134414.4012	222992.216	261686.7291
212416.0506	403474.367	232767.2447	177025.607	230547.7983	161327.6385

Fail

Spot 56

Controlrep1	Controlrep2	Controlrep3	96hrinfectrep1	96hrinfectrep2	96hrinfectrep3
582505.244	289823.7925	333640.7273	733180.1662	445261.0231	445794.196
368192.135	347331.7561	254039.6668	569991.5529	586353.6711	389778.0858
407810.407	372297.405	362018.1624	616073.1493	619216.3727	597319.7459
437247.32	299463.9362	326138.0254	773572.6302	463603.2352	414838.1424
355804.922	422286.1254	358780.1131	538272.8588	562325.3836	417677.2974
408774.762	388081.19	477503.2136	652612.8387	478040.4526	431809.0167

Fail

Spot 57

Controlrep1	Controlrep2	Controlrep3	96hrinfectrep1	96hrinfectrep2	96hrinfectrep3
2852032.919	2743922.545	2255925.301	3887992.491	3452901.379	2642086.426
2576308.219	2809308.093	1943672.016	3071143.548	3594922.936	1697992.384
3162458.379	2848359.85	1588468.626	4684815.439	3371323.198	1492169.308
1267183.147	1395945.144	1586294.154	2222732.745	2812883.759	2329936.027
1962575.266	1785594.16	1978364.508	3901321.349	3301405.096	2579805.37
1695475.503	1973289.999	870095.2615	3132259.24	3757504.669	1420964.863

Fail

Spot 60

Controlrep1	Controlrep2	Controlrep3	96hrinfectrep1	96hrinfectrep2	96hrinfectrep3
4321351.603	3138973.487	2589060.547	6217386.011	4753830.412	4380873.63
2486722.598	2753300.735	6601318.712	3057057.28	4231598.536	10173712.73
3427118.446	2538838.371	8125013.936	4567438.321	4085116.687	11200221.46
2826094.971	3246729.862	4755620.556	4506323.444	4043099.723	6519807.23
2064729.219	2676372.089	5099217.02	2768396.673	3295672.101	7327704.888
2037587.632	2860463.35	5053529.947	3661474.344	3557474.211	6286143.607

Fail

Spot 82

Controlrep1	Controlrep2	Controlrep3	96hrinfectrep1	96hrinfectrep2	96hrinfectrep3
993546.0215	745508.9209	760821.5119	599963.5586	559805.6542	866108.586
908064.5185	758205.3527	842888.177	688903.1257	604020.531	877941.318
903591.2388	677284.9782	870359.2468	569474.8964	494595.4602	887922.9628
728587.0945	887075.4489	961789.26	511784.0809	601272.2979	707896.7849
919966.1341	973655.9835	686911.7773	561397.5987	512210.505	515923.9826
1183979.218	1048966.786	1075261.523	814026.092	619956.8475	811067.549

Fail

Spot 84

Controlrep1	Controlrep2	Controlrep3	96hrinfectrep1	96hrinfectrep2	96hrinfectrep3
191500.2507	148151.1142	128206.7461	116652.0959	123552.3811	167521.9428
193772.6974	170726.2204	135073.811	103889.9891	140320.6456	119348.8973
220624.5453	194977.7775	173933.9859	92578.8131	177895.3493	160918.141
154975.2028	221703.3254	131753.2399	72662.32126	115898.4249	130867.8048
183558.8056	187595.5145	146508.6253	154171.8305	109001.6171	120863.917
210205.6068	231515.1281	161080.8347	168799.9784	151860.5691	146982.1883

Fail

Spot 87

Controlrep1	Controlrep2	Controlrep3	96hrinfectrep1	96hrinfectrep2	96hrinfectrep3
464578.621	241948.2527	354673.2002	296146.3911	162550.6992	362459.7116
444679.487	279243.449	423061.4343	400320.392	240011.5487	425434.4507
456355.843	230584.0005	524171.6209	366179.5347	183027.676	527910.4982
466770.797	405646.678	376478.8542	318861.9193	225054.0257	285085.7002
443115.059	425206.5428	403325.1304	294748.0621	238041.6867	251668.9117
605987.825	385910.2148	476642.8388	447564.9266	219823.2606	287965.9135

Fail

Spot 88

Controlrep1	Controlrep2	Controlrep3	96hrinfectrep1	96hrinfectrep2	96hrinfectrep3
2908991.851	2182090.341	1721698.807	1950180.908	2059666.242	1706215.35
2748849.495	1932767.01	1936529.2	1968865.99	1571513.666	1850494
2715828.063	1245634.342	1517821.027	1816238.963	1254371.124	1465848.88
2569682.263	2211470.917	1621400.193	1853157.932	1420293.88	1111315.212
2128942.761	1928977.982	2120081.3	1484235.683	1313340.399	1291019.214
2383568.969	2263283.556	2277791.059	1691030.62	1557924.608	1372615.366

Fail

Spot 90

Controlrep1	Controlrep2	Controlrep3	96hrinfectrep1	96hrinfectrep2	96hrinfectrep3
138383.2839	111080.2599	107255.6359	138365.2042	67477.46305	80908.8415
141520.4212	150772.0941	99605.28867	139570.4173	109642.761	99192.07862
138433.4181	103067.9898	86810.54407	137570.989	88473.30916	77479.81671
228120.8284	153788.623	137292.8823	130847.7741	109341.3435	103388.9055
176200.957	132900.2553	120746.7713	102651.8894	100296.8072	73707.17506
227037.2114	192291.9546	153993.5835	137079.088	144786.3044	108305.3544

Fail

Spot 94

Controlrep1	Controlrep2	Controlrep3	96hrinfectrep1	96hrinfectrep2	96hrinfectrep3
1356341.763	1755861.763	996858.651	812497.2294	1205370.539	859735.0273
1451029.919	1545827.697	956801.96	1095777.057	1040077.273	704210.6164
1495290.902	1570781.504	878204.741	1262275.113	1086287.335	865992.5781
980053.8154	1966415.019	998530.06	854811.1891	1478318.895	651203.2594
1119849.774	1415436.324	1181086.41	699041.1409	1066592.056	915205.0116
1205969.59	1863137.29	1797156.36	1062300.179	1378046.891	1598809.875

Fail

Spot 97

Controlrep1	Controlrep2	Controlrep3	96hrinfectrep1	96hrinfectrep2	96hrinfectrep3
1930714.572	1937348.317	1210444.045	1253856.028	1419582.61	1328478.216
2029601.45	1981808.311	1688150.553	1476417.217	1721372.431	1843539.033
2280082.356	1769819.679	1898642.35	1519737.362	1569874.613	2005184.13
2070713.848	2158554.733	1722615.125	1644467.152	1355541.147	1228613.398
1864676.813	2737193.843	1789611.433	1308330.412	1857028.675	1179955.694
2562919.821	2522288.553	2688769.909	1949946.052	1637095.987	1852568.699

Pass

Spot 102

Controlrep1	Controlrep2	Controlrep3	96hrinfectrep1	96hrinfectrep2	96hrinfectrep3
10433520.26	9676933.876	9265681.615	6187787.962	6192603.168	9329916.544
8464129.055	10715868.08	11868035.58	6030974.056	7295991.088	10523563.3
9649370.113	9653850.084	9739223.794	7129359.205	6462830.086	8542521.139
7740492.426	10044820.37	12722279.67	6214177.639	7134487.429	10541436.32
6137692.386	8146469.627	9641470.906	5702349.084	5836561.844	8650386.559
7738916.17	10703324.09	8442092.031	6844088.63	7412475.47	5241907.908

Fail

Spot 112

Controlrep1	Controlrep2	Controlrep3	96hrinfectrep1	96hrinfectrep2	96hrinfectrep3
18982524.47	18455303.89	8753400.117	20003680.16	26064830.17	11644802.93
16032891.82	17627498.29	14712475.63	16418966.18	22653638.02	16751883.72
15893213.25	17335001.82	15753998.23	17419674.06	23590745.59	17803946.73
15289184.58	15555983.03	12192212.52	19174100.74	20426651.73	17262966.83
10396621.99	12692231.18	16660187.7	13667773.51	16904617.47	24625603.72
10992490.89	12184808.51	14349929.33	17006152.64	16265675.43	19716290.64

Fail

Spot 122

Controlrep1	Controlrep2	Controlrep3	96hrinfectrep1	96hrinfectrep2	96hrinfectrep3
657919.684	357094.1928	432516.6	666459.8856	531782.543	707980.5955
427800.588	363005.0396	604146.04	492582.0061	619124.4634	774558.3016
644788.271	367141.2025	383050.6	692895.6638	562776.4521	526441.9096
495387.134	529125.6177	470487.93	689183.9429	665718.723	514610.7627
418380.682	593203.7761	442636.21	505177.8883	756377.9579	400586.3171
607979.816	648323.8609	602900.23	657293.2056	789411.2836	595308.8118

Pass

Spot 124

Controlrep1	Controlrep2	Controlrep3	96hrinfectrep1	96hrinfectrep2	96hrinfectrep3
3700157.869	2892347.81	1784055.138	3544323.965	3897477.349	2784361.007
2363394.034	2740719.52	2055344.727	2847952.833	3484279.326	2745227.799
3927478.598	2431621.13	1726279.951	3709077.597	3611792.259	2400395.626
3024330.586	2682534.98	3311982.003	4201145.931	3431296.106	3448305.688
2452151.106	2547007.99	3217809.624	2809804.105	3458954.198	3612272.208
2758651.537	2619886.1	2802144.946	3589877.65	3845079.326	2133024.417

Fail
