# Supplementary material for: Proteomic and Systems Biology Analysis of the Monocyte Response to Coxiella burnetii Infection
Source: PLoS One. 2013 Aug 21;8(8):e69558. doi: 10.1371/journal.pone.0069558 (PMC3749201; doi:10.1371/journal.pone.0069558)
Supplement: Data S2 — Sequence coverage maps for identified proteins. Red indicates sequence coverage. The underscores seen in certain amino acids indicate possible sites of posttranslational modification. Data is presented for all bioinformatics tools used. (PDF) [file pone.0069558.s007.pdf]

48 hr soluble fraction/spot 10/MnSOD/Mascot
MLSRAVCGTSRQLAPALGYLGSRQKHSLPDLPYDYGALEPHINAQIMQLHHSKHHAAYVNNLNVTEEKYQEA
LAKGDVTAQIALQPALKFNGGGHINHSIFWTNLSPNGGGEPKGELLEAIKRDFGSFDKFKEKLTAASVGVQGS
GWGWLGFNKQRGHLQIAACPNQDPLQGTTGLIPLLGIDVWEHAYYLQYKNVRPDYLKAIWNVINWENVTE
RYMACKK

48 hr soluble fraction/spot 10/MnSOD/X!Hunter
MLSRAVCGTSRQLAPALGYLGSRQKHSLPDLPYDYGALEPHINAQIMQLHHSKHHAAYVNNLNVTEEKYQEALAKGD
VTAQIALQPALKFNGGGHINHSIFWTNLSPNGGGEPKGELLEAIKRDFGSFDKFKEKLTAASVGVQGSGWGWLGFNKE
RGHLQIAACPNQDPLQGTTGLIPLLGIDVWEHAYYLQYKNVRPDYLKAIWNVINWENVTERYMACKK

48 hr soluble fraction/spot 10/MnSOD/X!Tandem P3
MLSRAVCGTSRQLAPALGYLGSRQKHSLPDLPYDYGALEPHINAQIMQLHHSKHHAAYVNNLNVTEEKYQEALAKGD
VTAQIALQPALKFNGGGHINHSIFWTNLSPNGGGEPKGELLEAIKRDFGSFDKFKEKLTAASVGVQGSGWGWLGFNKE
RGHLQIAACPNQDPLQGTTGLIPLLGIDVWEHAYYLQYKNVRPDYLKAIWNVINWENVTERYMACKK

96 hr soluble fraction/Spot 2/S100 Ca^{2+} binding protein A9/Mascot

MTCKMSQLERNIETIINTFHQYSVKLGHPDTLNQGEFKELVRKDLQNFLKKENKNEKVIEHIMEDLDTNADKQLSFEEFI
MLMARLTWASHEKMHEGDEGPGHHHKPGLGEGTP

96 hr soluble fraction/Spot 2/S100 Ca^{2+} binding protein A9/X!Hunter

MTCKMSQLERNIETIINTFHQYSVKLGHPDTLNQGEFKELVRKDLQNFLKKENKNEKVIEHIMEDLDTNADKQLSFEEFI
MLMARLTWASHEKMHEGDEGPGHHHKPGLGEGTP

96 hr soluble fraction/Spot 2/S100 Ca^{2+} binding protein A9/X!Tandem P3

MTCKMSQLERNIETIINTFHQYSVKLGHPDTLNQGEFKELVRKDLQNFLKKENKNEKVIEHIMEDLDTNADKQLSFEEFI
MLMARLTWASHEKMHEGDEGPGHHHKPGLGEGTP

96 hr soluble fraction/Spot 5/Visfatin,PBEF/Mascot
MNPAAEAEFNILLATDSYKVTHYKQYPPNTSKVYSYFECREKKTENSKLRKVKYEETVFYGLQYILNKYLKGKVV
TKEKIQEAKDVYKEHFQDDVFNEKGWNYILEKYDGHLPIEIKAVPEGFVIPRGNVLFTVENTDPECYWLTNWIE
TILVQSWYPITVATNSREQKKILAKYLLETSGNLDGLEYKLHDFGYRGVSSQETAGIGASAHLVNFKGTDTVAG
LALIKKYYGTKDPVPGYSVPAAEHSTITAWGKDHEKDAFEHIVTQFSSVPVSVVSDSYDIYNACEKIWGEDLRH
LIVSRSTQAPLIIRPDSGNPLDTVLKVLEILGKKFPVTENSKGYKLLPPYLRVIQGDGVDINTLQEIVEGMKQKM
WSIENIAFGSGGGLLQKLTRDLLNCSFKCSYVVTNGLGINVFKDPVADPNKRSKKGRLSLHRTPAGNFVTLEEG
KGDLEEYGQDLLHTVFKNGKVTKSYSFDEIRKNAQLNIELEAAHH

96 hr soluble fraction/Spot 5/Visfatin,PBEF/X!Hunter
MNPAAEAEFNILLATDSYKVTHYKQYPPNTSKVYSYFECREKKTENSKLRKVKYEETVFYGLQYILNKYLKGKVVTKEKIQE
AKDVYKEHFQDDVFNEKGWNYILEKYDGHLPIEIKAVPEGFVIPRGNVLFTVENTDPECYWLTNWIETILVQSWYPITVA
TNSREQKKILAKYLLETSGNLDGLEYKLHDFGYRGVSSQETAGIGASAHLVNFKGTDTVAGLALIKKYYGTKDPVPGYSVP
AAEHSTITAWGKDHEKDAFEHIVTQFSSVPVSVVSDSYDIYNACEKIWGEDLRHLIVSRSTQAPLIIRPDSGNPLDTVLKV
LEILGKKFPVTENSKGYKLLPPYLRVIQGDGVDINTLQEIVEGMKQKMWSIENIAFGSGGGLLQKLTRDLLNCSFKCSYVV
TNGLGINVFKDPVADPNKRSKKGRLSLHRTPAGNFVTLEEGKGDLEEYGQDLLHTVFKNGKVTKSYSFDEIRKNAQLNIE
LEAAHH

96 hr soluble fraction/Spot 5/Visfatin,PBEF/X!Hunter P3
MNPAAEAEFNILLATDSYKVTHYKQYPPNTSKVYSYFECREKKTENSKLRKVKYEETVFYGLQYILNKYLKGKVVTKEKIQE
AKDVYKEHFQDDVFNEKGWNYILEKYDGHLPIEIKAVPEGFVIPRGNVLFTVENTDPECYWLTNWIETILVQSWYPITVA
TNSREQKKILAKYLLETSGNLDGLEYKLHDFGYRGVSSQETAGIGASAHLVNFKGTDTVAGLALIKKYYGTKDPVPGYSVP
AAEHSTITAWGKDHEKDAFEHIVTQFSSVPVSVVSDSYDIYNACEKIWGEDLRHLIVSRSTQAPLIIRPDSGNPLDTVLKV
LEILGKKFPVTENSKGYKLLPPYLRVIQGDGVDINTLQEIVEGMKQKMWSIENIAFGSGGGLLQKLTRDLLNCSFKCSYVV
TNGLGINVFKDPVADPNKRSKKGRLSLHRTPAGNFVTLEEGKGDLEEYGQDLLHTVFKNGKVTKSYSFDEIRKNAQLNIE
LEAAHH

96 hr soluble fraction/Spot 9/S100 Ca^{2+} binding protein A9/Mascot
MTCKMSQLERNIETIINTFHQYSVKLGHPDTLNQGEFKELVRKDLQNFLKKENKNEKVIEHIMEDLDTNADKQ
LSFEEFIMLMARLTWASHEKMHEGDEGPGHHHKPGLGEGTP

96 hr soluble fraction/Spot 9/S100 Ca^{2+} binding protein A9/X!Hunter
MLTELEKALNSIIDVYHKYSLIKGNFHAVYRDDLKKLLETECPQYIRKKGADVWFKELDINTDGAVNFQEFLILVIKMGVA
AHKKSHEESHKE

96 hr soluble fraction/Spot 9/S100 Ca^{2+} binding protein A9/X!Tandem P3
MLTELEKALNSIIDVYHKYSLIKGNFHAVYRDDLKKLLETECPQYIRKKGADVWFKELDINTDGAVNFQEFLILVIKMGVA
AHKKSHEESHKE

96 hr soluble fraction/Spot 14/S100 Ca^{2+} binding protein A8/Mascot

MLTELEKALNSIIDVYHKYSLIKGNFHAVYRDDLKKLLETECPQYIRKKGADVWFKELDINTDGAVNFQEFLILVI
KMGWQPTKKAMKKATKSS

96 hr soluble fraction/Spot 9/S100 Ca^{2+} binding protein A9/X!Hunter

MLTELEKALNSIIDVYHKYSLIKGNFHAVYRDDLKKLLETECPQYIRKKGADVWFKELDINTDGAVNFQEFLILVIKMGVA
AHKKSHEESHKE

96 hr soluble fraction/Spot 9/S100 Ca^{2+} binding protein A9/X!Tandem P3

MLTELEKALNSIIDVYHKYSLIKGNFHAVYRDDLKKLLETECPQYIRKKGADVWFKELDINTDGAVNFQEFLILVIKMGVA
AHKKSHEESHKE

96 hr soluble fraction/Spot 48/Transaldolase 1/Mascot
MSSSPVKRQRMESALDQLKQFTTVVADTGDFHAIDEYKPQDATTNPSLILAAAQMPAYQELVEEAIAYGRKL
GGSQEDQIKNAIDKLFVLFGAEILKKIPGRVSTEVDARLSFDKDAMVARARRLIELYKEAGISKDRILIKLSSTWE
GIQAGKELEEQHGIHCNMTLLFSFAQAVACAEAGVTLISPFVGRILDWHVANTDKKSYEPLEDPGVKSVTKIYN
YYKKFSYKTIVMGASFRNTGEIKALAGCDFLTISPKLLGELLQDNAKLVPVLSAKAAQASDLEKIHLDEKSFRWL
HNEDQMAVEKLSDGIRKFAADAVKLERMLTERMFNAENGK

96 hr soluble fraction/Spot 48/Transaldolase 1/X!Hunter
MSSSPVKRQRMESALDQLKQFTTVVADTGDFHAIDEYKPQDATTNPSLILAAAQMPAYQELVEEAIAYGRKLGGSQED
QIKNAIDKLFVLFGAEILKKIPGRVSTEVDARLSFDKDAMVARARRLIELYKEAGISKDRILIKLSSTWEGIQAGKELEEQHG
IHCNMTLLFSFAQAVACAEAGVTLISPFVGRILDWHVANTDKKSYEPLEDPGVKSVTKIYNYYKKFSYKTIVMGASFRNT
GEIKALAGCDFLTISPKLLGELLQDNAKLVPVLSAKAAQASDLEKIHLDEKSFRWLHNEDQMAVEKLSDGIRKFAADAVK
LERMLTERMFNAENGK

96 hr soluble fraction/Spot 48/Transaldolase 1/X!Tandem P3
MSSSPVKRQRMESALDQLKQFTTVVADTGDFHAIDEYKPQDATTNPSLILAAAQMPAYQELVEEAIAYGRKLGGSQED
QIKNAIDKLFVLFGAEILKKIPGRVSTEVDARLSFDKDAMVARARRLIELYKEAGISKDRILIKLSSTWEGIQAGKELEEQHG
IHCNMTLLFSFAQAVACAEAGVTLISPFVGRILDWHVANTDKKSYEPLEDPGVKSVTKIYNYYKKFSYKTIVMGASFRNT
GEIKALAGCDFLTISPKLLGELLQDNAKLVPVLSAKAAQASDLEKIHLDEKSFRWLHNEDQMAVEKLSDGIRKFAADAVK
LERMLTERMFNAENGK

96 hr soluble fraction/Spot 60/Hsp60/Mascot
MLRLPTVFRQMRPVSRVLAPHLTRAYAKDVKFGADARALMLQGVDLLADAVAVTMGPKGRTVIIEQSWGS
PKVTKDGVTVAKSIDLKDKYKNIGAKLVQDVANNTNEEAGDGTTTATVLARSIAKEGFEKISKGANPVEIRRGV
MLAVDAVIAELKKQSKPVTTPEEIAQVATISANGDKEIGNIISDAMKKVGRKGVITVKDGKTLNDELEIIEGMKF
DRGYISPYFINTSKGQKCEFQDAYVLLSEKKISSIQSIVPALEIANAHRKPLVIIAEDVDGEALSTLVLNRLKVGLQ
VVAVKAPGFGDNRKNQLKDMAIATGGAVFGEEGLTLNLEDVQPHDLGKVGEVIVTKDDAMLLKGKGDKAQI
EKRIQEIIEQLDVTTSEYEKEKLNERLAKLSDGVAVLKVGGTSDVEVNEKKDRVTDALNATRAAVEEGIVLGGG
CALLRCIPALDSLTPANEDQKIGIEIIKRTLKIPAMTIAKNAGVEGSLIVEKIMQSSSEVGYDAMAGDFVNMVEK
GIIDPTKVVRTALLDAAGVASLLTTAEVVVTEIPKEEKDPGMGAMGGMGGGMGGGMF

96 hr soluble fraction/Spot 60/Hsp60/X!Hunter
MLRLPTVFRQMRPVSRVLAPHLTRAYAKDVKFGADARALMLQGVDLLADAVAVTMGPKGRTVIIEQSWGSPKVTKD
GVTVAKSIDLKDKYKNIGAKLVQDVANNTNEEAGDGTTTATVLARSIAKEGFEKISKGANPVEIRRGVMLAVDAVIAELK
KQSKPVTTPEEIAQVATISANGDKEIGNIISDAMKKVGRKGVITVKDGKTLNDELEIIEGMKFDRGYISPYFINTSKGQKCE
FQDAYVLLSEKKISSIQSIVPALEIANAHRKPLVIIAEDVDGEALSTLVLNRLKVGLQVVAVKAPGFGDNRKNQLKDMAIA
TGGAVFGEEGLTLNLEDVQPHDLGKVGEVIVTKDDAMLLKGKGDKAQIEKRIQEIIEQLDVTTSEYEKEKLNERLAKLSD
GVAVLKVGGTSDVEVNEKKDRVTDALNATRAAVEEGIVLGGGCALLRCIPALDSLTPANEDQKIGIEIIKRTLKIPAMTIAK
NAGVEGSLIVEKIMQSSSEVGYDAMAGDFVNMVEKGIIDPTKVVRTALLAAGVASLLT
TAEVVVTEIPKEEKDPGMGAMGGMGGGMGGGMF

96 hr soluble fraction/Spot 60/Hsp60/X!Tandem P3
MLRLPTVFRQMRPVSRVLAPHLTRAYAKDVKFGADARALMLQGVDLLADAVAVTMGPKGRTVIIEQSWGSPKVTKD
GVTVAKSIDLKDKYKNIGAKLVQDVANNTNEEAGDGTTTATVLARSIAKEGFEKISKGANPVEIRRGVMLAVDAVIAELK
KQSKPVTTPEEIAQVATISANGDKEIGNIISDAMKKVGRKGVITVKDGKTLNDELEIIEGMKFDRGYISPYFINTSKGQKCE
FQDAYVLLSEKKISSIQSIVPALEIANAHRKPLVIIAEDVDGEALSTLVLNRLKVGLQVVAVKAPGFGDNRKNQLKDMAIA
TGGAVFGEEGLTLNLEDVQPHDLGKVGEVIVTKDDAMLLKGKGDKAQIEKRIQEIIEQLDVTTSEYEKEKLNERLAKLSD
GVAVLKVGGTSDVEVNEKKDRVTDALNATRAAVEEGIVLGGGCALLRCIPALDSLTPANEDQKIGIEIIKRTLKIPAMTIAK
NAGVEGSLIVEKIMQSSSEVGYDAMAGDFVNMVEKGIIDPTKVVRTALLDAAGVASLLTTAEVVVTEIPKEEKDPGMG
AMGGMGGGMGGGMF

96 hr soluble fraction/Spot 63/Inorganic pyrophosphatase/Mascot
STEERAAAFSLEYRVFLKNEKGQYISPFHDIPIYADKDVFHMVVEVPRWSNAKMEIATKDPLNPIKQDV
KKGKLRYVANLFPYKGYIWNYGAIPQTWEDPGHNDKHTGCCGDNDPIDVCEIGSKVCARGEIIGVKVLGILA
MIDEGETDWKVIAINVDDPDAANYNDINDVKRLKPGYLEATVDWFRRYKVPDGKPENEFAFNAEFKDKDFAI
DIIKSTHDHWKALVTKKTNGKGISCMNTTLSESPFKCDPDAARAIVDALPPPCESACTVPTDVDKWFHH

96 hr soluble fraction/Spot 63/Inorganic pyrophosphatase/X!Hunter
MSGFSTEERAAPFSLEYRVFLKNEKGQYISPFHDIPIYADKDVFHMVVEVPRWSNAKMEIATKDPLNPIKQDVKKGKLR
YVANLFPYKGYIWNYGAIPQTWEDPGHNDKHTGCCGDNDPIDVCEIGSKVCARGEIIGVKVLGILAMIDEGETDWKVI
AINVDDPDAANYNDINDVKRLKPGYLEATVDWFRRYKVPDGKPENEFAFNAEFKDKDFAIDIIKSTHDHWKALVTKKT
NGKGISCMNTTLSESPFKCDPDAARAIVDALPPPCESACTVPTDVDKWFHHQKN

96 hr soluble fraction/Spot 63/Inorganic pyrophosphatase/X!Tandem P3
MSGFSTEERAAPFSLEYRVFLKNEKGQYISPFHDIPIYADKDVFHMVVEVPRWSNAKMEIATKDPLNPIKQDVKKGKLR
YVANLFPYKGYIWNYGAIPQTWEDPGHNDKHTGCCGDNDPIDVCEIGSKVCARGEIIGVKVLGILAMIDEGETDWKVI
AINVDDPDAANYNDINDVKRLKPGYLEATVDWFRRYKVPDGKPENEFAFNAEFKDKDFAIDIIKSTHDHWKALVTKKT
NGKGISCMNTTLSESPFKCDPDAARAIVDALPPPCESACTVPTDVDKWFHHQKN

96 hr soluble fraction/Spot 69/EB1/Mascot
MAVNVYSTSVTSDNLSRHDMLAWINESLQLNLTKIEQLCSGAAYCQFMDMLFPGSIALKKVKFQAKLEHEYI
QNFKILQAGFKRMGVDKIIPVDKLVKGKFQDNFEFVQWFKKFFDANYDGKDYDPVAARQGQETAVAPSLVA
PALNKPKKPLTSSSAAPQRPISTQRTAAAPKAGPGVVRKNPGVGNGDDEAAELMQQVNVLKLTVEDLEKER
DFYFGKLRNIELICQENEGENDPVLQRIVDILYATDEGFVIPDEGGPQEEQEEY

96 hr soluble fraction/Spot 69/EB1/X!Hunter
MAVNVYSTSVTSDNLSRHDMLAWINESLQLNLTKIEQLCSGAAYCQFMDMLFPGSIALKKVKFQAKLEHEYIQNFKILQ
AGFKRMGVDKIIPVDKLVKGKFQDNFEFVQWFKKFFDANYDGKDYDPVAARQGQETAVAPSLVAPALNKPKKPLTSSS
AAPQRPISTQRTAAAPKAGPGVVRKNPGVGNGDDEAAELMQQVNVLKLTVEDLEKERDFYFGKLRNIELICQENEGEN
DPVLQRIVDILYATDEGFVIPDEGGPQEEQEEY

96 hr soluble fraction/Spot 69/EB1/X!Tandem P3
MAVNVYSTSVTSDNLSRHDMLAWINESLQLNLTKIEQLCSGAAYCQFMDMLFPGSIALKKVKFQAKLEHEYIQNFKILQ
AGFKRMGVDKIIPVDKLVKGKFQDNFEFVQWFKKFFDANYDGKDYDPVAARQGQETAVAPSLVAPALNKPKKPLTSSS
AAPQRPISTQRTAAAPKAGPGVVRKNPGVGNGDDEAAELMQQVNVLKLTVEDLEKERDFYFGKLRNIELICQENEGEN
DPVLQRIVDILYATDEGFVIPDEGGPQEEQEEY

96 hr soluble fraction/Spot 87/Fascin 1/Mascot

MTANGTAEAVQIQFGLINCGNKYLTAEAFGFKVNASASSLKKKQIWTLEQPPDEAGSAAVCLRSHLGRYLAA
DKDGNVTCEREVPGPDCRFLIVAHDDGRWSLQSEAHRRYFGGTEDRLSCFAQTVSPAEKWSVHIAMHPQV
NIYSVTRKRYAHLSARPADEIAVDRDVPWGVDSLITLAFQDQRYSVQTADHRFLRHDGRLVARPEPATGYTLE
FRSGKVAFRDCEGRYLAPSGPSGTLKAGKATKVGKDELFALEQSCAQVVLQAANERNVSTRQGMDLSANQD
EETDQETFQLEIDRDTKKCAFRTHTGKYWTLTATGGVQSTASSKNASCYFDIEWRDRRITLRASNGKFVTSKK
NGQLAASVETAGDSELFLMKLINRPIIVFRGEHGFIGCRKVTGTLDANRSSYDVFQLEFNDGAYNIKDSTGKYW
TVGSDSAVTSSGDTPVDFFFEFCDYNKVAIKVGGRYLKGDHAGVLKASAETVDPASLWEY

96 hr soluble fraction/Spot 87/Fascin 1/X!Hunter

MTANGTAEAVQIQFGLINCGNKYLTAEAFGFKVNASASSLKKKQIWTLEQPPDEAGSAAVCLRSHLGRYLAADKDGNV
TCEREVPGPDCRFLIVAHDDGRWSLQSEAHRRYFGGTEDRLSCFAQTVSPAEKWSVHIAMHPQVNIYSVTRKRYAHLS
ARPADEIAVDRDVPWGVDSLITLAFQDQRYSVQTADHRFLRHDGRLVARPEPATGYTLEFRSGKVAFRDCEGRYLAPS
GPSGTLKAGKATKVGKDELFALEQSCAQVVLQAANERNVSTRQGMDLSANQDEETDQETFQLEIDRDTKKCAFRTHT
GKYWTLTATGGVQSTASSKNASCYFDIEWRDRRITLRASNGKFVTSKKNGQLAASVETAGDSELFLMKLINRPIIVFRGE
HGFIGCRKVTGTLDANRSSYDVFQLEFNDGAYNIKDSTGKYWTVGSDSAVTSSGDTPVDFFFEFCDYNKVAIKVGGRYL
KGDHAGVLKASAETVDPASLWEY

96 hr soluble fraction/Spot 87/Fascin 1/X!Tandem P3

MTANGTAEAVQIQFGLINCGNKYLTAEAFGFKVNASASSLKKKQIWTLEQPPDEAGSAAVCLRSHLGRYLAADKDGNV
TCEREVPGPDCRFLIVAHDDGRWSLQSEAHRRYFGGTEDRLSCFAQTVSPAEKWSVHIAMHPQVNIYSVTRKRYAHLS
ARPADEIAVDRDVPWGVDSLITLAFQDQRYSVQTADHRFLRHDGRLVARPEPATGYTLEFRSGKVAFRDCEGRYLAPS
GPSGTLKAGKATKVGKDELFALEQSCAQVVLQAANERNVSTRQGMDLSANQDEETDQETFQLEIDRDTKKCAFRTHT
GKYWTLTATGGVQSTASSKNASCYFDIEWRDRRITLRASNGKFVTSKKNGQLAASVETAGDSELFLMKLINRPIIVFRGE
HGFIGCRKVTGTLDANRSSYDVFQLEFNDGAYNIKDSTGKYWTVGSDSAVTSSGDTPVDFFFEFCDYNKVAIKVGGRYL
KGDHAGVLKASAETVDPASLWEY

96 hr soluble fraction/Spot 102/Leucine aminopeptidase 3/Mascot
MFLLPLPAAGRVVVRRLAVVRSGSRSLSTADMTKGLVLGIYSKEKEDDVPQFTSAGENFDKLLAGKLRETLNISGPPLKA
GKTRTFYGLHQDFPSVVLVGLGKKAAGIDEQENWHEGKENIRAAVAAGCRQIQDLELSSVEVDPCGDAQAAAEGAV
LGLYEYDDLKQKKKMAVSAKLYGSGDQEAWQKGVLFASGQNLARQLMETPANEMTPTRFAEIIEKNLKSASSKTEVH
IRPKSWIEEQAMGSFLSVAKGSDEPPVFLEIHYKGSPNANEPPLVFVGKGITFDSGGISIKASANMDLMRADMGGAAT
ICSAIVSAAKLNLPINIIGLAPLCENMPSGKANKPGDVVRAKNGKTIQVDNTDAEGRLILADALCYAHTFNPKVILNAAT
LTGAMDVALGSGATGVFTNSSWLWNKLFEASIETGDRVWRMPLFEHYTRQVVDCQLADVNNIGKYRSAGACTAAA
FLKEFVTHPKWAHLDIAGVMTNKDEVPYLRKGMTGRPTRTLIEFLLRFSQDNA

96 hr soluble fraction/Spot 102/Leucine aminopeptidase 3/X!Hunter
MFLLPLPAAGRVVVRRLAVRRFGSRSLSTADMTKGLVLGIYSKEKEDDVPQFTSAGENFDKLLAGKLRETLNISGPPLKA
GKTRTFYGLHQDFPSVVLVGLGKKAAGIDEQENWHEGKENIRAAVAAGCRQIQDLELSSVEVDPCGDAQAAAEGAVL
GLYEYDDLKQKKKMAVSAKLYGSGDQEAWQKGVLFASGQNLARQLMETPANEMTPTRFAEIIEKNLKSASSKTEVHIR
PKSWIEEQAMGSFLSVAKGSDEPPVFLEIHYKGSPNANEPPLVFVGKGITFDSGGISIKASANMDLMRADMGGAATICS
AIVSAAKLNLPINIIGLAPLCENMPSGKANKPGDVVRAKNGKTIQVDNTDAEGRLILADALCYAHTFNPKVILNAATLTG
AMDVALGSGATGVFTNSSWLWNKLFEASIETGDRVWRMPLFEHYTRQVVDCQLADVNNIGKYRSAGACTAAAFLKE
FVTHPKWAHLDIAGVMTNKDEVPYLRKGMTGRPTRTLIEFLLRFSQDNA

96 hr soluble fraction/Spot 102/Leucine aminopeptidase 3/X!Tandem P3
MFLLPLPAAGRVVVRRLAVRRFGSRSLSTADMTKGLVLGIYSKEKEDDVPQFTSAGENFDKLLAGKLRETLNISGPPLKA
GKTRTFYGLHQDFPSVVLVGLGKKAAGIDEQENWHEGKENIRAAVAAGCRQIQDLELSSVEVDPCGDAQAAAEGAVL
GLYEYDDLKQKKKMAVSAKLYGSGDQEAWQKGVLFASGQNLARQLMETPANEMTPTRFAEIIEKNLKSASSKTEVHIR
PKSWIEEQAMGSFLSVAKGSDEPPVFLEIHYKGSPNANEPPLVFVGKGITFDSGGISIKASANMDLMRADMGGAATICS
AIVSAAKLNLPINIIGLAPLCENMPSGKANKPGDVVRAKNGKTIQVDNTDAEGRLILADALCYAHTFNPKVILNAATLTG
AMDVALGSGATGVFTNSSWLWNKLFEASIETGDRVWRMPLFEHYTRQVVDCQLADVNNIGKYRSAGACTAAAFLKE
FVTHPKWAHLDIAGVMTNKDEVPYLRKGMTGRPTRTLIEFLLRFSQDNA

96 hr soluble farction/Spot 126/Leucine aminopeptidase 3/Mascot
MFLLPLPAAGRVVVRRLAVVRSGSRSLSTADMTKGLVLGIYSKEKEDDVPQFTSAGENFDKLLAGKLRETLNISGPPLKA
GKTRTFYGLHQDFPSVVLVGLGKKAAGIDEQENWHEGKENIRAAVAAGCRQIQDLELSSVEVDPCGDAQAAAEGAV
LGLYEYDDLKQKKKMAVSAKLYGSGDQEAWQKGVLFASGQNLARQLMETPANEMTPTRFAEIIEKNLKSASSKTEVH
IRPKSWIEEQAMGSFLSVAKGSDEPPVFLEIHYKGSPNANEPPLVFVGKGITFDSGGISIKASANMDLMRADMGGAAT
ICSAIVSAAKLNLPINIIGLAPLCENMPSGKANKPGDVVRAKNGKTIQVDNTDAEGRLILADALCYAHTFNPKVILNAAT
LTGAMDVALGSGATGVFTNSSWLWNKLFEASIETGDRVWRMPLFEHYTRQVVDCQLADVNNIGKYRSAGACTAAA
FLKEFVTHPKWAHLDIAGVMTNKDEVPYLRKGMTGRPTRTLIEFLLRFSQDNA

96 hr soluble farction/Spot 126/Leucine aminopeptidase 3/X!Hunter
MFLLPLPAAGRVVVRRLAVRRFGSRSLSTADMTKGLVLGIYSKEKEDDVPQFTSAGENFDKLLAGKLRETLNISGPPLKA
GKTRTFYGLHQDFPSVVLVGLGKKAAGIDEQENWHEGKENIRAAVAAGCRQIQDLELSSVEVDPCGDAQAAAEGAVL
GLYEYDDLKQKKKMAVSAKLYGSGDQEAWQKGVLFASGQNLARQLMETPANEMTPTRFAEIIEKNLKSASSKTEVHIR
PKSWIEEQAMGSFLSVAKGSDEPPVFLEIHYKGSPNANEPPLVFVGKGITFDSGGISIKASANMDLMRADMGGAATICS
AIVSAAKLNLPINIIGLAPLCENMPSGKANKPGDVVRAKNGKTIQVDNTDAEGRLILADALCYAHTFNPKVILNAATLTG
AMDVALGSGATGVFTNSSWLWNKLFEASIETGDRVWRMPLFEHYTRQVVDCQLADVNNIGKYRSAGACTAAAFLKE
FVTHPKWAHLDIAGVMTNKDEVPYLRKGMTGRPTRTLIEFLLRFSQDNA

96 hr soluble farction/Spot 126/Leucine aminopeptidase 3/X!Tandem P3
MFLLPLPAAGRVVVRRLAVRRFGSRSLSTADMTKGLVLGIYSKEKEDDVPQFTSAGENFDKLLAGKLRETLNISGPPLKA
GKTRTFYGLHQDFPSVVLVGLGKKAAGIDEQENWHEGKENIRAAVAAGCRQIQDLELSSVEVDPCGDAQAAAEGAVL
GLYEYDDLKQKKKMAVSAKLYGSGDQEAWQKGVLFASGQNLARQLMETPANEMTPTRFAEIIEKNLKSASSKTEVHIR
PKSWIEEQAMGSFLSVAKGSDEPPVFLEIHYKGSPNANEPPLVFVGKGITFDSGGISIKASANMDLMRADMGGAATICS
AIVSAAKLNLPINIIGLAPLCENMPSGKANKPGDVVRAKNGKTIQVDNTDAEGRLILADALCYAHTFNPKVILNAATLTG
AMDVALGSGATGVFTNSSWLWNKLFEASIETGDRVWRMPLFEHYTRQVVDCQLADVNNIGKYRSAGACTAAAFLKE
FVTHPKWAHLDIAGVMTNKDEVPYLRKGMTGRPTRTLIEFLLRFSQDNA

96 hr soluble fraction/Spot 144/Pyrophosphatase 1/Mascot
STEERAAAFSLEYRVFLKNEKGQYISPFHDIPIYADKDVFHMVVEVPRWSNAKMEIATKDPLNPIKQDVKKGKLRYVAN
LFPYKGYIWNYGAIPQTWEDPGHNDKHTGCCGDNDPIDVCEIGSKVCARGEIIGVKVLGILAMIDEGETDWKVIAINV
DDPDAANYNDINDVKRLKPGYLEATVDWFRRYKVPDGKPENEFAFNAEFKDKDFAIDIIKSTHDHWKALVTKKTNGK
GISCMNTTLSESPFKCDPDAARAIVDALPPPCESACTVPTDVDKWFHH

96 hr soluble fraction/Spot 144/Pyrophosphatase 1/X!Hunter
MSGFSTEERAAPFSLEYRVFLKNEKGQYISPFHDIPIYADKDVFHMVVEVPRWSNAKMEIATKDPLNPIKQDVKKGKLR
YVANLFPYKGYIWNYGAIPQTWEDPGHNDKHTGCCGDNDPIDVCEIGSKVCARGEIIGVKVLGILAMIDEGETDWKVI
AINVDDPDAANYNDINDVKRLKPGYLEATVDWFRRYKVPDGKPENEFAFNAEFKDKDFAIDIIKSTHDHWKALVTKKT
NGKGISCMNTTLSESPFKCDPDAARAIVDALPPPCESACTVPTDVDKWFHHQKN

96 hr soluble fraction/Spot 144/Pyrophosphatase 1/X!Tandem P3
MSGFSTEERAAPFSLEYRVFLKNEKGQYISPFHDIPIYADKDVFHMVVEVPRWSNAKMEIATKDPLNPIKQDVKKGKLR
YVANLFPYKGYIWNYGAIPQTWEDPGHNDKHTGCCGDNDPIDVCEIGSKVCARGEIIGVKVLGILAMIDEGETDWKVI
AINVDDPDAANYNDINDVKRLKPGYLEATVDWFRRYKVPDGKPENEFAFNAEFKDKDFAIDIIKSTHDHWKALVTKKT
NGKGISCMNTTLSESPFKCDPDAARAIVDALPPPCESACTVPTDVDKWFHHQKN

96 hr membrane fraction/Spot 6/MnSOD/Mascot
LSPAVCGTSRHLAPVLGYLGSRQKHSLPDLPYDYGALEPHINAQIMQLHHSKHHAAYVNNLNVTEEKYQEALAKGDVT
AQIALQPALKFNGGGHINHSIFWTNLSPNGGGEPKGELLEAIKRDFGSFDKFKEKLTAASVGVQGSGWGWLGFNKER
GHLQIAACPNQDPLQGTTGLIPLLGIDVWEHAYYLQYKNVRPDYLKAIWNVINWENVT

96 hr membrane fraction/Spot 6/MnSOD/X!Hunter
MLSRAVCGTSRQLAPALGYLGSRQKHSLPDLPYDYGALEPHINAQIMQLHHSKHHAAYVNNLNVTEEKYQEALAKGD
VTAQIALQPALKFNGGGHINHSIFWTNLSPNGGGEPKGELLEAIKRDFGSFDKFKEKLTAASVGVQGSGWGWLGFNKE
RGHLQIAACPNQDPLQGTTGLIPLLGIDVWEHAYYLQYKNVRPDYLKAIWNVINWENVTERYMACKK

96 hr membrane fraction/Spot 6/MnSOD/X!Tandem P3
MLSRAVCGTSRQLAPALGYLGSRQKHSLPDLPYDYGALEPHINAQIMQLHHSKHHAAYVNNLNVTEEKYQEALAKGD
VTAQIALQPALKFNGGGHINHSIFWTNLSPNGGGEPKGELLEAIKRDFGSFDKFKEKLTAASVGVQGSGWGWLGFNKE
RGHLQIAACPNQDPLQGTTGLIPLLGIDVWEHAYYLQYKNVRPDYLKAIWNVINWENVTERYMACKK

96 hr membrane fraction/Spot 8/Vimentin/Mascot
MSTRSVSSSSYRRMFGGPGTASRPSSSRSYVTTSTRTYSLGSALRPSTSRSLYASSPGGVYATRSSAVRLRSSVPGVRLLQ
DSVDFSLADAINTEFKNTRTNEKVELQELNDRFANYIDKVRFLEQQNKILLAELEQLKGQGKSRLGDLYEEEMRELRRQ
VDQLTNDKARVEVERDNLAEDIMRLREKLQEEMLQREEAENTLQSFRQDVDNASLARLDLERKVESLQEEIAFLKKLH
EEEIQELQAQIQEQHVQIDVDVSKPDLTAALRDVRQQYESVAAKNLQEAEEWYKSKFADLSEAANRNNDALRQAKQE
STEYRRQVQSLTCEVDALKGTNESLERQMREMEENFAVEAANYQDTIGRLQDEIQNMKEEMARHLREYQDLLNVK
MALDIEIATYRKLLEGEESRISLPLPNFSSLNLRETNLDSLPLVDTHSKRTFLIKTVETRDGQVINETSQHHDDLE

96 hr membrane fraction/Spot 8/Vimentin/X!Hunter
MSTRSVSSSSYRRMFGGPGTASRPSSSRSYVTTSTRTYSLGSALRPSTSRSLYASSPGGVYATRSSAVRLRSSVPGVRLLQ
DSVDFSLADAINTEFKNTRTNEKVELQELNDRFANYIDKVRFLEQQNKILLAELEQLKGQGKSRLGDLYEEEMRELRRQV
DQLTNDKARVEVERDNLAEDIMRLREKLQEEMLQREEAENTLQSFRQDVDNASLARLDLERKVESLQEEIAFLKKLHEE
EIQELQAQIQEQHVQIDVDVSKPDLTAALRDVRQQYESVAAKNLQEAEEWYKSKFADLSEAANRNNDALRQAKQESTE
YRRQVQSLTCEVDALKGTNESLERQMREMEENFAVEAANYQDTIGRLQDEIQNMKEEMARHLREYQDLLNVKMALD
IEIATYRKLLEGEESRISLPLPNFSSLNLRETNLDSLPLVDTHSKRTLLIKTVETRDGQVINETSQHHDDLE

96 hr membrane fraction/Spot 8/Vimentin/X!Tandem P3
MSTRSVSSSSYRRMFGGPGTASRPSSSRSYVTTSTRTYSLGSALRPSTSRSLYASSPGGVYATRSSAVRLRSSVPGVRLLQ
DSVDFSLADAINTEFKNTRTNEKVELQELNDRFANYIDKVRFLEQQNKILLAELEQLKGQGKSRLGDLYEEEMRELRRQV
DQLTNDKARVEVERDNLAEDIMRLREKLQEEMLQREEAENTLQSFRQDVDNASLARLDLERKVESLQEEIAFLKKLHEE
EIQELQAQIQEQHVQIDVDVSKPDLTAALRDVRQQYESVAAKNLQEAEEWYKSKFADLSEAANRNNDALRQAKQESTE
YRRQVQSLTCEVDALKGTNESLERQMREMEENFAVEAANYQDTIGRLQDEIQNMKEEMARHLREYQDLLNVKMALD
IEIATYRKLLEGEESRISLPLPNFSSLNLRETNLDSLPLVDTHSKRTLLIKTVETRDGQVINETSQHHDDLE

96 hr membrane fraction/Spot 9/Vimentin/Mascot
MSTRSVSSSSYRRMFGGPGTASRPSSSRSYVTTSTRTYSLGSALRPSTSRSLYASSPGGVYATRSSAVRLRSSVPGVRLLQ
DSVDFSLADAINTEFKNTRTNEKVELQELNDRFANYIDKVRFLEQQNKILLAELEQLKGQGKSRLGDLYEEEMRELRRQ
VDQLTNDKARVEVERDNLAEDIMRLREKLQEEMLQREEAENTLQSFRQDVDNASLARLDLERKVESLQEEIAFLKKLH
EEEIQELQAQIQEQHVQIDVDVSKPDLTAALRDVRQQYESVAAKNLQEAEEWYKSKFADLSEAANRNNDALRQAKQE
STEYRRQVQSLTCEVDALKGTNESLERQMREMEENFAVEAANYQDTIGRLQDEIQNMKEEMARHLREYQDLLNVK
MALDIEIATYRKLLEGEESRISLPLPNFSSLNLRETNLDSLPLVDTHSKRTFLIKTVETRDGQVINETSQHHDDLE

96 hr membrane fraction/Spot 9/Vimentin/X!Hunter
MSTRSVSSSSYRRMFGGPGTASRPSSSRSYVTTSTRTYSLGSALRPSTSRSLYASSPGGVYATRSSAVRLRSSVPGVRLLQ
DSVDFSLADAINTEFKNTRTNEKVELQELNDRFANYIDKVRFLEQQNKILLAELEQLKGQGKSRLGDLYEEEMRELRRQV
DQLTNDKARVEVERDNLAEDIMRLREKLQEEMLQREEAENTLQSFRQDVDNASLARLDLERKVESLQEEIAFLKKLHEE
EIQELQAQIQEQHVQIDVDVSKPDLTAALRDVRQQYESVAAKNLQEAEEWYKSKFADLSEAANRNNDALRQAKQESTE
YRRQVQSLTCEVDALKGTNESLERQMREMEENFAVEAANYQDTIGRLQDEIQNMKEEMARHLREYQDLLNVKMALD
IEIATYRKLLEGEESRISLPLPNFSSLNLRETNLDSLPLVDTHSKRTLLIKTVETRDGQVINETSQHHDDLE

96 hr membrane fraction/Spot 9/Vimentin/X!Tandem P3
MSTRSVSSSSYRRMFGGPGTASRPSSSRSYVTTSTRTYSLGSALRPSTSRSLYASSPGGVYATRSSAVRLRSSVPGVRLLQ
DSVDFSLADAINTEFKNTRTNEKVELQELNDRFANYIDKVRFLEQQNKILLAELEQLKGQGKSRLGDLYEEEMRELRRQV
DQLTNDKARVEVERDNLAEDIMRLREKLQEEMLQREEAENTLQSFRQDVDNASLARLDLERKVESLQEEIAFLKKLHEE
EIQELQAQIQEQHVQIDVDVSKPDLTAALRDVRQQYESVAAKNLQEAEEWYKSKFADLSEAANRNNDALRQAKQESTE
YRRQVQSLTCEVDALKGTNESLERQMREMEENFAVEAANYQDTIGRLQDEIQNMKEEMARHLREYQDLLNVKMALD
IEIATYRKLLEGEESRISLPLPNFSSLNLRETNLDSLPLVDTHSKRTLLIKTVETRDGQVINETSQHHDDLE

96 hr membrane fraction/Spot 10/Rab7/Mascot
MTSRKKVLLKVIILGDSGVGKTSLMNQYVNKKFSNQYKATIGADFLTKEVMVDDRLVTMQIWDTAGQERFQSLGVAF
YRGADCCVLVFDVTAPNTFKTLDSWRDEFLIQASPRDPENFPFVVLGNKIDLENRQVATKRAQAWCYSKNNIPYFETS
AKEAINVEQAFQTIARNALKQETEVELYNEFPEPIKLDKNDRAKASAESCSC

96 hr membrane fraction/Spot 10/Rab7/X!Hunter
MTSRKKVLLKVIILGDSGVGKTSLMNQYVNKKFSNQYKATIGADFLTKEVMVDDRLVTMQIWDTAGQERFQSLGVAF
YRGADCCVLVFDVTAPNTFKTLDSWRDEFLIQASPRDPENFPFVVLGNKIDLENRQVATKRAQAWCYSKNNIPYFETSA
KEAINVEQAFQTIARNALKQETEVELYNEFPEPIKLDKNDRAKASAESCSC

96 hr membrane fraction/Spot 10/Rab7/X!Tandem P3
MTSRKKVLLKVIILGDSGVGKTSLMNQYVNKKFSNQYKATIGADFLTKEVMVDDRLVTMQIWDTAGQERFQSLGVAF
YRGADCCVLVFDVTAPNTFKTLDSWRDEFLIQASPRDPENFPFVVLGNKIDLENRQVATKRAQAWCYSKNNIPYFETSA
KEAINVEQAFQTIARNALKQETEVELYNEFPEPIKLDKNDRAKASAESCSC

96 hr membrane fraction/Spot 18/Rab7/Mascot
MTSRKKVLLKVIILGDSGVGKTSLMNQYVNKKFSNQYKATIGADFLTKEVMVDDRLVTMQIWDTAGQERFQSLGVAF
YRGADCCVLVFDVTAPNTFKTLDSWRDEFLVQASPRDPENFPFVVLGNKVDLENRQVATKRAQAWCYSKNNIPYFET
SAKEAINVEQAFQTIARNALKQETEVELYNEFPEPIKLDKNDRAKASAESCSC

96 hr membrane fraction/Spot 18/Rab7/X!Hunter
MTSRKKVLLKVIILGDSGVGKTSLMNQYVNKKFSNQYKATIGADFLTKEVMVDDRLVTMQIWDTAGQERFQSLGVAF
YRGADCCVLVFDVTAPNTFKTLDSWRDEFLIQASPRDPENFPFVVLGNKIDLENRQVATKRAQAWCYSKNNIPYFETSA
KEAINVEQAFQTIARNALKQETEVELYNEFPEPIKLDKNDRAKASAESCSC

96 hr membrane fraction/Spot 18/Rab7/X!Tandem P3
MTSRKKVLLKVIILGDSGVGKTSLMNQYVNKKFSNQYKATIGADFLTKEVMVDDRLVTMQIWDTAGQERFQSLGVAF
YRGADCCVLVFDVTAPNTFKTLDSWRDEFLIQASPRDPENFPFVVLGNKIDLENRQVATKRAQAWCYSKNNIPYFETSA
KEAINVEQAFQTIARNALKQETEV

96 hr membrane fraction/Spot 20/Vimentin/Mascot
MSTRSVSSSSYRRMFGGPGTASRPSSSRSYVTTSTRTYSLGSALRPSTSRSLYASSPGGVYATRSSAVRLRSSVPGVRLLQ
DSVDFSLADAINTEFKNTRTNEKVELQELNDRFANYIDKVRFLEQQNKILLAELEQLKGQGKSRLGDLYEEEMRELRRQV
DQLTNDKARVEVERDNLAEDIMRLREKLQEEMLQREEAENTLQSFRQDVDNASLARLDLERKVESLQEEIAFLKKLHEE
EIQELQAQIQEQHVQIDVDVSKPDLTAALRDVRQQYESVAAKNLQEAEEWYKSKFADLSEAANRNNDALRQAKQESTE
YRRQVQSLTCEVDALKGTNESLERQMREMEENFAVEAANYQDTIGRLQDEIQNMKEEMARHLREYQDLLNVKMALD
IEIATYRKLLEGEESRISLPLPNFSSLNLRETNLDSLPLVDTHSKRTFLIKTVETRDGQVINETSQHHDDLE

96 hr membrane fraction/Spot 20/Vimentin/X!Hunter
MSTRSVSSSSYRRMFGGPGTASRPSSSRSYVTTSTRTYSLGSALRPSTSRSLYASSPGGVYATRSSAVRLRSSVPGVRLLQ
DSVDFSLADAINTEFKNTRTNEKVELQELNDRFANYIDKVRFLEQQNKILLAELEQLKGQGKSRLGDLYEEEMRELRRQV
DQLTNDKARVEVERDNLAEDIMRLREKLQEEMLQREEAENTLQSFRQDVDNASLARLDLERKVESLQEEIAFLKKLHEE
EIQELQAQIQEQHVQIDVDVSKPDLTAALRDVRQQYESVAAKNLQEAEEWYKSKFADLSEAANRNNDALRQAKQESTE
YRRQVQSLTCEVDALKGTNESLERQMREMEENFAVEAANYQDTIGRLQDEIQNMKEEMARHLREYQDLLNVKMALD
IEIATYRKLLEGEESRISLPLPNFSSLNLRETNLDSLPLVDTHSKRTFLIKTVETRDGQVINETSQHHDDLE

96 hr membrane fraction/Spot 20/Vimentin/X!Tandem P3
MSTRSVSSSSYRRMFGGPGTASRPSSSRSYVTTSTRTYSLGSALRPSTSRSLYASSPGGVYATRSSAVRLRSSVPGVRLLQ
DSVDFSLADAINTEFKNTRTNEKVELQELNDRFANYIDKVRFLEQQNKILLAELEQLKGQGKSRLGDLYEEEMRELRRQV
DQLTNDKARVEVERDNLAEDIMRLREKLQEEMLQREEAENTLQSFRQDVDNASLARLDLERKVESLQEEIAFLKKLHEE
EIQELQAQIQEQHVQIDVDVSKPDLTAALRDVRQQYESVAAKNLQEAEEWYKSKFADLSEAANRNNDALRQAKQESTE
YRRQVQSLTCEVDALKGTNESLERQMREMEENFAVEAANYQDTIGRLQDEIQNMKEEMARHLREYQDLLNVKMALD
IEIATYRKLLEGEESRISLPLPNFSSLNLRETNLDSLPLVDTHSKRTLLIKTVETRDGQVINETSQHHDDLE

96 hr membrane fraction/Spot 28/Hsp60/Mascot
MLRLPTVFRQMRPVSRVLAPHLTRAYAKDVKFGADARALMLQGVDLLADAVAVTMGPKGRTVIIEQSWGSPKVTKD
GVTVAKSIDLKDKYKNIGAKLVQDVANNTNEEAGDGTTTATVLARSIAKEGFEKISKGANPVEIRRGVMLAVDAVIAEL
KKQSKPVTTPEEIAQVATISANGDKEIGNIISDAMKKVGRKGVITVKDGKTLNDELEIIEGMKFDRGYISPYFINTSKGQK
CEFQDAYVLLSEKKISSIQSIVPALEIANAHRKPLVIIAEDVDGEALSTLVLNRLKVGLQVVAVKAPGFGDNRKNQLKDM
AIATGGAVFGEEGLTLNLEDVQPHDLGKVGEVIVTKDDAMLLKGKGDKAQIEKRIQEIIEQLDVTTSEYEKEKLNERLAK
LSDGVAVLKVGGTSDVEVNEKKDRVTDALNATRAAVEEGIVLGGGCALLRCIPALDSLTPANEDQKIGIEIIKRTLKIPA
MTIAKNAGVEGSLIVEKIMQSSSEVGYDAMAGDFVNMVEKGIIDPTKVVRTALLDAAGVASLLTTAEVVVTEIPKEEKD
PGMGAMGGMGGGMGGGMF

96 hr membrane fraction/Spot 28/Hsp60/X!Hunter
MLRLPTVFRQMRPVSRVLAPHLTRAYAKDVKFGADARALMLQGVDLLADAVAVTMGPKGRTVIIEQSWGSPKVTKD
GVTVAKSIDLKDKYKNIGAKLVQDVANNTNEEAGDGTTTATVLARSIAKEGFEKISKGANPVEIRRGVMLAVDAVIAELK
KQSKPVTTPEEIAQVATISANGDKEIGNIISDAMKKVGRKGVITVKDGKTLNDELEIIEGMKFDRGYISPYFINTSKGQKCE
FQDAYVLLSEKKISSIQSIVPALEIANAHRKPLVIIAEDVDGEALSTLVLNRLKVGLQVVAVKAPGFGDNRKNQLKDMAIA
TGGAVFGEEGLTLNLEDVQPHDLGKVGEVIVTKDDAMLLKGKGDKAQIEKRIQEIIEQLDVTTSEYEKEKLNERLAKLSD
GVAVLKVGGTSDVEVNEKKDRVTDALNATRAAVEEGIVLGGGCALLRCIPALDSLTPANEDQKIGIEIIKRTLKIPAMTIAK
NAGVEGSLIVEKIMQSSSEVGYDAMAGDFVNMVEKGIIDPTKVVRTALLDAAGVASLLTTAEVVVTEIPKEEKDPGMG
AMGGMGGGMGGGMF

96 hr membrane fraction/Spot 28/Hsp60/X!Tandem P3
MLRLPTVFRQMRPVSRVLAPHLTRAYAKDVKFGADARALMLQGVDLLADAVAVTMGPKGRTVIIEQSWGSPKVTKD
GVTVAKSIDLKDKYKNIGAKLVQDVANNTNEEAGDGTTTATVLARSIAKEGFEKISKGANPVEIRRGVMLAVDAVIAELK
KQSKPVTTPEEIAQVATISANGDKEIGNIISDAMKKVGRKGVITVKDGKTLNDELEIIEGMKFDRGYISPYFINTSKGQKCE
FQDAYVLLSEKKISSIQSIVPALEIANAHRKPLVIIAEDVDGEALSTLVLNRLKVGLQVVAVKAPGFGDNRKNQLKDMAIA
TGGAVFGEEGLTLNLEDVQPHDLGKVGEVIVTKDDAMLLKGKGDKAQIEKRIQEIIEQLDVTTSEYEKEKLNERLAKLSD
GVAVLKVGGTSDVEVNEKKDRVTDALNATRAAVEEGIVLGGGCALLRCIPALDSLTPANEDQKIGIEIIKRTLKIPAMTIAK
NAGVEGSLIVEKIMQSSSEVGYDAMAGDFVNMVEKGIIDPTKVVRTALLDAAGVASLLTTAEVVVTEIPKEEKDPGMG
AMGGMGGGMGGGMF

96 hr membrane fraction/Spot 97/Enoyl CoA Hydratase 1/Mascot
MAAGIVASRRLRDLLTRRLTGSNYPGLSISLRLTGSSAQEEASGVALGEAPDHSYESLRVTSAQKHVLHVQLNRPNKRN
AMNKVFWREMVECFNKISRDADCRAVVISGAGKMFTAGIDLMDMASDILQPKGDDVARISWYLRDIITRYQETFNVI
ERCPKPVIAAVHGGCIGGGVDLVTACDIRYCAQDAFFQVKEVDVGLAADVGTLQRLPKVIGNQSLVNELAFTARKMM
ADEALGSGLVSRVFPDKEVMLDAALALAAEISSKSPVAVQSTKVNLLYSRDHSVAESLNYVASWNMSMLQTQDLVKS
VQATTENKELKTVTFSKL

96 hr membrane fraction/Spot 97/Enoyl CoA Hydratase 1/X!Hunter
MAAGIVASRRLRDLLTRRLTGSNYPGLSISLRLTGSSAQEEASGVALGEAPDHSYESLRVTSAQKHVLHVQLNRPNKRNA
MNKVFWREMVECFNKISRDADCRAVVISGAGKMFTAGIDLMDMASDILQPKGDDVARISWYLRDIITRYQETFNVIER
CPKPVIAAVHGGCIGGGVDLVTACDIRYCAQDAFFQVKEVDVGLAADVGTLQRLPKVIGNQSLVNELAFTARKMMAD
EALGSGLVSRVFPDKEVMLDAALALAAEISSKSPVAVQSTKVNLLYSRDHSVAESLNYVASWNMSMLQTQDLVKSVQA
TTENKELKTVTFSKL

96 hr membrane fraction/Spot 97/Enoyl CoA Hydratase 1/X!Tandem P3
MAAGIVASRRLRDLLTRRLTGSNYPGLSISLRLTGSSAQEEASGVALGEAPDHSYESLRVTSAQKHVLHVQLNRPNKRNA
MNKVFWREMVECFNKISRDADCRAVVISGAGKMFTAGIDLMDMASDILQPKGDDVARISWYLRDIITRYQETFNVIER
CPKPVIAAVHGGCIGGGVDLVTACDIRYCAQDAFFQVKEVDVGLAADVGTLQRLPKVIGNQSLVNELAFTARKMMAD
EALGSGLVSRVFPDKEVMLDAALALAAEISSKSPVAVQSTKVNLLYSRDHSVAESLNYVASWNMSMLQTQDLVKSVQA
TTENKELKTVTFSKL

96 hr soluble fraction/Spot 122/Aldehyde dehydrogenase 2/Mascot
MLRAAARFGPRLGRRLLSAAATQAVPAPNQQPEVFCNQIFINNEWHDAVSRKTFPTVNPSTGEVICQVAEGDKEDVD
KAVKAARAAFQLGSPWRRMDASHRGRLLNRLADLIERDRTYLAALETLDNGKPYVISYLVDLDMVLKCLRYYAGWADK
YHGKTIPIDGDFFSYTRHEPVGVCGQIIPWNFPLLMQAWKLGPALATGNVVVMKVAEQTPLTALYVANLIKEAGFPPG
VVNIVPGFGPTAGAAIASHEDVDKVAFTGSTEIGRVIQVAAGSSNLKRVTLELGGKSPNIIMSDADMDWAVEQAHFAL
FFNQGQCCCAGSRTFVQEDIYDEFVERSVARAKSRVVGNPFDSKTEQGPQVDETQFKKILGYINTGKQEGAKLLCGGGI
AADRGYFIQPTVFGDVQDGMTIAKEEIFGPVMQILKFKTIEEVVGRANNSTYGLAAAVFTKDLDKANYLSQALQAGTV
WVNCYDVFGAQSPFGGYKMSGSGRELGEYGLQAYTEVKTVTVKVPQKNS

96 hr soluble fraction/Spot 122/Aldehyde dehydrogenase 2/X!Hunter
MLRAAARFGPRLGRRLLSAAATQAVPAPNQQPEVFCNQIFINNEWHDAVSRKTFPTVNPSTGEVICQVAEGDKEDVD
KAVKAARAAFQLGSPWRRMDASHRGRLLNRLADLIERDRTYLAALETLDNGKPYVISYLVDLDMVLKCLRYYAGWADK
YHGKTIPIDGDFFSYTRHEPVGVCGQIIPWNFPLLMQAWKLGPALATGNVVVMKVAEQTPLTALYVANLIKEAGFPPG
VVNIVPGFGPTAGAAIASHEDVDKVAFTGSTEIGRVIQVAAGSSNLKRVTLELGGKSPNIIMSDADMDWAVEQAHFAL
FFNQGQCCCAGSRTFVQEDIYDEFVERSVARAKSRVVGNPFDSKTEQGPQVDETQFKKILGYINTGKQEGAKLLCGGGI
AADRGYFIQPTVFGDVQDGMTIAKEEIFGPVMQILKFKTIEEVVGRANNSTYGLAAAVFTKDLDKANYLSQALQAGTV
WVNCYDVFGAQSPFGGYKMSGSGRELGEYGLQAYTEVKTVTVKVPQKNS

96 hr soluble fraction/Spot 122/Aldehyde dehydrogenase 2/X!Tandem P3
MLRAAARFGPRLGRRLLSAAATQAVPAPNQQPEVFCNQIFINNEWHDAVSRKTFPTVNPSTGEVICQVAEGDKEDVD
KAVKAARAAFQLGSPWRRMDASHRGRLLNRLADLIERDRTYLAALETLDNGKPYVISYLVDLDMVLKCLRYYAGWADK
YHGKTIPIDGDFFSYTRHEPVGVCGQIIPWNFPLLMQAWKLGPALATGNVVVMKVAEQTPLTALYVANLIKEAGFPPG
VVNIVPGFGPTAGAAIASHEDVDKVAFTGSTEIGRVIQVAAGSSNLKRVTLELGGKSPNIIMSDADMDWAVEQAHFAL
FFNQGQCCCAGSRTFVQEDIYDEFVERSVARAKSRVVGNPFDSKTEQGPQVDETQFKKILGYINTGKQEGAKLLCGGGI
AADRGYFIQPTVFGDVQDGMTIAKEEIFGPVMQILKFKTIEEVVGRANNSTYGLAAAVFTKDLDKANYLSQALQAGTV
WVNCYDVFGAQSPFGGYKMSGSGRELGEYGLQAYTEVKTVTVKVPQKNS
