# Supplementary material for: Proteomic and Systems Biology Analysis of the Monocyte Response to Coxiella burnetii Infection
Source: PLoS One. 2013 Aug 21;8(8):e69558. doi: 10.1371/journal.pone.0069558 (PMC3749201; doi:10.1371/journal.pone.0069558)
Supplement: Methods S1 — Additional methodology information for this investigation. Additional information on the methods used in this investigation. References cited are listed in the references section of the manuscript. (DOC) [file pone.0069558.s008.doc]

S**upplemental methods:** The Monomac I cells were grown, and infected with phase II *C. burnetii* using previously established techniques [14]. Pelleted cells were resuspended to 4 mL final volume ( 5x10^7^ cells/mL), using monocyte extraction buffer [160 mM NaCl (ACS grade, Fisher), 10 mM MgCl_2_·6H_2_O (ACS grade, Baker), 1 mM Na_3_VO_3_ (ultra pure research grade, LC laboratories), 5 mM NaF (ultra pure research grade, LC laboratories), 20 mM Tris pH 7.5 (electrophoresis grade, Biorad), and 1 Roche Complete protease inhibitor cocktail tablet per 50 mL buffer) [15-17] and mixed thoroughly. Pooled cells were divided into four 1 mL aliquots for homogenization, sonication, ultracentrifugation, and protein precipitation.

One ml aliquots of resuspended cell samples were lysed on ice with 400 passes in a hand driven Dounce homogenizer, 4 rounds of sonication on ice, a freeze thaw cycle to -80 °C, followed by 2 rounds of sonication on ice. Sonication was performed using fifteen 1 second bursts with 1 second pauses between each burst, using a Fisher Scientific Sonic Dismembranator model 100 on setting 3, a Misonix Inc. ultrasonic converter and a microtip. Between rounds of sonication, samples were held on ice for at least 1 minute. Lysed samples were subjected to high speed centrifugation (1x 45 minutes, 100,000 xg, 4 °C). The soluble fraction, primarily consisting of cytosolic proteins and the luminal contents of organelles and the membrane fraction containing the plasma and organellar membranes. The samples were stored at -80 °C until further processing [19].

Trichloroacetic acid precipitation

The soluble protein fraction was precipitated with trichloroacetic acid (TCA) (ACS grade, Fisher) followed by acetone washes. Briefly, freshly made 100% (w/v) TCA was added to a final concentration of 10% (v/v) in each of the 1 mL aliquots, vortexed briefly and incubated on ice for 1 hour. Precipitates were pelleted at 6800 xg 4 °C in a Legend Sorvall Mach 1.6R centrifuge for 1 minute, and the supernatant was discarded. To each protein pellet was added 800 uL of -20 °C acetone (Optima grade, Fisher) and the pellet was broken up by sonication and vortexing (as above, on setting 2). The pellets were incubated overnight at -20 °C. The samples were centrifuged as above, the supernatant discarded and the pellet washed twice more (at -20 °C) for 30 minutes each with fresh acetone each time. Following the final wash the supernatant was discarded and the pellet Speed-vac'd to complete dryness. To each dried pellet was added 200 uL of labeling buffer [7M urea (Sigma, ultra grade), 2M thiourea (Sigma, ACS grade), 30 mM Tris, 4% (w/v) CHAPS (Sigma, electrophoresis grade), 1 mM Na_3_VO_3_, 5 mM NaF, and 1 tablet Roche Complete protease inhibitor cocktail/50 mL buffer]. Like pellets were pooled, and either stored at -80 °C or quantified by the Bradford protein assay (Biorad).

Bradford assay

The Bradford assay and pH adjustments were performed immediately prior to labeling.

Label buffer was added to the samples as needed to adjust the protein concentration to ~10-15 ug/uL. After adjusting the concentration of the sample, the pH was adjusted to 8.4-8.5. The pH was adjusted in small increments, so as not to overshoot the target range, in order to minimize the introduction of excess salts.

Once the pH had been adjusted, the proteins were quantified by the Bradford assay using BSA (BioRad Quick Start BSA protein standard catalog# 500-0206) for the standard curve. The BSA measurements were made in triplicate, and an average value was used for the standard curve. Five sample replicates were measured for each sample to be tested. The samples were vortexed well, and equilibrated for 15 minutes at room temperature. 200 uL per tube was transferred to a 96 well plate and scanned on a Tecan Safire at 595 nm, using 3 flashes / read.

The standard curve was calculated for the BSA sample measurements, an average taken of the A595 readings, and a linear best fit curve plotted (y=mx+b) vs. the [BSA]. A minimum of five points were required to generate the working calibration curve. If a minimum of five points could not be generated, the assay was rerun. To assess the quality of the data, the average of the unknown sample A595 values +/- 10% of the average A595 value for that sample was calculated and outliers discarded. Each sample average was calculated with a minimum of 3 results.

Labeling for multiplex differential 2DE

The methods have been adapted from published 2D-gel [24] and 2D-DIGE protocols[GE Healthcare CyDye Manual]. All steps were performed at room temperature, unless otherwise indicated. Following the Bradford assay, aliquots of samples were prepared in the following manner:

Control: volume of sample to obtain 100 ug protein

Experimental: volume of sample to obtain 100 ug protein

Pooled internal standard: 1/2 the volume of sample of each of the above two samples (50 ug per sample).

Forward labeling was always made with the control sample labeled with ZGB (green fluorophore), experimental samples labeled with ZBB (blue fluorophore), and the pooled internal standard labeled with ZRB (red fluorophore) [21-23]. For reverse labeling, control and experimental labeling colors were reversed. ZRB was always used to label the internal standard. All samples were >5 mg/mL in their stock vial. The samples were brought to 20 uL (5 mg/mL final concentration) with labeling buffer pH 8.5 containing detergent(s) appropriate for the sample type. Each technical replicate was labeled separately.

For a labeling reaction, 4 nmol of dye per reaction (ZGB, ZBB, or ZRB) was added to the protein aliquot at a dye stock concentration of 1 nmol/uL. Samples were vortexed well and centrifuged briefly to pull liquid to bottom of the tubes. Samples were allowed to react with the Zdyes for 30 minutes in the dark at room temperature. 5 uL of 10 mM lysine was added to quench the reaction, the tubes were vortexed and centrifuged briefly as above, and incubated for 10 minutes in the dark. The fractions were combined appropriately, 1 green + 1 red + 1 blue in appropriate labeling condition (all forward label or all reverse) and 8.44 uL of 1.1 M DTT (20 mM final concentration), 2.25 uL of carrier ampholytes, labeling buffer to final volume (450 uL final volume for a 24 cm strip) and 3 uL of 0.03% (w/v) bromophenol blue were added. Samples were vortexed well and centrifuged briefly.

IEF was achieved using 24 cm immobilized pH gradient (IPG) strips pH 3-11NL. The samples were loaded via active rehydration using a Biorad Protean IEF cell at 50V, 20 °C for 14-16 hours, with a layer of mineral oil over the strip to prevent drying. To facilitate even loading of the strips, the sample was evenly distributed across the length of the strip. Note that wicks (BioRad Electrode Wicks catalog# 165-4071) were placed at the ends of each strip to ensure proper contact with the tray electrodes and to help ensure that the sample stayed in contact with the strip. A complete sample set for a single biological replicate (3x forward label strips, and 3x reverse label strips) were always run simultaneously. After loading for 14-16 hours, the strips were distributed as needed to IPGphor instruments such that only one sample type per labeling condition was present on a given IPGphor. Strips were stored at -80 °C until run on the 2nd dimension separation.

2nd dimension

Acrylamide gels were cast using 1.5mm spacer plates and the Amersham/GE Healthcare casting chamber. The 16% (v/v) acrylamide [0.3M Tris pH 8.8, 16% (v/v) bisacrylamide 37.5:1 (2.6% C), 19.8% (v/v) glycerol] was used for membrane samples and the 18% (v/v) acrylamide solution [0.3M Tris pH 8.8, 18% (v/v) bisacrylamide 37.5:1 (2.6% C), 17.4% (v/v) glycerol] was used for soluble fraction samples. The samples were degassed 1x 25 minutes under a vacuum in a sonicator bath, starting with the high %acrylamide sample. After degassing, solutions were stored at 4 °C until ready to pour the gels, as the vacuum flasks got warm at the top during degassing, and heat transfer would speed the polymerization process, possibly causing nonuniform polymerization. The degassing was repeated for the 9.5% (v/v) acrylamide solution [0.3M Tris pH 8.8, 9.5% (v/v) bisacrylamide 37.5:1 (2.6% C)] and the flasks containing the acrylamide solutions were stored at 4 °C until ready to pour the gels.

All gels contained a unique identifier, containing first and last initial and a unique five digit number (e.g. MSxxxxx). All gel plates were wiped thoroughly with 70% (v/v) ethanol and a lint-free cloth prior to layup. Gels were cast in the Amersham 24 cm casting chamber (13 gels/casting). Immediately prior to casting, the gradient maker was rinsed with 500 mL of nanopure water per chamber. 10% (w/v) APS and TEMED was added from stock containers to final concentrations of 0.016% (v/v) for APS and 0.016% (v/v) for TEMED for each acrylamide solution, and swirled gently to mix. Casting continued until all of the acrylamide had been pumped into the casting chamber, including the solution in the tubing. Displacement buffer [0.3M Tris pH 8.8, 50% (v/v) glycerol, trace bromophenol blue] was added slowly until the top of the acrylamide just reached the top of the spacers. The displacement buffer remained below the bottom of the gel plates. Each gel was gently covered with 3.5 mL of water saturated butanol per gel, starting at one end of the gel and working towards the other. The gels were allowed to polymerize overnight on the bench at room temperature. The following morning, the caster was opened and each gel cassette rinsed with nanopure water, placed in 1x running buffer [(0.1% (w/v) SDS, 0.192M glycine, and 0.025M Tris)], and stored at 4 °C.

Prior to loading the gels, IPG strips were thawed and reduced for15 minutes in 7 mL/strip of 0.13M dithiothretol (DTT) in equilibration buffer [6M urea, 4%(w/v) SDS, 30% (v/v) glycerol, and 500 mM Tris pH 8.8], followed by alkylation for 15 minutes in 7 mL/strip of 0.51M iodoacetamide (IAA) in equilibration buffer [24, 28, 29]. The IPG strips were loaded onto 2nd dimension gels under a layer of 0.5% (w/v) agarose and a trace of bromophenol blue. Gel cassettes were run in a Dalt12 tank overnight in 1x running buffer at 16 °C in the dark at 4W/gel (44-48W total for the duration of the run, typically 18-20 hours). When the bromophenol blue front exited the gel the gels were removed from the tank and placed in fixative for ~28 hrs. in the dark on a shaker table. The fixative solution was carefully removed from the gels as completely as possible, replaced with 1-1.5 L of nanopure water, and placed on a shaker table for 1 hour in the dark. The wash was repeated once more by removing the wash water and adding fresh nanopure water (1-1.5 L), followed by incubating overnight on a shaker table in the dark prior to scanning the following morning. Following the overnight wash in nanopure water, the gels were scanned on a Typhoon Trio gel scanner at 200 um resolution, as described in the text. Gels were scanned as close to saturation as possible without actually producing saturated pixels in the viewable/analyzable area of the gel. Saturated pixels in this region required a replicate scan at a lower PMT setting.

Gel analysis

Images were uploaded into the Progenesis software package (v. 2 Nonlinear Dynamics). The Progenesis software was set to the multiple dyes without DIGE structure setting. Gel images were labeled uniquely and a reference image was chosen that had the best qualitative appearance with respect to highest spot number, and best spot separation. The area of interest, the portion of the gel being analyzed, was selected and aligned with different colors on the same gel and aligned between gels. Gels were aligned first by manually aligning many of the most intense spots as well as some of the more distinctive less intense spots. This was followed by automatic vector detection. Alignment was manually validated and adjusted where needed for each gel.

Following alignment and spot detection, regions of the gel that were too streaky or had a high background (obscuring details in the main body of the gel) were excluded. A minimum of 2 technical replicates were required for each forward and reverse labeling condition in a particular biological replicate (minimum of 4 gels total). In group setup, all control gels were grouped together, and all experimental gels were grouped together. In the view results portion, every spot was manually examined to assess proper splitting and ensure that the software had not selected an overly large spot area, requiring trimming.

For the initial pass through the spots to evaluate differential expression, a p-value of <0.05 was required, and the spot area had to appear to contain a clear spot. Fold change did not play a part in decision making criteria. Selected spots that were properly split (either manually or by the software) and met the appropriate p-value cutoff proceeded to Progenesis stats analysis. Here the same p-value decision criteria as well as a power score of > 0.8 was required. For those spots passing this phase of statistical analysis, the data was exported to an Excel file. The data was analyzed by nested ANOVA (mixed linear model) as described in the text and in supplemetary materials. The Syntax for the nested ANOVA in table S2 is explained in the following: SAVE OUTFILE = is a unique command for each dataset that indicates the pathway for storage of the output of a particular analysis on the local computer. In the commands, /RANDOM indicates which condition in our data is random, in this case biological replicate. The command /METHOD=SSTYPE(3), indicates that this test uses a type 3 sum of squares. The command /INTERCEPT=INCLUDE indicates that the intercept term (a constant) is included in the calculations. The command /CRITERIA=ALPHA(.05) indicates a p-value cutoff of 0.05 /PRINT=OPOWER directs the program to return the observed power in addition to a p-value. /DESIGN specifes how the variables relate to each other. The output from the nested ANOVA analysis was utilized as described in the text.

Spot picking

Gels selected for picking were placed in a glass container filled with 1 L of Blue Silver Coomassie Blue [10% (v/v) phosphoric acid (ACS grade, Fisher), 10% (w/v) NH_4_SO_4_ (ACS grade, Sigma), 0.12% (w/v) Coomassie Blue G-250 (standard grade, Fluka), 20% (v/v) methanol (HPLC grade, Fisher)] [Candiano et al, 2004 ] per 3 gels, covered, and placed on a shaker overnight. The stain was gently removed as completely as possible by vacuum. Gels were rinsed twice with fresh nanopure water, removing liquid by vacuum each time. Destaining was done using several changes of nanopure water and shaking each wash for 1 hour to overnight until the gel background appeared to be gone. Spots were picked from the gel using a scalpel wiped with 95% ethanol before each spot was picked. Typically, corresponding spots from three gels were picked and pooled to maximize recovery.

In-gel protein digestion and MS analysis

Gel pieces were destained with 25 mM NH_4_HCO_3_/50% (v/v) acetonitrile, vortexed for 10 minutes, and centrifuged briefly to pellet the gel pieces and facilitate removal of the liquid. Fresh 25 mM NH_4_HCO_3_ (ReagentPlus grade, Sigma)/50% (v/v) acetonitrile (Optima grade, Fisher) was used for each wash. Pieces were washed until no longer blue (typically 2-4 washes). When all washes were complete, the gel pieces were dried completely in a Speed-vac.

Trypsin was activated according to manufacturer's instructions. The pieces were vortexed and sonicated as for the first extraction, and the remaining extractions were vortexed only. Each peptide extract were pooled with the previous extract. The peptide solutions were Speed-vac'd to <200 uL and transferred to labeled 0.6 mL tubes being careful to not transfer gel pieces in the process. The peptide extracts were Speed-vac'd to < 20 uL and 200 uL of fresh nanopure water was added. The solutions were then Speed-vac'd again to ~20 uL final volume and either stored at -80° or used immediately for MS analysis.

For MS analysis, the peptide extracts were carefully transferred to fresh tubes appropriate for the autosampler on an Agilent 1100 series HPLC. Care was exercised to prevent any remaining gel pieces from being transferred to the autosampler vial. All reagents used were HPLC grade. This search output file was exported as a .mgf file and used to identify the proteins, using bioinformatics tools, as described in the text.

Additional Reference:

Candiano G, Bruschi M, Musante L, Santucci L, et al. (2004) Blue silver: a very sensitive colloidal Coomassie G-250 staining for proteome analysis. Electrophoresis 25: 1327-1333.
